# The relationship between just world belief and wellbeing, cheating behaviors, and academic work behaviors during COVID 19 among university students

**DOI:** 10.1038/s41598-022-18045-7

**Published:** 2022-08-22

**Authors:** Susan Münscher

**Affiliations:** Department of Psychology, University of Cologne, Gronewaldstrasse 2, 50935 Cologne, Germany

**Keywords:** Health care, Human behaviour

## Abstract

Is the belief in a just world among students also stable under COVID-19? To answer this question, a study was conducted with university students from Germany (n = 291). The aim of the study was to analyze the predictive performance of the personal belief in a just world (PBJW) on students' life satisfaction and academic cheating and to take into account important mediators from the university context such as fellow student justice, lecturer justice, and procrastination. Derived from existing research, university students with a stronger PBJW should be more satisfied with their lives and cheat less than those with a weaker PBJW. The results support the hypothesized direct effects of PBJW on life satisfaction. Procrastination additionally mediated the effect of PBJW on life satisfaction. The level of PBJW predicted academic cheating only indirectly. The mediators procrastination and lecturer justice were crucial here. The results persisted when gender, learning, time to exam, socially desirable responding, general BJW, and self-efficacy were controlled. The findings were discussed in relation to the stressful situation caused by COVID-19. A reflection on the adaptive function of PBJW as a resource and relevant situation-specific mediators for university research and practice followed.

## Introduction

The COVID-19 pandemic changed the life conditions of all people^1^. Not only worry about a possible disease but also the university interventions to contain the number of infections affected the students^2^. Stresses associated with the COVID-19 pandemic can be considered a critical life event. A protective factor here is the personal belief in a just world (PBJW), which supports the maintenance of life satisfaction^3^. Behavior and experience changes have occurred during the COVID-19 pandemic^4^. Topics such as life satisfaction, procrastination, and cheating behavior in exam situations are possible research. Crucial to dealing with pandemic life situations and university regulations are available external and internal resources. Identifying resources in education that can protect students' life satisfaction and reduce maladaptive behavior is considered a research goal. According to previous research, personal belief in a just world (PBJW) should be a significant resource in this context^5,6^. PBJW describes the belief of justice in a person's own social environment^7^. The person assumes to be treated justly by his or her community. Correia et al.^3^ described that PBJW leads to higher life satisfaction. Research on dishonesty, delinquency, and academic cheating^8,9^ among high school and college students showed a significant negative association with PBJW. Consequently, people with a strong PBJW have better well-being and behave more justly toward their fellows than people with a weak PBJW.

In justice research, the effect of PBJW on the outcome variable is often mediated by the social environment^10,11^. Münscher et al.^8^ examined the interaction of university students' perceived lecturer and fellow student justice on life satisfaction and cheating. It was found that the relationship between PBJW and life satisfaction, as well as cheating, was partially mediated by individual perceptions of just behavior of lecturers and fellow students. One goal of the present study was to replicate the results during the COVID-19 pandemic. Supporting findings can be found in the school sector. Comparable results were found for life satisfaction^12^ as well as for various forms of delinquent behavior^9^. At the University of Cologne, only online teaching was offered until the winter semester 2021/2022, which conditions a reduced contact among university students or with lecturers. Whether these social contacts are also important as mediators during the COVID-19 pandemic will be investigated in the study. Outside of the external factors (lecturer justice and fellow student justice), the internal factor, university work behavior, could have an impact on life satisfaction and academic cheating. A suitable construct here is procrastination. Procrastination negatively affects life satisfaction^13^ and positively affects cheating^14^. Derived from a preliminary study^15^, a higher belief in a just world is associated with lower procrastinating behavior. Transferring to this study, procrastination should mediate the relationship between PBJW and life satisfaction as well as academic cheating. The function of the PBJW and its effect on the perception of the social environment is essential according to previous studies. To what extent this is also valid during COVID-19 pandemic is to be questioned.

Based on the hypothesized stability of PBJW, it is reasonable to assume that the relationship between PBJW and life satisfaction/academic cheating may be mediated in part by perceived lecturer justice and fellow student justice. This evidence should be supplemented by further research on procrastination.

### Belief in a just world

For one's own life management and planning, the conviction of a just living together is a central premise. The subjective assumption that the person lives in a just world and is treated accordingly by its social environment is the basis of the justice motive^16^. The basal cognitive schema described is referred to as the "Belief in a just world" (BJW). Following Lerner’s^16^ research and further research by Dalbert^17^, BJW functions as a resource. It is an anchor for experiencing justice under both unstressed^18^ and stressed^19,20^ living conditions. According to Lerner^16^, people have a need to believe in a just world where everyone gets what they deserve and deserves what they get. In response, researcher postulate that BJW is a disposition that varies across individuals.

Dalbert^17^ classifies BJW into three distinct functions that serve its maintenance: the assimilation function, the trust function, and the motivation function.

Through the person's trust in community and in the just actions of others^21^, which is described in the trust function, BJW serves to support well-being^22^, among other things. Furthermore, they described that with a higher belief in justice, distrust of others decreases. Dalbert^17^ saw this function as central to daily life. It can facilitate coping with everyday tasks and difficulties.

When injustice is experienced, the assimilation function acts to protect the BJW. Self-experienced or perceived injustice usually cannot be eliminated or reduced through action (e.g., compensation). Therefore, the experience of justice is cognitively restored (e.g., by minimizing or denying the injustice)^23^. In the school context, for example, students with a stronger BJW felt more justly treated by their teachers and classmates than those with a weaker BJW^24^. The same can be inferred for the university context^8^.

The motive function implies a commitment to act justly and to avoid one's own unjust behavior. Empirical evidence shows that students with a strong BJW behaved more justly toward their peers^25^ and were more motivated to achieve personal goals through just means. Münscher et al.^8^ found that university students with strong BJW reported less rule-breaking behavior such as academic cheating.

For further research, the differentiation of BJW into personal BJW (PBJW) and general BJW (GBJW) is important^7^. Dalbert et al.^26^ described the GBJW as a belief that things are just across the world. In contrast, PBJW includes the belief that things are generally just in one's own life. Researchers have shown that PBJW is better at explaining subjective well-being than GBJW and is also a better indicator of the justice motive^27,28^. Additionally, personal BJW was positively associated with prosocial behavior^29,30^ and forgiveness^30,31^ supported the findings. GBJW performs predictively for socially inappropriate behavior and harsh social attitudes (see Hafer and Sutton^32^, for a review). In addition, Bellomo et al.^33^ found a positive relationship between dishonest behavior and GBJW.

It is also exciting to look at the changeability of the two dimensions. Adoric^34^ explained the differential meaningfulness of experienced injustice for the two motive components. Long-lasting and recurrent unfair experiences, which are characterized by personal embeddedness, attack PBJW. In contrast, according to Adoric, these exert little influence on general just world beliefs. In the context of COVID-19, the study of PBJW is all the more important.

Therefore, the study used PBJW as the main predictor, controlling for the effects of GBJW. Through this, it may be possible to identify those of PBJW within life-critical events compared to GBJW.

### Personal belief in a just world and life satisfaction

According to Diener^35^, life satisfaction is defined as the cognitive and evaluative component of subjective well-being. A person's life is compared and evaluated against his or her own set standards. Situational conditions but also personality factors affect life satisfaction; at the university it is the established rules and situational factors^36^. Diener^35^ showed that critical life events significantly reduce life satisfaction, which can be assumed during the COVID-19 pandemic. Initial findings for increased anxiety levels and increased depression during the COVID-19 pandemic offer Satici et al.^37^. The psychological stress caused by COVID-19 led to a decrease in positive emotions and subjective well-being^38,39^.

PBJW and its functions serve as a personal protective factor for the maintenance of well-being^17,32^. Fundamentally, PBJW can be assumed to be related to life satisfaction^40^. In university students^41,42^, several studies showed a positive relationship between PBJW and life satisfaction when controlling for personality dimensions. A causal relationship of PBJW on life satisfaction was even found^3^.

Based on empirical findings^43^, PBJW could reduce the risk of stress-induced negative emotions and the development of depression due to the COVID-19 pandemic and increase well-being.

### Personal belief in a just world and academic cheating

Cheating is an act of deception that leads to personal gain by circumventing rules, standards, mores, and norms. Pegels^44^ considers it a form of deviant and illegal behavior in testing situations. Marsden et al.^45^ further describe typical cheating activities such as copying from other students, using cheat sheets, and obtaining exam content in advance. Whether academic cheating is considered normal depends on what happens in the classroom or at the university^46^. In principle, such behavior is inconsistent with the assumptions of BJW; in particular, the motive function obligates individuals to behave in a moral and socially acceptable manner. Any type of academic cheating violates the rules and norms of the university and affects the validity of examinations in higher education^47^. Academic cheating occurred more frequently during the COVID-19 pandemic^48^.

However, further study results showed that students with stronger PBJW beliefs behaved appropriately and according to the rules^9,49^. They were less likely to report cheating, less likely to engage in delinquent behavior, less likely to bully, and less likely to stay away from school than those with weak PBJW^9,10,25^. Some reinforcing findings also exist in the university context^8^.

The present study aims to examine the effect of PBJW on cheating among university students during the COVID-19 pandemic.

### Mediating role of justice experiences

Both life satisfaction and academic cheating research indicate the influence of social factors, such as the behavior of others^50^. According to just-world research, a direct relationship between PBJW and life satisfaction and academic cheating can be assumed, which explains much of the variance in research findings. Other effects of PBJW could be mediated by students' experiences with the social environment. Of interest are two groups that represent direct contacts for students, lecturers and fellow students^8^. Crucial here is the perception of justice-related behavior^51^.

Lecturer justice is defined as the individually and subjectively experienced justice of lecturers' behavior towards students (following teacher justice^52^). In contrast, the individually and subjectively experienced justice of the behavior of fellow students towards the student describes the fellow student justice according to Correia and Dalbert^51^. Fair treatment by lecturers and fellow students may also promote feelings of being valued and belonging, in line with the group value theory^53^, and have a positive impact on students' life satisfaction.

During COVID-19, contact with lecturers and fellow students changed. During the first lockdown, social contact was significantly limited in Germany. In many cases, digital contact with fellow students was established, which led to limitations in the learning environment and social networking^54^. Both the perception of justice-related behavior and the adaptation of justice-related behavior were significantly diminished by the digital medium. The lecturers' justice-related behaviors are also transparent at the digital level through the given rules and norms. However, the experience of fellow student justice was distorted by the lack of social contact^55^.

The study findings of Münscher et al.^8^ indicated a positive relationship between PBJW and lecturer justice as well as fellow student justice. Only fellow student justice served as a mediator of life satisfaction. Other supportive findings come from the school context. Here, perceived teacher justice^24^ and classmate justice^51^ mediated the relationship between students' PBJW and various indicators of well-being, including life satisfaction. That this influence can persist under COVID-19 is supported by the findings of Hafer et al.^56^. They reported a substantial influence of BJW on well-being. Bartholomaeus and Strelan^31^ also emphasized the significance of PBJW for well-being, coping with negative life events, prosocial behaviors, and a positive future orientation.

Studies of academic cheating supported the assumption of the mediating function of school and university justice experiences. For example, Donat et al.^9^ demonstrated that the negative relationships between students' PBJW and cheating were mediated in part by teacher justice and classmate justice. University students with strong beliefs in a just world who report less cheating perceived their lecturers' behavior to be more just than university students with low PBJW^8^.

Several studies supported that fair treatment in the university setting is significantly associated with lower levels of cheating among university students^57^. Based on the reduced interaction between students during digital teaching, it is reasonable to assume that the effect of PBJW on cheating is mediated only by lecturer justice.

### Mediating role of state-procrastination

Procrastination is mostly analyzed in the academic context. This is defined as the unnecessary postponement or not finishing of important activities or academic tasks, which can be explained by deficits in self-regulation/motivation^58^. A distinction is made between trait procrastination and state procrastination^59^. The first one is considered a personality disposition and thus occurs over a long period of time, whereas the second one occurs only situationally and in the short term^59^. Both types lead to a variety of academic problems, such as missing deadlines, insufficient exam preparation, feelings of guilt, low self-esteem, and social anxiety^59–63^. According to Chehrzad et al.^64^, 70% of university students procrastinate. During the COVID-19 pandemic, the risk of procrastinating is estimated to be even higher^65^. One of the motivations of procrastinating is the fear of negative evaluation and non-compliance^66,67^.

Starting points for a relationship between procrastination and PBJW can be derived from empirical evidence. In principle, PBJW functions as an adaptive resource in various demanding situations^11,17,23,68–71^. For example, students with higher PBJW are less likely to skip school, report lower test anxiety, and report fewer negative emotions than students with low PBJW^10^. Pursuing for long-term goals is also positively associated with PBJW^70–72^. Procrastinators have difficulty managing goals^73^ and exhibit marked anxiety^67^. Following the trust function of PBJWs, situational procrastination should occur less in the context of PBJW. Just world belief support coping with work-related stress^74^ and thus might be associated with a lower propensity for state procrastination. Preliminary evidence for this emerged in a pretest^15^.

Several empirical findings on procrastination and life satisfaction point to the negative effect of procrastination on life satisfaction^13,75^. Patrzek et al.^76^ investigated the relationship between all forms of academic cheating and procrastination among students. They found a significant positive correlation. Students who procrastinated reported cheating more. From the empirical findings, a mediating role between PBJW and life satisfaction/cheating can be suggested, which will be investigated in this study.

## Present study

**Figure 1:**
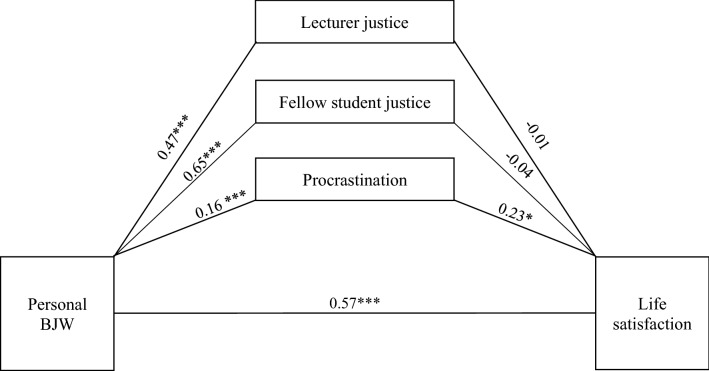
Mediation analysis of the relation between personal BJW and life satisfaction considering the control variables. Total indirect effect of procrastination (*b* = 0.04), lecturer justice (*b* = − 0.01) and fellow student justice (*b* = − 0.02) was: *b* = 0.00, 95% CI [− 0.09; 0.09]. Estimates are based on a bootstrapping procedure with 5000 bootstrap samples. *p < 0.05, ***p < 0.001. Used Process^77^ Model 4. Direct effects on the paths. Values describe unstandardized path coefficients.

**Figure 2:**
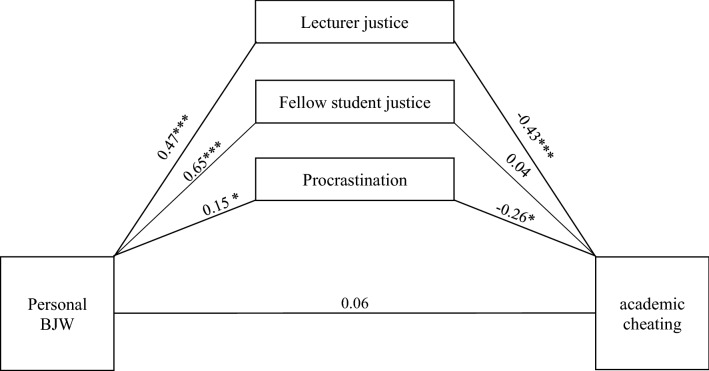
Mediation analysis of the relation between personal BJW and academic cheating considering the control variables. Total indirect effect of procrastination (*b* = − 0.04), lecturer justice (*b* = − 0.20) and fellow student justice (*b* = 0.03) was: *b* = − 0.21, 95% CI [− 0.35; − 0.08]. Estimates are based on a bootstrapping procedure with 5000 bootstrap samples. *p < 0.05, ***p < 0.001. Used Process^77^ Model 4. Direct effects on the paths. Values describe unstandardized path coefficients.

The aim of the present study is to replicate the study results of Münscher et al.^8^ during the COVID-19 pandemic. It is hypothesized that PBJW is positively related to students' life satisfaction and negatively related to cheating at university^3,9^. Recent research has considered mediators of the direct effect of PBJW on outcome variables that indirectly mediate the effect. Students' justice experiences with fellow students and lecturers play a significant role here^41^. However, during digital teaching due to COVID-19, a change in the perception of justice experiences can be assumed. Consequently, the influence of the mediator fellow student justice would have to be lower than during face-to-face teaching. By means of the study, on the one hand, the assumption of stability of PBJW and the maintenance of their adaptive functions during life-critical events should be supported, and on the other hand, the experience-based dependence of the perception of just behavior should be tested. Self-regulated work is considered one of the essential core competencies in digital teaching, which ranges from synchronous to asynchronous offerings at the University of Cologne. From empirical findings, it can be inferred that university work behaviors such as procrastination have a negative relationship with life satisfaction and a positive relationship with cheating. In justice research, it could serve as a mediator that mediates the effect of PBJW on cheating or life satisfaction. Based on the empirical findings, two path models can be derived by means of correlation hypotheses and mediator hypotheses (Figs. 1 and 2). The following hypotheses were tested:

1. The more students believed in a personal just world during COVID-19, the more satisfied they were with their lives.
2. The more students believed in a personal just world during COVID-19, the less they reported cheating in university.
3. Perceived just lecturer behavior mediates the relationships between students' PBJW and academic cheating.
4. Perceived just behavior of fellow students mediates the relationships between students' PBJW and their life satisfaction.
5. State-procrastination mediates the relationships between students' PBJW and their (5a) life satisfaction and (5b) academic cheating.

Furthermore, we expected these relationships to be significant when we controlled for confounding effects of gender, GBJW, academic self-efficacy, social desirability, distance to exam, and exam preparation.

### Control factors

There are ambiguous findings in BJW research on the influence of *gender,* so it is usually included as a control variable^78,79^. Gender differences are found in life satisfaction and academic cheating. It was found that girls and women are less satisfied with their lives than boys and men^80,81^, which could also be confirmed under COVID-19^82^. Male students cheated at university more often than female students^8,83^. The findings on gender differences in procrastination are varied. One researcher^84,85^. reported higher procrastination in females; Özer and Ferrari^86^ derived no significant differences.

*Academic self-efficacy*, according to Bandura, consists of a person's belief that he or she can successfully cope with difficult situations and challenges, such as exams, on his or her own. Empirical findings highlight the following relationships between study variables and self-efficacy: self-efficacy is positively associated with PBJW^87,88^ and life satisfaction^89^ and found to be negatively related to both academic dishonesty^90–92^ and procrastination^93^.

State procrastination is associated with dysfunctional time management^63,94,95^. Work tends to be completed last minute^96^. For this reason, the *distance to the next exam* and *current exam preparations* will be controlled in the study.

*Socially desirable response behavior* is a potential source of outcome bias, especially for moral themes. Paulhus^97^ describes it as the tendency of a person to give positive self-descriptions and to answer in a socially conforming way. Thus, the truthfulness of the response depends on the tendency to answer socially desirable (for a review, see Tourangeau and Yan^98^). With secured privacy and anonymity of the response, the truth content also increases^99^. Kemper and colleagues^100^ consider it important to control the influence on moral questions. Due to the morally sensitive items on academic cheating, life satisfaction, and PBJW, socially desirable responses are suspected in the study^101,102^. In this study, the approach of Kemper, Beierlein, Bensch, Kovaleva, and Rammstedt^100^ is used. Here, two facets of social desirability are distinguished: the *overstatement of positive traits (PQ*+*)* and the *understatement of negative traits (NQ−)*.

## Method

### Test persons and procedure

A total of N = 291 students from a variety of universities in Germany took part in this study. The age range in the total sample was 17–68 years with a mean age of 25.0 years (*SD* = 6.71). Overall, 21.9% of the students were male and 76.7% were female. Four students did not provide a response in this regard. A total of 28.6% of students reported writing an exam in the next 3 weeks, 29.3% in the next 3–6 weeks, and 42% later than 6 weeks. Sixty percent of students were currently studying for an exam, while 40% were not preparing for an exam.

The survey took place at the end of the 2021 summer semester. The timing was chosen due to its proximity to the exam period, where state-procrastination can be more specifically measured. The study was designed as an online survey. The sample was to be drawn from students at the university in order to be able to specifically answer the hypotheses of the study. There were no other exclusion criteria. Students were invited to take part in the online survey, which lasted approximately 20 min, through various recruitment platforms. Primarily, student teachers and psychology students of the University of Cologne were invited to join the study via the digital platform ‘Ilias’. Further, students from all over Germany were recruited via the website ‘surveycircle’. Participation was voluntary and anonymous. The questionnaire was introduced through standardized instructions that included the aim as well as information about the voluntary nature and anonymity of participation. Further, demographic data (age, gender, learning activities, distance to next exam) were collected. The measurement instruments on PBJW, GBJW, fellow student justice, lecturer justice, procrastination, cheating, life satisfaction, and self-efficacy were presented randomly. No inference can be drawn from the personal data collected. Upon successful completion of the survey, a certificate of participation equivalent to 0.5 experimental subject hours may be earned, which will receive academic credit. If students dropped out of the survey prior to completing it, a partial credit of 0.2 experimental subject hours was earned. Subject hours count as a required examination performance at the University of Cologne and are issued by the researcher of the study. Furthermore it was certified by the Psychology Examination Office of the University of Cologne. The study is conducted in accordance with the ethical guidelines of the German Psychological Society.

### Measures

Nine instruments used in the study were assigned to four content groups: internal personal factors, justice experiences, life satisfaction, and cheating. All instruments were available in German and were used as a combined online tool. For seven scales, test persons were instructed to indicate their responses on a 6-point scale ranging from *completely agree* to *completely disagree*. Academic cheating was measured with a 6-point scale ranging from 1 (*never*) to 6 (*very often*). Only for procrastination was a 5-point scale used, ranging from *never* to *always*.

#### Internal personal factors

The *Personal BJW Scale* contains 7 items of justice beliefs in their own lives ("Overall, events in my life are just"^103^). In the present study, the internal consistency for the sample was α = 0.84 (α varied in other studies between α = 0.68 and α = 0.84^3,8^). Dalbert^7^ successfully tested the factorial validity of the scale.

The *General BJW Scale*^104,105^ contains 6 items that capture the belief of an overall justice in the world ("I think basically the world is a just place."^105^). The internal consistency of the scale for the sample was α = 0.75 (α varied in other studies between α = 0.74 and α = 0.90^26^). Dalbert^7^ describes the factorial validity of the scale and Schmitt et al.^104^ describe its norm-referenced validation.

The German version of the *Aitken Procrastination State Inventory* (APSI-d) includes 22 items, which are subdivided into 3 subscales (State-Procrastination: "You have been distracted from work."; Anxiety and Uncertainty: "You have experienced feelings of panic while learning."; Aversion: "You have felt a downright hatred for learning."). Patzelt and Opitz^106^ reported internal consistencies of α = 0.82 for the Aversion subscale, α = 0.85 for the Anciety and Uncertainty subscale, and α = 0.89 for the State-Procrastination subscale. Evidence of convergent, divergent, and criterion validity is reported. In the present study, internal consistencies were α = 0.86 for the State-Procrastination subscale, α = 0.88 for the Anxiety and Uncertainty subscale, and α = 0.82 for the Aversion subscale. In the study, only the subscale State-Procrastination is considered.

The *Academic Self-Efficacy Scale*^107^ measures with seven items the students' expectation to deal effectively with study-related requirements (α = 0.77; α ranged in other studies between α = 0.79 and α = 0.84^108^; “Even though a test is very difficult, I know that I will succeed”^107^).

In a justice-related context, an increased tendency of socially desirable response behavior is likely. Using the *Social Desirability Gamma Short Scale* (KSE-G; Kemper et al.^100^), response behavior was examined. The scale consists of two subscales (Overstatement of positive qualities: PQ+, "In an argument, I always remain factual and stick to the facts."; Understatement of negative qualities: NQ−, "It has happened that I have taken advantage of someone in the past," reverse coded) with 3 items each. Acceptable reference values for reliability estimates were 0.71 for PQ+ and 0.78 for NQ−^100^. In my study, internal consistencies were: PQ+: α = 0.77; NQ−: α = 0.55. According to Kemper et al.^100^, the scale can be described as an economical, reliable, and valid measure of the gamma factor of socially desirable response behavior.

#### Justice experiences

Lecturer justice was assessed using a modified and already established version of the 10-item *Teacher Justice Scale*^109^. Items included statements about students' personal justice experiences with their lecturers (α = 0.85; α ranged from α = 0.87 to α = 0.88 in other studies^52,110^; "My lecturers generally treat me fairly"^109^). To survey fellow student justice, the adapted version of the *Classmate Justice Scale*^51^ with six items was applied (German: α = 0.84; α ranged from α = 0.82 to α = 0.87 in other studies; "My fellow students generally treat me fairly"^51^).

#### Life satisfaction

The *Trait Well-Being Inventory* by Dalbert^111^ includes a 7-item subscale on general life satisfaction. Students were asked here to rate their satisfaction with past and present life as well as future perspectives (German: α = 0.85; α ranged from α = 0.86 to α = 0.90 in other studies^17,111^; "I am satisfied with my life"^112^).

#### Academic cheating

Using 16 items from McCabe and Trevino’s^112^ *Academic Integrity Survey*, academic cheating was assessed via self-report. Students indicated by answering the items how often they engaged in academically dishonest behavior in university (German: α = 0.90; α ranged from α = 0.75 to α = 0.90 in other studies^57,90^; "copied from another student during a test or exam"). To make the instrument's statement more valid, academic cheating should be evaluated in relation to the entire study.

### Analytical strategy

The analysis of the data is performed in two steps. The correlations between the variables were performed first to identify trends in the results.

In the second step, the data were examined using mediation analyses. Due to the nonparametric data, MACRO PROCESS v4.0 was found to be suitable for analyzing mediations and was used for calculation.

The macro was developed by Hayes^113^ specifically for running various regression models of nonparametric data with an integration of mediators and covariates. Thus, it is robust to violations of the normal distribution. Model 4 of PROCESS^77^ is suitable for the mediation hypotheses established in the study. It can include up to ten mediators operating in parallel and simultaneous control of covariates. The mediation analysis was performed step by step by gradually including the control variables. In each model, direct and indirect effects were computed, as well as bootstrap confidence interval (CI) estimates for indirect effects. Following Hayes^113^, 5000 bootstrap samples are used to generate the CI. A CI is interpreted as not significant if it contains zero. The use of bootstrapping also allows mediation analyses to be examined with medium-sized samples. Chernick^114^ recommends a sample size of at least 50. Larger samples provide additional assurance of statistical results. Consequently, the study sample of N = 291 is appropriate for hypothesis testing.

### Ethical approval

All procedures performed in this study were in accordance with the ethical standards of the institutional research committee.

### Informed consent

Informed consent was obtained from all individual participants included in the study.

## Results

The results will be used to answer the following research questions (see hypotheses in chapter *present study*):

1. Are university students who believe more strongly in a just world more satisfied with their lives during COVID-19?
2. Are university students with stronger PBJW less likely to report academic cheating during COVID-19?
3. Is the assumed negative relationship between PBJW and academic cheating mediated by perceived lecturer justice?
4. Is the assumed positive relationship between PBJW and life satisfaction meditated by perceived fellow student justice?
5. Is the assumed positive relationship between PBJW and life satisfaction and the assumed negative relationship between PBJW and academic cheating meditated by state-procrastination?

### Correlational results

First, zero-order correlations between all variables were examined as part of the analytical strategy (see Table 1). From the correlation results, the hypothesized relationships can be identified. All correlations relevant to the analysis of the research hypotheses are presented. Consistent with the first hypothesis, students' PBJW correlated positively with life satisfaction and negatively with academic cheating. The more strongly students believed in a just world, the higher their life satisfaction and the less likely they were to report cheating.

**Table 1:** Bivariate correlations and descriptive statistics of study variables (N = 291 university students).

	PQ+	NQ−	Procrastination	Personal BJW	Academic cheating	Fellow student justice	Lecturer justice	Well-being	General BJW	Self-efficacy	Learning	Sex	Age	Distance to exam
PQ+	1	− 0.116	0.302**	0.405**	− 0.274**	0.128*	0.136*	0.424**	0.288**	0.442**	0.099	− 0.005	− 0.020	− 0.028
NQ−	− 0.116	1	0.137	0.068	0.146*	− 0.173**	− 0.217**	0.021	0.052	0.014	− 0.036	0.034	0.051	− 0.021
State-Procrast-ination	0.302**	0.137	1	0.437**	− 0.219**	0.208**	0.244**	0.505**	0.315**	0.519**	0.016	− 0.019	0.039	− 0.006
Personal BJW	0.405**	0.068	0.437**	1	− 0.170**	0.499**	0.419**	0.743**	0.406**	0.548**	0.013	0.079	0.124*	− 0.041
Academic cheating	− 0.274**	0.146*	− 0.219**	− 0.170**	1	− 0.308**	− 0.442**	− 0.180**	0.156*	− 0.156*	− 0.072	− 0.019	0.161*	− 0.052
Fellow student justice	0.128*	− 0.173**	0.208**	0.499**	− 0.308**	1	0.763**	0.369**	0.052	0.251**	− 0.100	0.065	− 0.019	0.005
Lecturer justice	0.136*	− 0.217**	0.244**	0.419**	− 0.442**	0.763**	1	0.347**	− 0.072	0.357**	− 0.051	0.058	− 0.068	0.002
Well-being	0.424**	0.021	0.505**	0.743**	− 0.180**	0.369**	0.347**	1	0.468**	0.607**	− 0.032	0.181**	− 0.001	− 0.051
General BJW	0.288**	0.052	0.315**	0.406**	0.156*	0.052	− 0.072	0.468**	1	0.295**	0.062	0.147*	0.048	0.034
Self-efficacy	0.442**	.014	0.519**	0.548**	− 0.156*	0.251**	0.357**	0.607**	0.295**	1	0.025	− 0.012	− 0.034	0.048
Learning	0.099	− 0.036	0.016	0.013	− 0.072	− 0.100	− 0.051	− 0.032	0.062	0.025	1	− 0.097	0.006	0.620**
Sex	− 0.005	0.034	− 0.019	0.079	− 0.019	0.065	0.058	0.181**	0.147*	− 0.012	− 0.097	1	0.004	− 0.047
Age	− 0.020	0.051	0.039	0.124*	0.161*	− 0.019	− 0.068	− 0.001	0.048	− 0.034	0.006	0.004	1	0.039
Distance to exam	− 0.028	− 0.021	− 0.006	− 0.041	− 0.052	0.005	0.002	− 0.051	0.034	0.048	0.620**	− 0.047	0.039	1

For sex, 1 = female and 2 = male; for learning, 1 = yes, 2 = no; for distance to exam, 1 = up to 3 weeks, 2 = between 3-6 weeks, 3 = later then 6 weeks; psychological variables ranged from 1 to 6 with lower values indicating stronger endorsement of the constructs, procrastination ranged from 1 to 5 with higher values indicating stronger endorsement of the constructs; BJW = belief in a just world; PQ+ = overstatement of positive qualities; NQ- = understatement of negative qualities. Correlations: point-biserial correlation between dichotomous and continuous variables; product-moment correlation between continuous variables.

In addition, there was a significant relationship between students' PBJW and procrastination, lecturer justice, and fellow student justice. The more students affirmed PBJW, the less they seemed to procrastinate and the more just they perceived the behavior of their fellow students and lectures.

Furthermore, positive correlations were shown between procrastination, lecturer justice and fellow student justice, with life satisfaction, and negative correlations with academic cheating.

Outside of the study assumptions, other correlations with control variables emerged. Life satisfaction correlated positively with GBJW, self-efficacy, and the social desirability indicator "overstatement of positive qualities" and negatively with academic cheating; male students reported higher life satisfaction. Academic cheating correlated positively with GBJW and negatively with life satisfaction, self-efficacy, and the social desirability indicator "overstatement of positive qualities"; it was more likely to occur in older students.

### Prediction of life satisfaction

The main focus of the statistical analysis was on the mediation analyses of procrastination, lecturer justice, and fellow student justice on the relationship between PBJW and life satisfaction. The analysis was conducted in three steps. In Model 1, mediation is reported without considering control variables. They were added to Model 2 (nonpsychological variables) and Model 3 (psychological variables). The main results are summarized in Fig. 1.

Model 1 showed a significant direct effect of PBJW and procrastination on life satisfaction (hypothesis 1) but no direct effect of lecturer justice (hypothesis 4) and fellow student justice. The same was found for the mediation analyses. Only procrastination mediated the direct effect of PBJW on life satisfaction. Including the non-psychological variables (gender, time to exam, studying for exam), the explained variance in Model 2 decreased slightly (hypothesis 5a). There were nonsignificant changes in the direct and indirect effects of predictors and mediators; of the control variables, only the effect of gender was significant. If the psychological variables (GBJW, self-efficacy, and the two indicators of social desirability) were additionally considered (Model 3/Table 2), the direct and indirect effects of the predictors and mediators changed slightly. The effects of gender (women) and self-efficacy were significant. The explained variance of this model was higher with *R*^2^ = 69.16% (*F*_12.161_ = 30.09, *p* < 0.000).

**Table 2:** Results of bootstrap mediation analyses for life satisfaction including control variables (N = 291 university students).

Life satisfaction				
	b (se)	t	p	CI
**Direct effects**				
Constant	− 0.92 (0.46)	− 2.01	0.05	[− 1.83; − 0.01]
Personal BJW	0.57 (0.07)	7.75	< 0.001	[0.43; 0.72]
Lecturer justice	− 0.01 (0.09)	− 0.16	0.87	[− 0.19; 0.16]
Fellow student justice	− 0.04 (0.08)	− 0.47	0.63	[− 0.19; 0.12]
Procrastination	0.23 (0.09)	2.64	0.009	[0.06; 0.40]
**Indirect effects**				
Total	0.00 (0.05)	–	–	[− 0.08; 0.09]
Lecturer justice	− 0.01 (0.05)	–	–	[− 0.10; 0.10]
Fellow student justice	− 0.02 (0.07)	–	–	[− 0.16; 0.11]
Procrastination	0.04 (0.02)	–	–	[0.01; 0.08]
**Direct effects of control variables**				
Gender	0.32 (0.11)	3.01	0.003	[0.11; 0.53]
Age	0.00 (0.01)	0.01	0.99	[− 0.01; 0.01]
Learning	− 0.16 (0.12)	− 1.30	0.19	[− 0.41; 0.08]
Distance to exam	0.10 (0.07)	1.42	0.16	[− 0.04; 0.25]
General BJW	0.09 (0.06)	1.70	0.10	[− 0.02; 0.21]
Self− efficacy	0.20 (0.08)	2.63	0.001	[0.05; 0.34]
Overstatement of positive qualities	0.06 (0.06)	1.07	0.29	[− 0.05; 0.17]
Understatement of negative qualities	0.01 (0.07)	− 0.17	0.87	[− 0.15; 0.13]

BJW = Belief in a just world; For gender, 1 = female and 2 = male. Psychological variables ranged from 1 to 6 with lower values indicating stronger endorsement of the constructs, procrastination ranged from 1 to 5 with higher values indicating stronger endorsement of the constructs. Values describe unstandardized path coefficients. *CI* = 95% confidence interval. Estimates are based on a bootstrapping procedure with 5000 bootstrap samples. Used Process (Hayes 2018) Model 4.

### Prediction of academic cheating

For the mediation analyses between procrastination, lecturer justice, and fellow student justice on the relationship between PBJW and academic cheating, the same procedure (see “Prediction of life satisfaction”) was used (Table 3). In Model 1, mediation results are presented without considering control variables. In Model 2, the non-psychological variables were added to the analysis, and in Model 3, the psychological variables were added. The main results are summarized in Fig. 2.

**Table 3:** Results of bootstrap mediation analyses for life satisfaction including control variables (N = 291 university students).

Academic cheating				
	b (se)	t	p	CI
**Direct effects**				
Constant	5.75 (0.51)	11.27	< 0.001	[4.74; 6.76]
Personal BJW	0.06 (0.08)	0.73	0.46	[− 0.10; 0.22]
Lecturer justice	− 0.43 (0.10)	− 4.33	< 0.001	[− 0.62; − 0.23]
Fellow student justice	0.04 (0.08)	0.48	0.63	[− 0.13; 0.22]
Procrastination	− 0.26 (0.09)	− 2.61	0.01	[− 0.45; − 0.06]
**Indirect effects**				
Total	− 0.22 (0.07)	–	–	[− 0.35; − 0.08]
Lecturer justice	− 0.20 (0.06)	–	–	[− 0.34; 0.10]
Fellow student justice	0.03 (0.05)	–	–	[− 0.07; 0.14]
Procrastination	− 0.04 (0.02)	–	–	[− 0.08; − 0.01]
**Direct effects of control variables**				
Gender	− 0.11 (0.12)	− 1.00	0.32	[− 0.35; 0.11]
Age	0.01 (0.07)	1.57	0.11	[− 0.003; 0.03]
Learning	− 0.07 (0.13)	0.48	0.63	[− 0.34; 0.20]
Distance to exam	− 0.04 (0.08)	− 0.52	0.61	[− 0.20; 0.11]
General BJW	0.20 (0.07)	2.98	0.003	[0.07; 0.32]
Self-efficacy	0.20 (0.08)	2.44	0.02	[0.04; 0.37]
Overstatement of positive qualities	− 0.22 (0.06)	− 3.44	0.001	[− 0.34; − 0.10]
Understatement of negative qualities	0.09 (0.08)	1.12	0.26	[− 0.07; 0.24]

BJW = Belief in a just world; For gender, 1 = female and 2 = male. Psychological variables ranged from 1 to 6 with lower values indicating stronger endorsement of the constructs, procrastination ranged from 1 to 5 with higher values indicating stronger endorsement of the constructs. Values describe unstandardized path coefficients. *CI* = 95% confidence interval. Estimates are based on a bootstrapping procedure with 5000 bootstrap samples. Used Process (Hayes 2018) Model 4.

Model 1 showed a significant direct effect of procrastination and lecturer justice on academic cheating, and no other direct effects could be obtained from the analysis (hypotheses 2/3/5b). Deducing significant indirect effects, the influence of PBJW was mediated by procrastination and lecturer justice.

After adding gender, age, time to exam, and learning for exam, the explained variance in Model 2 increased slightly; the direct and indirect effects of predictors and mediators changed. The direct effect of PBJW on cheating became significant, whereas the effect of procrastination did not. None of the control variables showed a significant effect. In the next step of the analysis, GBJW, self-efficacy, and the two indicators of social desirability were added (Model 3/Table 3). Again, modified significant direct and indirect effects were found. There was no direct significant effect of PBJW on cheating but of lecturer justice and procrastination on cheating. For the control variables, the effect of GBJW, self-efficacy, and overstatement of positive qualities as an indicator of social desirability was significant. The explained variance of this model was higher with *R*^2^ = 34.39% (*F*_12,160_ = 6.99, *p* < 0.001).

## Discussion

The aim of the study was to investigate the function of PBJW as an adaptive resource in life-critical situations and to support its stability based on the study results. Thus, the focus was on whether the results of Münscher et al.'s^8^ study could be replicated during the COVID-19 pandemic. In addition, since digital teaching required under COVID-19 changed the communication structure and social interaction at the university with lecturers and fellow students, the significance of justice experiences and university work behavior should be tested.

The expected associations between PBJW and life satisfaction and academic cheating and mediation effects on university students were able to partially replicate recent findings from school and university^8,31,41,79,115,116^ and supported most of the study hypotheses. Justice experiences with fellow students were not significant in this study compared to studies outside of COVID-19 pandemic. Below is a practical discussion of all study hypotheses.

Consistent with the first hypothesis, PBJW had a significant positive effect on life satisfaction. No significant negative effects on academic cheating were shown, in contrast to the second hypothesis. Often, an effect of PBJW on the outcome variable can be mediated indirectly; to check this, a mediation analysis follows. This approach is supported by Warner^117^ and Hayes and Rockwood^118^. A significant direct effect of X on Y is not considered a prerequisite for further mediation analyses. Evidence of the direct effect of PBJW on academic cheating is provided by analysis Model 2, where a significant effect of PBJW on students' cheating behavior could be shown after adding the non-psychological control variables. Finally, the more university students believed in a personally just world, the more satisfied they were with their lives, and the less frequently they reported cheating in their studies.

The study results fit into the findings of justice research and support the assumption that PBJW functions as an internal resource under both unstressed and stressed situational conditions^20,51,74,110,119–121^. Due to the diverse research on PBJW and its protective function in everyday life and during extraordinary life events in children, adolescents, and adults, a generalization of the effects of PBJW can also be supported by the findings of this study. Dalbert^5^ defined BJW as an experience-based disposition. In this study, PBJW protected university students' life satisfaction and strengthened their justice-related university work behaviors. The current evidence during the COVID-19 pandemic^43^ and results from other studies^3,30,122,123^ support the robustness of belief in a just world.

It is suggested that the adaptive functions of the PBJW commit students with a strong PBJW to behave more justly toward others, cognitively reinterpret unjust and life-critical experiences at university, and principally trust others more. The present results do not allow for causal inferences, so the assumptions should be subjected to further investigation.

In the next section, the mediation analyses (hypotheses 3/4/5) are discussed in terms of justice experiences and procrastination. Justice experiences are obtained in direct social contact^124^. The assimilation function is particularly important in the case of injustice experiences for the maintenance of the PBJW^17^. Thus, in the university context, justice experiences with fellow students and lecturers were examined. Interventions against COVID-19 required universities to adapt teaching^4,55^. At the University of Cologne, digital synchronous and asynchronous teaching was offered, which led to contact reduction among students and with lecturers.

As mentioned above, strong justice believers basically notice the behavior of others as more just. The perception changes the person's own experience and behavior and commits the person to his or her own just behavior^8,17^. From this, justice experiences seem to take on a mediator role. Due to contact reduction interventions, the influence of justice experiences might be lower than in studies under face-to-face teaching. In the third and fourth hypotheses, it is hypothesized that justice experiences of university students would mediate, at least in part, the relationships between their PBJW and life satisfaction and academic cheating. During digital teaching, the established rules and norms of lecturers and their interactions with students provide basic information on their justice-related behaviors^48,125–128^. Nevertheless, during digital teaching, face-to-face interaction as well as nonverbal communication, which is crucial for communicative understanding, is missing. The interpretation of others' justice-related behavior depends, among other things, on the individual learning history^129^. The prerequisite for this is social contact, which is strongly curbed in the case of digital teaching.

The necessary condition for this is social contact, which is severely limited in the case of digital teaching. The extent to which university justice experiences serve as a value orientation in this situation^130,131^ and contribute to students' self-esteem^132,133^ is difficult to assess.

The present study results suggest no indirect effect of justice experiences on life satisfaction and support the hypothesis that perceptions of just lecturer behavior mediate the effect of PBJW on academic cheating. University students with a strong belief in a just world perceived their fellow students and lecturers to be more just than students who were less convinced of a just world. Compared to the study of Münscher et al.^8^, in this study, perceived justice of fellow students is less important for students' life satisfaction during the COVID-19 pandemic. The reason for this could be the lower contact between students. A replication of the mediation effects of existing studies on PBJW, justice experiences, and life satisfaction, such as those shown in Donat et al.^79^ or Münscher et al.^8^, could not be realized here. Previous studies describe a situation-specific effect of justice experiences on life satisfaction, so it is possible that under COVID-19 a reliance on external private resources, such as close friends and family, are more important for life satisfaction^78,134^. This domain specificity of justice experiences was discussed by Dalbert and Stoeber^52^. Therefore, the third hypothesis can only be partially supported. In contrast, the results are consistent with the fourth hypothesis. Students with a strong PBJW perceived their lecturers' behavior as more just and felt committed to more just examination behavior.

One possible reason would be the high transparency of established rules and norms, as these provide a guiding framework for the required student behavior in digital teaching and digital exams. As many researchers^48,125^ describe, the transparency of behavioral guidelines in exams is crucial for exam behavior. High procedural and interpersonal fairness also promotes students' motivation to behave conform to the rules^6,79,135^.

In addition, procrastination has been introduced as another mediator. First evidence for its mediating effect on cheating or life satisfaction was provided in a preliminary study^15^. According to the results, students with strong PBJW procrastinated less, were more satisfied with their lives, and cheated less. The clinical disorder of procrastination is defined by, among other things, a deficit in self-regulation as well as a motivational deficit^58,59,63^. Several studies on PBJW indicate long-term goal pursuit^70^, better emotion regulation^136^, and higher work motivation^31^ compared to individuals with low PBJW. Thus, the adaptive functions of PBJW could promote academic work and provide better work attitudes^120^. In this study, state procrastination was measured based on the situational stress of COVID-19, which is not understood as a habitual disposition but as situationally conditioned. Necessary academic activities may be postponed by students for anxiety about being evaluated by others, among other reasons^95,137^. It is possible that PBJW, with its trust function and motivational function, is protective and acts as a resource here against evaluative anxiety. Confidence in others' just treatment toward oneself and commitment to follow university rules and norms could reduce students' situational procrastination^138^. Adaptive work behavior subsequently has a positive impact on life satisfaction and honest academic work. The current finding is consistent with the theoretical basis of the justice motive and supports the assumption of the mediator function of procrastination. The results can highlight procrastination as an indicator of motive function and support the assumptions of the fifth hypothesis of the study.

In addition to the main results discussed, a look should be taken at the influence of the control variables. Only significant effects on outcome variables are discussed. The direct and indirect effects of PBJW on life satisfaction and academic cheating should not change even when the control variables of gender, age, exam preparation, time to next exam, academic self-efficacy, social desirability, and GBJW were added. The control variables provided no significant change in the study results. Regarding gender, male students reported being more satisfied with their lives than female students, as also observed by Bergold et al.^80^.

For both life satisfaction and cheating, academic self-efficacy proved to be an important control variable^8,88,89,93^. Students with high self-efficacy reported being more satisfied with their lives and cheating less. This finding can be supported by recent research^91^ and highlights self-efficacy as another important resource in life-critical situations. Particularly in digital instruction, self-efficacy seems to play a crucial role due to the required independent work. In future studies, it should be used as a complementary mediator in justice research. A first approach to this was examined by Kiral Ucar et al.^41^, who pointed out self-efficacy as a mediator in the relationship between PBJW and life satisfaction.

In addition, students' response behavior was controlled for social desirability. Due to the sensitive moral items in the inventory, controlling for response behavior was essential. Both justice-related items and statements about dishonest academic work may increase socially desirable response behavior. Sepùleveda et al. found no relationship between social desirability and reported life satisfaction, which can be replicated in the present study. Vesely and Klöckner^139^ reported that social desirability is only a subtle influencing factor that can lead to biases in response behavior under very specific conditions. From the study results, it can be inferred that students who overstate their positive qualities seem to be less likely to cheat. A finding on the dimension "understatement of negative qualities" cannot be deduced from the results. This result is in contrast to previous research^140,141^. To provide more clearness in this regard, future studies on PBJW and rule-breaking behavior should control for the variable “social desirability”.

To indicate the relevance of the predictor power of PBJW, GBJW was obtained comparatively. The results are interesting and fit with the study findings of Schindler et al.^142^ regarding the relationship between dishonest behavior and justice beliefs. Hafer and Sutton^32^ described the association of GBJW with socially inappropriate behavior in their review. The results of the present study also indicate a relationship between GBJW and cheating. Adoric^34^ addressed the stability of motive dimensions in their study. They found that PBJW is more vulnerable to experiences of injustice. A decrease in personal justice beliefs could not be confirmed in the current study, so PBJW can also be defined as a robust disposition. GBJW was also a significant predictor in this study and did not promote adaptive functional behavior and experience. As expected, PBJW was a more important predictor in this study.

In conclusion, from the present results and existing findings, the PBJW with its adaptive functions seems to be a significant resource even in life-critical events, such as COVID-19 pandemic. It serves as a protective measure for maintaining life satisfaction and functional academic work during the COVID-19 pandemic. Thus, the results complement the given empirics^3,18,41,51^. Through the findings of the present study, the validity of the adaptive function of PBJW can be assumed and its stability in life-critical situations can be highlighted. The situation-specific effect of justice experiences discussed by Dalbert and Stoeber^52^, among others, can be used here as an explanation for their partial lack of significance. Under COVID-19, the private social environment most likely mediates the relationship between well-being and justice beliefs. The importance of situational justice experiences for social behavior may be supported by the effect of lecturer justice on students' cheating. Outside justice experiences, other mediators between PBJW and life satisfaction as well as students' social behavior would be interesting to investigate. This study considered students' work behaviors, which were significant for both life satisfaction and academic cheating during the COVID-19 pandemic. However, the generalizability of PBJW's effects on life satisfaction and academic cheating mediated by lecturer justice and procrastination requires further replication. In addition, follow-up studies on the differential effects of justice experiences and self-reported work behavior as a result of PBJW's adaptive function among university students would be valuable.

### Practical implications

The study results provide incremental research-practice validity. On the one hand, PBJW could be identified as a stable disposition that represents a resource in life-critical situations, and on the other hand, procrastination could be explored in its significance as a mediator. Furthermore, practical derivations for the university context can be drawn from the study results. According to the adaptive function of the PBJW, experiences of justice and one's own fair behavior are important for well-being and academic cheating. Fair treatment by lecturers at the university motivates students to accept and follow rules and norms. In particular, the design of teaching also supports a sense of justice, such as the transparency of examination conditions and the right to have a say. According to Sanches et al.^130^, acceptance and rule-following behavior can be transferred to institutions such as the police, court, and judiciary. Educational tasks are thus not only provided in the school but also in the university.

### Limitations and prospects

Several limitations of the study should be noted here. The data of the study were collected cross-sectionally due to the pandemic situation. Thus, no conclusions about cause and effect can be concluded. It would be interesting for future research to embed a longitudinal design, as this would allow a statement to be made about the causality of the results.

Some scales were adapted to the university student sample so that slight changes in understanding could have occurred here. No irregularities are detectable in the statistical analysis. All scales except the subscale “understatement of negative qualities” showed satisfactory reliability coefficients. The lack of test quality of the subscale could be explained by the small number of items.

Generalization of the results is possible to a limited extent. Weaknesses are the disproportionality of genders and study programs in the sample. It was possible to recruit far more women than men and students from the study programs in education and psychology. Students reported from their subjective perspective on their justice beliefs, their justice experiences, their university work behaviors, and their life satisfaction. All of these topics are susceptible to socially desirable response behavior, which was controlled for this reason. Basically, the goal was to assess students' life satisfaction. Here, the cognitive component of general life satisfaction was considered, as it is of greater interest during the COVID-19 pandemic than specific university well-being. Nevertheless, the domain-specific investigation of well-being would be worth considering in future studies.

To avoid focusing students' awareness on the stresses of the pandemic, no questions were asked here. Thus, a comparison of students experiencing high or low levels of stress was not possible. Further research in the area of procrastination also seems useful. In this study, state procrastination was recorded. Complementary, analysis of trait-procrastination and self-regulatory processes would also be integrated in further research. In the current study, only some mediators and control factors were tested. In addition, consideration of other influencing factors, such as personality factors and motivational constructs^88^, on life satisfaction and academic cheating would also be important.

## Conclusion

The findings of this study strengthened existing findings from school and university contexts and supported the stability and robustness of the adaptive effect of PBJW on life satisfaction and justice-compliant behavior.

PBJW represents a protective factor not only in everyday life but also in life-critical situations, which has a positive effect on students' overall life satisfaction and honest behavior in examination situations. The adaptive functions of the PBJW can change the perception of one's own behavior and the behavior of others. Evidence for this can be derived from the study results. Students with high PBJW perceived both the behavior of their fellow students and lecturer to be more justly and at the same time showed less procrastination. However, the influence of justice experiences appears to be situation-dependent, such that only lecturer justice mediated the effect of PBJW on students’ academic cheating. A critical mediator of life satisfaction and cheating was reported procrastination behavior. The pandemic-altered learning environment may also lead to a decrease in the importance of university justice experiences and an increase in the importance of students' own work behaviors. The consideration of distributive, procedural, and interpersonal justice^6^ also seems to be an important orienting factor for students, especially in the context of digital teaching and digital examinations.

Following the results, the potential of justice experiences should be used in digital teaching and their perception should be promoted. The feeling of belonging, inclusion, and appreciation among students in digital teaching should be conveyed by lecturers and fellow students to increase their well-being and engage them in compliant behavior.

## Supplementary Information

Supplementary Information.

## Data Availability

All data generated or analysed during this study are included in this published article [and its supplementary information files].
