# Supplementary material for: The relationship between just world belief and wellbeing, cheating behaviors, and academic work behaviors during COVID 19 among university students
Source: Sci Rep. 2022 Aug 22;12:14328. doi: 10.1038/s41598-022-18045-7 (PMC9395426; doi:10.1038/s41598-022-18045-7)
Supplement: Supplementary file 1 — Supplementary Information. [file 41598_2022_18045_MOESM1_ESM.pdf]

Data_Justice beliefs during COVID-19? beliefs during COVID-19?

Alter	Geschlecht	Studiengang	timetoexam	examlearning	AGWG1	AGWG2	AGWG3	AGWG4	AGWG5	AGWG6	DG1	DG2	DG3	DG4	DG6	DG7	DG8	DG9	DG10	DG2x	DG4x	DG7x	DG6x	DG8x	DG10x	KG1	KG2	KG3	KG4	KG5	KG1x	KG4x	KG5x	LZ1	LZ2	LZ3	LZ4
					5	5	5	5	5	5	5	5	5	5	5	5	5	5	5	2	2	2	2	2	2	5	5	5	5	5	2	2	2	5	5	5	5
			1	1	6	6	6	6	6	6	6	6	6	6	6	6	6	6	6	1	1	1	1	1	1	6	6	6	6	6	1	1	1	6	6	6	6
			1	2	6	6	6	2	6	6	6	6	6	6	6	6	6	6	6	1	1	1	1	1	1	6	6	6	6	6	1	1	1	6	6	6	6
23	1	Bachelor	2	2	6	2	6	3	4	2	1	5	2	3	5	5	4	2	3	2	4	2	2	3	4	4	2	2	5	5	3	2	2	2	2	2	3
20	1	Psychologie	3	2	6	1	6	4	2	3	2	6	2	6	5	6	5	2	6	1	1	1	2	2	1	6	1	1	6	6	1	1	1	3	1	2	2
20	1	Psychologie	3	2	6	5	6	5	4	4	1	6	1	6	6	6	6	2	6	1	1	1	1	1	1	6	1	1	6	6	1	1	1	2	2	2	4
21	1	Psychologie	3	2	4	2	6	5	1	4	5	1	4	5	5	5	3	4	2	6	2	2	2	4	5	5	5	5	5	3	2	2	4	5	3	4	2
20	1	Psychologie	3	1	4	2	4	2	2	3	1	6	2	6	5	5	5	1	6	1	1	2	2	2	1	4	3	3	6	5	3	1	2	2	2	2	3
27	1	Psychologie	1	1	6	3	5	4	4	4	2	5	2	4	4	5	5	2	4	2	3	2	3	2	3	5	2	4	5	5	2	2	2	3	4	3	5
24	2	Psychologie	2	1	5	4	4	4	4	3	5	3	3	4	4	4	4	3	4	4	3	3	3	3	3	4	4	3	4	4	3	3	3	4	2	2	3
30	2	Bachelor Lehramt	3	1	6	5	6	6	5	6	4	3	4	3	3	3	3	2	1	4	4	4	4	4	6	4	3	3	3	4	3	4	3	4	5	3	5
19	1	Grundschullehramt	2	2	5	4	4	2	2	2	1	6	1	6	6	6	6	1	5	1	1	1	1	1	2	6	1	1	6	6	1	1	1	1	1	2	2
40	1	Lehramt Grundschule	3	1	4	2	4	2	2	2	2	5	2	3	5	5	5	2	3	2	4	2	2	2	4	5	2	2	5	5	2	2	2	2	2	2	2
22	1	Lehramt	3	1	4	3	4	4	3	4	2	4	2	3	5	5	4	3	3	3	4	2	2	3	4	4	2	3	4	5	3	3	2	4	4	4	4
20	1	Physik	2	1	2	6	1	1	1	1	1	5	1	6	4	5	3	1	1	2	1	2	3	4	6	3	1	1	4	3	4	3	4	2	5	2	5
19	1	Lehramt an Grundschulen	2	1	6	3	6	5	4	4	1	5	1	6	6	6	6	1	5	2	1	1	1	1	2	6	2	3	6	5	1	1	2	1	1	1	2
18	1	Lehramt an Grundschulen	3	2	5	2	4	2	3	3	2	5	2	4	5	5	5	2	4	2	3	2	2	2	3	4	2	2	5	5	3	2	2	2	2	2	3
21	1	Lehramt für Haupt real und Gesamtschulen	2	1	5	2	5	2	4	3	2	4	2	5	5	4	4	2	6	3	2	3	2	3	1	3	3	3	5	4	4	2	3	1	2	2	2
19	1	Lehramt an Grundschulen	2	2	4	2	2	4	2	3	2	5	2	4	5	5	5	2	5	2	3	2	2	2	2	5	2	2	4	5	2	3	2	2	2	2	3
20	1	Grundschullehramt (B.A.)	2	2	4	3	4	3	2	3	1	5	2	5	5	6	5	2	4	2	2	1	2	2	3	6	1	2	6	6	1	1	1	2	2	2	3
20	1	Lehramt für Gym/Ge (Mathe, Bio)	3	1	5	3	6	4	2	4	2	5	2	5	5	4	5	4	3	2	2	3	2	2	4	5	2	2	4	5	2	3	2	4	4	4	6
20	1	Grundschullehramt	2	2	6	3	5	4	2	4	1	5	1	6	6	6	6	1	4	2	1	1	1	1	3	6	2	1	6	6	1	1	1	1	1	1	1
23	1	Lehrarmt	3	1	6	2	2	1	2	1	2	5	2	5	5	5	5	2	5	2	2	2	2	2	2	5	2	2	2	5	2	5	2	2	2	3	3
21	1	Lehramt an Gymnasien und Gesamtschulen	2	1	6	2	4	3	4	5	2	5	3	5	5	5	3	2	3	2	2	2	2	4	4	6	2	3	6	6	1	1	1	1	2	2	1
19	2	Lehramt HRS-G	3	2	3	4	4	5	4	4	2	5	2	5	6	6	5	2	3	2	2	1	1	2	4	5	2	2	5	5	2	2	2	1	2	2	3
33	2	Lehramt	3	2	5	2	4	5	2	4	2	6	2	5	6	5	5	2	5	1	2	2	1	2	2	5	2	2	5	5	2	2	2	2	2	2	2
19	1	Grundschullehramt	3	1	6	5	6	5	4	6	2	5	2	5	5	6	5	2	5	2	2	1	2	2	2	5	2	2	5	5	2	2	2	2	2	2	2
19	2	Psychologie	3	2	5	3	5	6	2	2	2	5	2	3	5	5	4	1	3	2	4	2	2	3	4	4	3	2	3	3	3	4	4	1	2	2	3
19	1	Geschichte und Mathematik auf Lehramt	3	2	5	2	5	2	4	4	2	5	3	4	5	5	5	3	5	2	3	2	2	2	2	4	2	3	5	5	3	2	2	3	2	2	4
23	2	Wirtschaftswissenschaften und Englisch, Bachelor Lehramt BK	2	1	5	4	4	2	2	2	2	5	1	4	5	6	5	1	2	2	3	1	2	2	5	6	1	2	6	6	1	1	1	1	1	1	1
23	2	Psychologie	2	1	6	6	6	6	5	5	2	6	2	5	6	6	6	2	6	1	2	1	1	1	1	5	2	2	6	5	2	1	2	4	4	4	1
21	2	Psychologie	3	2	6	5	6	5	5	5	2	4	2	3	4	4	4	3	4	3	4	3	3	3	3	2	4	4	2	3	5	5	4	5	5	5	5
40	1	Lehramt an Grundschulen	2	1	6	6	6	2	2	1	2	5	2	6	5	5	5	1	6	2	1	2	2	2	1	5	2	3	6	6	2	1	1	1	4	3	5
27	2	Lehramt Haupt, Real Gesamtschule	2	1	6	5	6	6	6	6	1	5	2	5	5	5	5	2	3	2	2	2	2	2	4	3	2	2	5	5	4	2	2	2	3	3	3
20	1	Englisch, Geschichte BA Lehramt (Gym/Ge)	2	1	6	5	5	5	3	5	2	6	2	5	6	6	6	2	4	1	2	1	1	1	3	6	2	2	5	5	1	2	2	2	2	2	2
21	1	Lehramt	3	1	5	3	4	2	2	3	1	5	2	3	5	5	6	3	3	2	4	2	2	1	4	4	2	2	6	6	3	1	1	2	2	2	2
20	1	Grundschullehramt	3	2	5	3	4	4	3	3	1	6	1	5	6	6	6	2	6	1	2	1	1	1	1	6	1	1	6	6	1	1	1	3	3	3	3
23	1	Lehramt Grundschule	2	2	5	5	4	5	4	5	2	4	3	5	4	4	4	2	5	3	2	3	3	3	2	5	2	5	4	5	2	3	2	3	4	4	5
18	2	Lehramt auf haupt real gesamt sekundarschulen	3	1	5	1	3	1	2	3	3	2	3	3	3	3	3	2	3	5	4	4	4	4	4	2	3	4	4	3	5	3	4	2	3	3	5
20	1	Lehramt	2	1	6	4	3	4	4	3	2	4	2	4	4	4	4	3	3	3	3	3	3	3	4	4	2	1	4	4	3	3	3	2	4	3	4
23	1	Lehramt am Berufskolleg	2	1	5	4	5	4	2	3	3	5	5	6	5	5	4	2	5	2	1	2	2	3	2	5	2	3	6	5	2	1	2	3	2	3	4
26	2	Physik, Philosophie und Deutsch für das Lehramt an Haupt-, Real-, Sekundar-, und Gesamtschulen.	3	1	5	5	6	4	4	4	2	4	3	4	4	4	4	2	4	3	3	3	3	3	3	3	4	3	3	3	4	4	4	3	3	3	2
28	2	Geschichte und Sonderpädagogik auf Lehramt	3	1	4	2	4	5	3	3	2	4	2	4	4	4	5	3	3	3	3	3	3	2	4	5	2	2	4	4	2	3	3	4	4	4	4
21	1	B.A. Lehramt Gym/Ge	3	1	4	2	4	2	3	2	2	4	2	4	4	5	4	2	3	3	3	2	3	3	4	6	1	2	5	6	1	2	1	2	2	2	2
28	1	Lehramt an Grundschulen	2	1	6	3	5	3	4	4	1	6	1	5	6	6	6	1	6	1	2	1	1	1	1	5	2	2	6	5	2	1	2	2	2	3	2
26	1	Lehramt	3	2	5	5	5	6	2	4	2	5	2	4	5	5	5	3	3	2	3	2	2	2	4	5	2	2	5	5	2	2	2	2	2	1	3
20	1	Lehramt Hrgs	3	2	6	2	3	1	4	2	2	4	2	4	4	5	6	3	5	3	3	2	3	1	2	4	2	2	6	5	3	1	2	4	3	2	4
20	1	BA Lehramt HRSGe	3	1	5	3	4	5	5	5	2	5	3	2	5	5	5	3	3	2	5	2	2	2	4	4	2	3	2	3	3	5	4	5	6	5	6
20	1	Lehramt Haupt-,Real- und Gesamtschule	3	2	4	3	4	3	1	1	1	5	2	5	5	5	5	3	6	2	2	2	2	2	1	4	2	2	6	5	3	1	2	2	1	2	2
37	1	Lehramt HRGe	2	1	6	4	4	4	2	3	2	6	2	4	5	6	4	2	5	1	3	1	2	3	2	4	2	2	5	5	3	2	2	2	2	2	3
28	2	Psychologie	2	1	6	4	3	6	5	6	1	6	2	5	6	6	6	1	6	1	2	1	1	1	1	5	2	3	6	6	2	1	1	1	1	4	2
20	1	Lehramt HRSG	2	1	3	2	4	3	3	2	3	4	3	3	3	3	3	3	3	3	4	4	4	4	4	4	3	3	4	3	3	3	4	3	3	3	4
28	1	Bachelor	3	2	6	4	5	3	3	4	2	4	3	4	4	4	5	2	4	3	3	3	3	2	3	5	2	3	5	5	2	2	2	3	2	4	4
23	1	Lehramt Gym/Ge	2	2	5	4	4	4	2	4	3	5	2	5	4	4	3	2	4	2	2	3	3	4	3	3	3	2	3	4	4	4	3	4	2	2	3
25	1	Lehramt HRSG	2	1	6	3	5	5	3	5	2	6	2	4	6	6	6	1	6	1	3	1	1	1	1	5	2	2	5	6	2	2	1	2	1	2	2
22	1	Erziehungswissenschaft	2	1	4	2	3	4	3	3	2	5	2	5	5	5	4	2	5	2	2	2	2	3	2	5	2	2	4	5	2	3	2	2	2	2	3
23	3	Englisch Philosophie Lehramt GyGe	3	1	6	4	4	4	4	4	2	4	3	4	4	4	4	2	3	3	3	3	3	3	4	5	2	3	4	4	2	3	3	2	3	2	4
21	1	Psychologie	3	1	5	5	5	5	3	4	1	6	1	5	6	6	6	1	6	1	2	1	1	1	1	6	1	1	6	6	1	1	1	2	1	1	2
21	1	Psychologie	3	2	4	2	4	4	1	2	1	6	6	6	6	6	6	1	3	1	1	1	1	1	4	5	2	1	5	6	2	2	1	2	2	1	2
21	1	Psychologie	3	1	5	5	6	5	4	6	2	5	2	6	6	5	5	2	2	2	1	2	1	2	5	4	3	4	3	3	3	4	4	2	5	3	5
26	1	Psychologie	3	2	4	5	4	4	4	4	2	4	3	2	5	5	3	3	3	3	5	2	2	4	4	5	2	2	5	5	2	2	2	2	1	1	2
26	1	1-Fach BA Erziehungswissenschaft		1	6	4	5	5	4	6	2	5	2	6	5	5	5	2	4	2	1	2	2	2	3	6	1	1	5	5	1	2	2	3	2	2	3
24	2	Psychologie	3	2	6	6	6	6	5	6	1	6	5	6	6	6	6	3	5	1	1	1	1	1	2	5	2	2	5	6	2	2	1	2	3	4	6
19	1	Grundschullehramt	3	2	5	5	5	4	3	6	2	5	2	4	5	5	4	2	4	2	3	2	2	3	3	5	2	2	5	5	2	2	2	2	2	2	2
21	1	Psychologie	3	2	4	2	5	5	3	4	1	6	1	6	6	6	6	2	5	1	1	1	1	1	2	6	2	2	6	6	1	1	1	2	2	2	3
32	1	Psychologie	2	1	6	3	6	4	3	6	2	4	3	6	5	5	5	1	6	3	1	2	2	2	1	2	3	2	3	3	5	4	4	2	3	3	3
68	2	Gasthörer	3	2	4	3	2	4	3	4	2	6	2	3	6	6	6	2	4	1	4	1	1	1	3	6	1	2	5	6	1	2	1	3	1	2	2
22	2	Psychologie	2	1	5	3	3	5	5	5	1	6	2	6	6	6	6	1	6	1	1	1	1	1	1	6	2	1	6	6	1	1	1	2	3	2	4
42	1	Erziehungswissenschaft	2	1	6	4	6	3	4	4	4	3	4	3	3	3	3	4	5	4	4	4	4	4	2	3	5	4	3	3	4	4	4	2	2	1	2
20	1	Psychologie B.Sc.	3	1	4	4	4	5	4	5	2	4	2	4	5	5	4	2	4	3	3	2	2	3	3	3	3	4	3	3	4	4	4	3	2	4	5
21	1	Psychologie	3	1	5	3	4	4	2	3	2	4	2	3	4	4	4	2	4	3	4	3	3	3	3	4	1	2	4	4	3	3	3	2	2	1	3
21	1	Psychologie	2	2	3	5	5	5	3	6	4	3	3	3	2	4	4	2	2	4	4	3	5	3	5	1	1	3	3	5	6	4	2	2	5	3	4
19	1	Bachelor of Arts	3	1	6	4	4	3	3	4	4	3	4	4	4	3	4	3	4	4	3	4	3	3	3	5	2	2	5	6	2	2	1	3	4	3	5
21	1	Psychologie	2	1	3	1	4	4	2	3	1	3	4	3	4	4	4	1	3	4	4	3	3	3	4	2	3	3	3	3	5	4	4	1	2	1	2
32	1	Erziehungswissenschaft	2	1	3	3	3	3	3	3	3	3	3	3	3	3	3	3	3	4	4	4	4	4	4	3	3	3	3	3	4	4	4	3	3	3	3
26	1	Psychologie	2	1	4	3	2	5	2	5	2	6	2	6	6	6	6	2	6	1	1	1	1	1	1	5	2	2	6	6	2	1	1	2	3	2	3
33	1	B.A. LA GyGe	3	2	6	4	3	4	3	2	2	6	2	4	6	6	5	2	4	1	3	1	1	2	3	6	1	3	6	6	1	1	1	2	2	2	3
29	2	Psychologie	2	1	4	4	4	5	4	5	1	6	2	6	6	6	6	2	6	1	1	1	1	1	1	6	1	2	3	6	1	4	1	3	3	4	4
25	1	Psychologie	3	2	6	5	6	5	2	5	2	5	3	3	5	5	4	4	3	2	4	2	2	3	4	5	2	5	5	5	2	2	2	3	3	3	5
43	1	Psychologie	2	1	6	5	6	5	2	5	2	5	2	5	6	5	5	2	5	2	2	2	1	2	2	4	3	2	4	4	3	3	3	1	2	3	2
20	1	Psychologie	3	2	4	3	3	3	3	2	1	6	1	4	5	6	6	1	6	1	3	1	2	1	1	4	2	2	6	5	3	1	2	2	3	2	2
30	1	Psychologie Bachelor	2	1	6	4	1	4	4	5	1	6	5	4	5	5	5	2	4	1	3	2	2	2	3	6	1	2	5	6	1	2	1	2	2	2	2
28	2	Psychologie	1	1	1	6	1	5	4	5	2	3	3	1	3	2	2	4	2	4	6	5	4	5	5	3	5	4	2	3	4	5	4	1	1	1	2
38	1	psychologie	3	2	4	4	4	6	3	5	1	6	2	6	6	5	5	1	5	1	1	2	1	2	2	5	2	1	4	5	2	3	2	2	2	2	2
19	1	Grundschullehramt	3	2	5	4	4	5	3	4	1	5	1	6	5	6	5	2	5	2	1	1	2	2	2	6	1	1	5	6	1	2	1	2	2	1	2
27	2	psych	1	2	6	6	6	6	6	6	6	6	6	6	6	6	6	6	6	1	1	1	1	1	1	6	6	6	6	6	1	1	1	6	6	6	6
20	1	Psychologie	2	1	4	3	4	4	1	3	2	6	2	5	6	6	6	2	5	1	2	1	1	1	2	5	2	3	5	6	2	2	1	2	1	2	2
23	1	B. Sc. Psychologie	1	1	6	4	5	6	3	6	2	5	2	3	5	4	5	3	6	2	4	3	2	2	1	6	1	1	6	6	1	1	1	3	3	3	4
20	1	Lehramt	2	1	3	3	4	4	3	3	2	5	2	4	5	6	5	3	3	2	3	1	2	2	4	6	2	2	6	5	1	1	2	4	4	3	5
21	1	Psychologie	2	1	4	3	5	3	2	4	2	6	2	6	6	6	5	2	6	1	1	1	1	2	1	5	2	2	6	6	2	1	1	2	2	2	2
22	1	Psychologie	1	1	2	1	1	1	1	2	2	1	2	2	1	1	2	3	1	6	5	6	6	5	6	1	2	1	1	1	6	6	6	2	2	1	2
36	1	Erziehungswissenschaft	2	1	4	4	6	5	4	3	2	4	2	4	5	6	5	2	6	3	3	1	2	2	1	4	2	2	5	5	3	2	2	2	2	2	2
26	1	Lehramt	2	1																																	
21	1	Bachelor Lehramt	3	2																																	
23	1	Lehramt Haupt-, Real-, Gesamtschulen	2	1																																	
22	1	Lehramt Gym/Ge	3	2																																	
20	1	Lehramt GymGe	3	2																																	
21	3	Psychologie	2	1	4	3	4	4	2	4	2	5	3	4	5	5	4	2	5	2	3	2	2	3	2	5	2	5	5	5	2	2	2	2	3	4	3
25	2	BaLaGymGe	2	1																																	
22	1	Bachelor	2	2																																	
20	1	Lehramt	2	1																																	
27	1	Master Psychologie anwendungsorientiert Uni Köln	2	1	5	5	5	4	3	4	1	6	1	5	6	6	6	2	5	1	2	1	1	1	2	6	1	1	6	6	1	1	1	3	1	2	1
24	2	B.A. Lehramt Deutsch und Geschichte HRSG	2	1																																	
19	2	Chemie	1	1																																	
22	2	Lehramt Bio und Chemie für GYmnasium und Gesamtschule	2	1																																	
19	1	Lehramt	3	1																																	
34	1	Psychologie	3	2	6	4	5	6	5	5	2	5	2	4	5	5	5	2	5	2	3	2	2	2	2	2	5	5	2	2	5	5	5	2	2	2	3
19	1	Grundschullehramt	3	1																																	
22	2	Psychologie B. Sc.	1	1	5	5	5	4	4	4	2	6	1	5	6	6	4	2	6	1	2	1	1	3	1	6	1	1	6	6	1	1	1	1	2	1	2
28	2	Psychologie	2	1																																	
27	1	Psychologie	1	1																																	
28	1	Psychologie	2	1	4	3	5	2	2	3	2	6	2	5	5	5	6	2	5	1	2	2	2	1	2	4	3	3	5	5	3	2	2	1	2	3	3
20	1	Lehramt Grundschule	2	2																																	
35	1	0	3	2	6	6	1	6	6	1	6	1	6	1	1	1	1	6	1	6	6	6	6	6	6	1	6	6	1	1	6	6	6	6	6	6	1
21	1	Soziologe	1	2	6	5	6	6	5	6	1	6	6	5	6	6	6	2	3	1	2	1	1	1	4	6	1	1	6	6	1	1	1	5	2	3	4
22	1	Psychologie	2	1	4	3	3	3	3	2	3	6	4	4	6	6	5	1	2	1	3	1	1	2	5	5	3	2	3	6	2	4	1	3	1	1	6
20	2	Medienproduktion und Medientechnik	1	1	5	4	4	3	4	4	2	5	3	4	5	5	4	4	4	2	3	2	2	3	3	5	2	3	5	5	2	2	2	3	3	4	5
43	1	Psychologie	2	1																																	
26	2	Psychologie	2	1	4	4	3	4	3	4	3	3	3	5	3	4	4	3	5	4	2	3	4	3	2	3	2	3	5	4	4	2	3	2	2	2	2
22	2	B. A. Geschichte und Sozialwissenschaft	1	2	4	3	4	6	3	4	1	6	1	6	6	6	6	1	4	1	1	1	1	1	3	5	1	1	6	6	2	1	1	1	1	1	2
24	2	Bildungswissenschaft	1	1	4	2	5	2	3	2	1	5	2	3	5	5	5	1	4	2	4	2	2	2	3	4	2	3	5	5	3	2	2	2	2	3	3
23	1	Betriebswirtschaft & Wirtschaftspsychologie	2	1	4	3	2	2	1	2	2	6	3	6	6	6	6	2	6	1	1	1	1	1	1	5	2	3	6	6	2	1	1	2	2	2	3
25	1	Betriebswirtschaft & Wirtschaftspsychologie	1	1	6	4	3	3	5	5	2	5	2	6	5	6	6	3	5	2	1	1	2	1	2	4	2	3	4	4	3	3	3	2	6	5	6
26	1	Psychologie	2	2	3	3	3	3	3	3	3	3	3	3	3	3	3	3	3	4	4	4	4	4	4	3	3	3	3	3	4	4	4	3	3	3	3
22	3	5	1	1	2	2	3	2	4	2	2	5	2	2	5	6	5	4	4	2	5	1	2	2	3	6	2	2	6	6	1	1	1	2	3	3	2
21	2	Psychoologie	2	1	4	4	5	6	5	4	2	5	2	6	5	5	5	2	5	2	1	2	2	2	2	6	1	2	5	5	1	2	2	3	2	2	2
21	1	Online-Medien-Management	1	1	5	4	3	5	2	4	2	5	2	2	5	5	5	1	2	2	5	2	2	2	5	5	2	2	5	5	2	2	2	1	1	1	2
24	1	Psychologie (B.Sc.)	1	1	5	4	6	4	3	3	2	5	2	5	5	6	5	3	6	2	2	1	2	2	1	6	2	2	5	5	1	2	2	2	2	1	4
19	2	Psychologie	2	1	4	4	4	4	4	4	4	4	4	4	4	4	4	4	4	3	3	3	3	3	3	4	4	4	4	4	3	3	3	4	4	4	4
23	1	Kulturwirtschaft	3	2	4	3	1	4	1	4	2	5	2	5	5	6	5	1	6	2	2	1	2	2	1	6	1	2	3	4	1	4	3	2	2	1	2
25	1	Master Erziehungswissenschaften		2	6	5	5	5	3	5	1	6	2	5	5	6	5	2	4	1	2	1	2	2	3	6	2	3	6	6	1	1	1	3	3	2	3
22	1	Erziehungwissenschaft		1	4	6	5	3	5	5	1	5	2	5	5	5	5	2	6	2	2	2	2	2	1	5	2	3	3	4	2	4	3	2	2	2	2
27	1	Psychologie	3	2	4	3	5	4	3	4	2	6	1	4	6	5	6	1	6	1	3	2	1	1	1	6	2	1	6	6	1	1	1	2	2	1	2
23	2	Psychologie	1	1	3	3	3	3	3	3	3	3	3	3	3	3	3	3	3	4	4	4	4	4	4	3	3	3	3	3	4	4	4	3	3	3	3
23	1	Psychologie	2	2	5	4	4	6	3	5	2	6	2	6	5	6	6	2	6	1	1	1	2	1	1	6	2	3	5	5	1	2	2	3	3	2	5
21	1	Erziehungswissenschaft	3	2	4	2	5	3	3	3	2	5	2	5	5	5	5	2	4	2	2	2	2	2	3	5	2	2	5	5	2	2	2	2	2	2	2

22	1	B.A. Erziehungswissenschaft	2	1	5	4	4	2	2	4	2	5	2	2	5	6	5	2	4	2	5	1	2	2	3	6	1	2	6	6	1	1	1	3	5	4	5
40	1	psychologie	1	1	6	3	1	1	1	1	2	6	1	6	6	6	6	1	6	1	1	1	1	1	1	6	2	1	6	6	1	1	1	2	1	2	1
23	1	M.A. Erziehungswissenschaft	1	1	4	3	5	3	3	1	2	5	2	4	5	5	6	2	5	2	3	2	2	1	2	4	2	3	4	6	3	3	1	3	2	2	4
30	1	Psychologie	1	1	5	5	5	5	5	4	2	5	2	4	5	6	5	3	6	2	3	1	2	2	1	5	2	2	5	6	2	2	1	3	2	2	4
25	1	MA Prävention und Intervention in der Kindheit	2	1	5	6	4	5	4	5	3	4	3	4	4	4	4	2	4	3	3	3	3	3	3	5	2	3	4	4	2	3	3	3	2	2	3
24	1	Prävention und Intervention in der Kindhiet	3	2	6	2	6	3	2	4	2	5	2	6	6	5	5	1	5	2	1	2	1	2	2	5	1	2	6	6	2	1	1	2	2	1	2
20	1	Psychologie	1	1	6	5	6	5	2	4	2	5	2	4	5	6	5	2	6	2	3	1	2	2	1	6	1	3	6	6	1	1	1	3	3	2	4
56	2	Psychologie	2	1	5	6	6	2	6	2	2	5	2	6	5	5	5	1	5	2	1	2	2	2	2	5	2	2	5	5	2	2	2	3	4	2	5
25	1	Psychologie	2	1	6	4	5	5	4	4	2	6	2	6	6	5	6	2	5	1	1	2	1	1	2	5	2	3	4	5	2	3	2	2	3	2	3
18	2	Psychologie	3	2	6	6	6	6	6	6	6	6	6	6	6	6	6	6	6	1	1	1	1	1	1	6	6	6	6	6	1	1	1	6	6	6	6
19	1	Psychologie	1	1	3	5	2	5	3	3	2	6	2	5	5	5	5	3	5	1	2	2	2	2	2	5	2	2	5	5	2	2	2	2	2	3	3
24	1	Psychologie	1	1	5	4	4	5	3	4	3	4	3	2	3	4	3	4	4	3	5	3	4	4	3	5	2	3	4	5	2	3	2	2	2	2	3
28	1	Psychologie	1	1	5	2	5	1	3	1	2	4	2	3	5	5	4	3	3	3	4	2	2	3	4	4	3	4	3	3	3	4	4	2	2	1	2
19	2	Psychologie	1	1	6	5	6	5	3	3	2	6	2	5	5	6	6	1	5	1	2	1	2	1	2	6	2	2	5	6	1	2	1	2	2	2	3
23	1	Psychologie	3	2	4	4	3	3	2	2	2	6	2	6	5	5	5	4	6	1	1	2	2	2	1	5	2	2	6	6	2	1	1	1	1	1	1
22	1	Psychologie	1	1	4	6	5	3	2	2	1	5	5	5	4	3	4	3	3	2	2	4	3	3	4	5	4	2	3	3	2	4	4	4	3	2	5
51	1	Psychologie	1	1	4	3	3	4	3	3	2	5	2	5	5	6	5	2	4	2	2	1	2	2	3	6	1	2	4	6	1	3	1	2	2	2	3
21	1	Psychologie	1	1	6	5	4	5	5	5	2	4	2	4	5	5	5	2	5	3	3	2	2	2	2	4	3	3	4	5	3	3	2	4	3	3	5
37	1	Psychologie	3	2	6	3	2	3	4	3	2	6	1	6	6	6	6	2	6	1	1	1	1	1	1	6	1	6	6	6	1	1	1	1	2	1	4
21	1	Psychologie	3	2	5	5	2	2	4	3	3	2	5	2	3	5	2	4	3	5	5	2	4	5	4	3	4	5	4	3	4	3	4	1	4	2	3
37	2	Psychologie	3	1	6	5	6	6	5	6	2	5	2	6	5	5	5	3	5	2	1	2	2	2	2	6	2	2	4	5	1	3	2	5	5	4	5
22	1	Psychologie	2	1	4	3	3	4	2	4	2	5	3	4	4	4	4	3	5	2	3	3	3	3	2	5	3	3	4	5	2	3	2	3	2	3	2
21	2	Psychologie	1	1	6	5	6	4	4	5	2	4	3	4	4	4	4	4	5	3	3	3	3	3	2	4	3	4	4	5	3	3	2	4	4	4	6
22	1	Erziehungswissenschaft Ein-Fach Bachelor	3	2	6	5	6	6	4	6	2	4	3	6	5	6	6	3	5	3	1	1	2	1	2	5	2	3	5	5	2	2	2	5	4	4	5
22	1	Psychologie	1	1	5	2	2	2	1	2	2	6	1	6	6	6	6	3	6	1	1	1	1	1	1	6	1	1	6	6	1	1	1	3	2	2	2
19	1	Psychologie	2	1	4	3	3	4	3	3	1	5	1	5	5	5	5	2	5	2	2	2	2	2	2	4	2	2	5	5	3	2	2	3	6	1	4
22	1	Jura	3	1																																	
22	1	Psychologie	1	1	5	3	5	3	3	3	2	5	3	4	5	5	4	2	5	2	3	2	2	3	2	5	3	3	5	5	2	2	2	3	3	3	4
24	1	Sportwissenschaft und Pädagogik	1	1	5	4	6	4	3	3	2	5	2	5	5	5	5	2	4	2	2	2	2	2	3	3	3	3	4	5	4	3	2	3	3	3	4
33	1	General management	3	2	1	1	1	1	1	1	1	6	1	6	6	6	6	1	6	1	1	1	1	1	1	6	1	1	6	6	1	1	1	1	1	1	1
31	1	Psychologie	1	1	4	2	4	2	3	2	1	5	2	2	5	3	4	3	4	2	5	4	2	3	3	5	1	3	3	5	2	4	2	3	1	1	2
22	2	Psychologie	3	2	5	3	5	5	4	5	2	5	2	3	5	6	5	2	6	2	4	1	2	2	1	5	2	2	5	6	2	2	1	2	2	2	2
32	1	Erziehungswissenschaft	1	1																																	
24	1	Rehabilitatiionspsychologie	1	1	5	3	5	3	3	3	2	4	3	3	4	4	4	2	3	3	4	3	3	3	4	3	3	3	4	4	4	3	3	3	2	2	3
20	2	Psychologie Bachelor	1	1	4	4	3	4	3	3	2	5	2	5	5	5	5	2	4	2	2	2	2	2	3	5	2	2	5	5	2	2	2	3	3	3	5
23	2	M.A. EZW	3	2																																	
18	1	Psychologie	1	1	5	3	3	2	2	4	1	6	1	6	6	6	6	1	6	1	1	1	1	1	1	6	2	2	6	6	1	1	1	2	4	1	4
20	2	Psychologie	1	1	4	4	4	4	3	3	2	4	3	6	5	4	4	3	2	3	1	3	2	3	5	4	2	3	5	5	3	2	2	3	3	2	4
20	1	Psychologie	1	1	4	3	3	2	2	3	2	6	2	4	5	5	5	1	4	1	3	2	2	2	3	4	2	2	6	5	3	1	2	2	2	2	3
22	1	Psychologie	2	1	2	3	2	3	4	3	3	4	5	2	4	2	4	3	1	3	5	5	3	3	6	2	2	3	4	2	5	3	5	2	2	3	3
19	2	Bachelor Psychologie	1	1	4	4	4	4	2	3	1	6	1	5	6	6	6	1	6	1	2	1	1	1	1	6	1	2	6	6	1	1	1	2	1	2	2
19	1	Psychologie	1	1	6	1	5	2	3	3	2	6	1	6	6	6	6	1	6	1	1	1	1	1	1	6	1	2	4	5	1	3	2	1	2	1	2
24	1	Psychologie	3	2	4	3	3	3	1	3	3	2	3	3	1	5	3	4	3	5	4	2	6	4	4	2	3	5	2	3	5	5	4	2	2	4	3
21	1	Psychologie	3	2	4	3	4	5	4	4	2	5	2	5	6	6	6	2	5	2	2	1	1	1	2	3	2	2	5	5	4	2	2	2	2	2	3
24	2	Psychologie BA	1	1	4	2	5	5	2	4	2	5	2	4	5	6	6	2	6	2	3	1	2	1	1	3	2	3	5	6	4	2	1	2	2	2	3
21	1	Psychologie	2	1	5	4	5	2	3	4	2	6	2	5	6	6	6	2	6	1	2	1	1	1	1	5	1	3	6	6	2	1	1	3	1	1	3
24	1	B.A. Lehramt Gym/Ge	1	1	6	3	5	2	3	5	1	5	2	5	5	5	5	2	4	2	2	2	2	2	3	5	3	3	4	5	2	3	2	3	2	2	3
29	1	Wirtschaftspsychologie	3	2	5	2	5	1	3	4	2	5	5	4	5	5	5	1	5	2	3	2	2	2	2	5	2	2	4	5	2	3	2	2	2	2	2
20	2	Psychologie B.Sc.	1	1	5	3	5	2	2	2	2	6	1	6	6	6	6	1	6	1	1	1	1	1	1	6	1	2	6	6	1	1	1	1	1	1	1
27	1	Psychologie	1	1																																	
21	1	psy	1	1																																	
22	1	Psychologie	1	1	5	4	5	3	3	4	2	5	2	4	6	6	6	1	4	2	3	1	1	1	3	6	1	2	6	6	1	1	1	2	2	3	2
34	1	Psychologie	3	2																																	
30	1	Psychologie Bachelor	1	1																																	
34	1	B.Sc. Psychologie	1	1	6	5	1	5	5	6	4	3	4	5	3	3	3	4	4	4	2	4	4	4	3	4	3	4	4	4	3	3	3	3	6	6	6
22	2	Psychologie	3	1																																	
33	1	Wirtschaftsinformatik	3	2	4	3	2	4	2	4	2	5	2	4	5	6	5	2	6	2	3	1	2	2	1	6	2	2	4	6	1	3	1	2	2	2	2
22	2	Psychologie	2	1	1	1	1	1	1	1	1	1	1	1	1	1	1	1	1	6	6	6	6	6	6	1	1	1	1	1	6	6	6	1	1	1	1
21	2	Psychologie	1	1																																	
24	1	Psychologie	3	2	2	3	4	2	2	3	2	3	4	4	1	3	3	3	4	4	3	4	6	4	3	1	4	3	6	2	6	1	5	3	4	3	2
27	1	Psychologie	3	1	6	5	6	6	3	6	1	6	2	6	6	6	6	2	5	1	1	1	1	1	2	3	2	2	2	5	4	5	2	1	3	4	4
34	1	Bachelor Psychologie	3	2	4	3	5	3	3	3	1	6	2	6	6	6	6	2	4	1	1	1	1	1	3	6	1	2	6	6	1	1	1	1	2	2	5
30	1	Psychologie	1	1	5	5	4	3	3	4	1	6	2	6	5	6	5	3	5	1	1	1	2	2	2	4	2	3	4	5	3	3	2	4	4	3	6
24	1	Psychologie	1	1	4	4	4	4	4	4	3	3	3	4	3	4	3	3	6	4	3	3	4	4	1	3	3	4	4	3	4	3	4	2	2	2	2
20	1	Psychologie	1	1	5	4	4	4	3	3	2	5	2	5	5	5	5	2	5	2	2	2	2	2	2	6	2	2	6	6	1	1	1	2	2	2	2
21	1	Psychologie	1	1	5	2	5	2	3	2	2	5	2	6	5	5	5	2	5	2	1	2	2	2	2	6	2	2	5	5	1	2	2	2	2	2	3
47	1	Psychologie	1	1	6	6	5	6	4	6	2	5	2	5	5	5	5	2	5	2	2	2	2	2	2	5	2	2	5	5	2	2	2	4	4	4	4
28	2	Psychologie	2	1	6	5	5	6	3	5	2	5	2	6	6	5	5	2	5	2	1	2	1	2	2	6	2	2	5	5	1	2	2	3	5	4	6
36	3	Psychologie Bachelor	1	1	5	3	5	4	2	5	2	6	2	5	5	6	5	2	6	1	2	1	2	2	1	5	2	3	3	4	2	4	3	2	5	4	5
18	1	Psychologie	1	1	4	4	4	6	3	4	2	6	2	5	6	6	5	2	5	1	2	1	1	2	2	6	1	2	6	6	1	1	1	3	2	2	4
21	1	Lehramt an Haupt-, Real-, Sekundar- und Gesamtschulen (B.A.)	1	1	5	3	2	3	2	3	1	5	2	4	5	6	5	2	5	2	3	1	2	2	2	5	1	2	5	6	2	2	1	3	3	3	4
24	2	Humanmedizin	3	2	4	4	3	4	4	4	2	5	2	5	5	6	5	4	3	2	2	1	2	2	4	6	1	2	5	5	1	2	2	3	4	4	6
23	2	Psychologie	3	2																																	
28	1	Bachelor Psychologie	3	1	3	4	3	4	3	2	4	3	3	5	4	4	3	4	2	4	2	3	3	4	5	5	2	3	3	3	2	4	4	3	2	5	3
24	1	Psychologie	3	2	4	3	4	4	3	4	2	5	2	6	6	6	6	2	6	2	1	1	1	1	1	6	2	2	6	6	1	1	1	2	2	2	3
30	1	Erziehungswissenschaft 1-fach	2	2	6	3	6	4	4	3	1	6	1	6	6	6	6	2	6	1	1	1	1	1	1	6	1	2	6	6	1	1	1	2	2	3	4
19	1	Psychologie	1	1	5	3	4	5	5	2	2	2	3	2	3	3	4	4	6	5	5	4	4	3	1	3	3	3	6	5	4	1	2	1	4	5	3
21	1	Psychologie	1	1	5	4	5	5	3	5	2	5	2	4	5	5	5	2	5	2	3	2	2	2	2	5	3	3	5	5	2	2	2	5	5	2	1
24	1	PSYCHOLOGIE	3	2	6	3	6	4	4	6	2	5	2	4	5	6	5	2	6	2	3	1	2	2	1	6	2	2	6	6	1	1	1	2	1	1	2
21	1	BA Lehramt	1	1	4	3	4	3	2	3	2	4	2	3	4	4	4	2	3	3	4	3	3	3	4	4	2	3	4	4	3	3	3	2	2	2	3
23	1	Lehramt HRSGe	1	1	3	3	4	3	3	3	3	4	3	3	3	3	3	3	3	3	4	4	4	4	4	4	3	3	4	3	3	3	4	3	3	3	3
25	2	Lehramt, Mathe & Physik	3	2	6	3	4	2	4	3	1	5	2	3	6	5	5	1	3	2	4	2	1	2	4	5	2	3	5	5	2	2	2	2	2	2	2
23	2	Psychologie	3	2	5	2	4	5	2	3	2	5	2	5	5	5	5	2	3	2	2	2	2	2	4	5	1	2	5	5	2	2	2	2	2	1	2
22	1	Psychologie	1	1	5	4	3	3	2	4	3	4	2	2	6	6	6	3	6	3	5	1	1	1	1	6	2	2	6	6	1	1	1	3	2	2	4
24	2	Ba GymGe Geschichte/Philosophie	1	1	4	4	4	3	3	3	2	4	3	3	4	3	3	3	3	3	4	4	3	4	4	4	3	3	3	4	3	4	3	3	4	4	4
20	1	Psychologie	1	1	4	2	2	2	2	2	2	5	4	6	5	5	5	2	6	2	1	2	2	2	1	6	2	2	6	6	1	1	1	2	1	1	2
25	1	Psychologie	2	1	5	5	4	4	3	3	1	6	1	5	6	6	6	2	6	1	2	1	1	1	1	5	2	3	5	5	2	2	2	4	2	2	2
34	1	B.Sc. Psychologie	1	1																																	
24	1	Psychologie	2	2																																	
40	1	Psychologie	1	1																																	
30	1	Philosophie/Jura	2	1																																	
19	1	Grundschullehramt	1	1	6	3	5	2	3	2	2	5	3	5	5	5	5	2	3	2	2	2	2	2	4	5	2	2	3	3	2	4	4	2	2	3	5
23	1	B. Sc. Psychologie	3	2	5	4	5	6	4	5	2	4	3	4	4	5	4	2	6	3	3	2	3	3	1	6	2	2	6	6	1	1	1	2	5	2	5
22	1	Psychologie	1	1																																	
24	2	Angewandte Kognitions- und Medienwissenschaft	3	2	5	4	5	5	3	5	2	4	2	4	5	4	4	2	3	3	3	3	2	3	4	5	1	2	4	4	2	3	3	3	2	2	3
27	1	Marketing	1	1	3	3	1	1	1	1	1	6	1	2	6	6	6	1	3	1	5	1	1	1	4	5	2	1	6	6	2	1	1	1	1	1	1
23	1	Psychologie	3	1	4	2	3	3	4	3	6	5	2	4	2	1	5	4	6	2	3	6	5	2	1	3	2	3	5	2	4	2	5	5	5	2	5
22	1	Psychologie	3	2	4	2	4	3	3	3	2	4	3	3	4	5	4	2	3	3	4	2	3	3	4	5	2	2	4	5	2	3	2	2	2	2	2
21	1	Medienkommunikation	2	2	6	5	4	3	3	3	2	5	2	5	5	6	5	4	6	2	2	1	2	2	1	5	2	2	4	5	2	3	2	3	4	3	5
22	1	Medienkommunikation	3	2	5	3	4	3	4	3	1	6	1	6	6	6	6	1	5	1	1	1	1	1	2	6	1	2	6	6	1	1	1	3	2	2	2
22	1	Master Wirtschaftspsychologie	1	1	2	2	1	3	1	2	1	6	1	6	6	6	6	1	6	1	1	1	1	1	1	6	1	1	6	6	1	1	1	1	1	1	1
24	1	Master of Management & Marketing	3	2	5	2	4	3	3	2	2	5	3	3	5	5	5	3	5	2	4	2	2	2	2	5	2	2	5	5	2	2	2	2	2	1	2
24	2	BWL	3	2	5	3	2	4	1	3	2	2	4	2	5	3	4	2	4	5	5	4	2	3	3	5	4	2	4	4	2	3	3	2	3	3	3
21	1	Medien- und Wirtschaftspsychologie	3	2	6	4	5	4	4	4	2	5	2	4	5	5	5	2	5	2	3	2	2	2	2	5	2	2	5	5	2	2	2	2	3	3	4
52	1	Psychologie Master	3	2	4	4	4	3	2	3	2	5	2	6	6	6	5	2	5	2	1	1	1	2	2	6	2	2	6	6	1	1	1	2	2	2	2
36	1	B.Sc. Psychologie	3	2	6	3	5	5	4	2	2	4	3	5	4	5	4	3	6	3	2	2	3	3	1	4	2	2	4	4	3	3	3	3	3	4	5
25	1	Wiwi	3	2	4	2	5	2	2	4	3	5	2	5	4	5	5	2	2	2	2	2	3	2	5	4	2	2	6	6	3	1	1	2	3	3	6
25	2	Wirtschaftswissenschaften	3	2	5	3	5	3	2	2	1	6	3	6	6	6	6	3	6	1	1	1	1	1	1	5	2	3	6	6	2	1	1	3	2	2	2
22	1	Medienkommunikation	3	2	2	3	6	4	4	4	2	6	5	6	6	6	5	2	6	1	1	1	1	2	1	5	2	2	5	6	2	2	1	2	2	3	2
24	2	Verkehrsingenieur und Mobilität	1	1	5	2	5	3	3	3	2	6	3	6	5	4	5	2	6	1	1	3	2	2	1	6	1	3	6	6	1	1	1	3	5	5	5
28	1	Psychologie		2	5	4	3	5	2	5	3	4	3	2	3	4	3	2	3	3	5	3	4	4	4	4	3	2	3	4	3	4	3	2	2	2	5
21	1	Psychologie	3	2																																	
25	1	BWL		2	1	1	1	4	3	3	1	1	2	1	2	2	2	1	2	6	6	5	5	5	5	1	1	1	3	2	6	4	5	1	4	4	1
22	1	Psychologie	2	1	4	4	6	2	3	5	2	3	3	3	4	3	4	2	1	4	4	4	3	3	6	1	6	4	1	1	6	6	6	3	4	5	4
31	2	M.Sc. Applied Statistics	3	2	5	4	5	6	3	5	2	5	3	5	5	5	5	2	5	2	2	2	2	2	2	6	1	1	6	6	1	1	1	6	6	5	6
25	1	Wirtschaftspsychologie		2	4	3	6	5	3	4	1	5	1	5	6	6	6	1	6	2	2	1	1	1	1	6	1	2	6	6	1	1	1	1	2	2	2
27	1	Psychologie	1	1	6	6	6	6	1	6	1	6	1	6	6	6	6	1	6	1	1	1	1	1	1	6	1	1	6	6	1	1	1	1	1	6	1
24	2	Wirtschaftsinformatik	2	2	5	5	4	6	4	5	2	5	3	5	5	5	5	2	5	2	2	2	2	2	2	4	3	2	5	4	3	2	3	2	2	2	3
24	1	BWL	3	2	5	3	3	4	2	3	2	4	2	6	5	6	5	1	5	3	1	1	2	2	2	4	2	2	4	5	3	3	2	3	1	2	3
23	1	Wirtschaftspsychologie	1	1	6	3	5	1	3	3	3	3	3	4	4	4	4	3	4	4	3	3	3	3	3	4	2	3	4	4	3	3	3	3	2	2	6
31	1	Psychologie	2	1	5	3	3	3	2	3	2	6	1	5	6	5	5	2	5	1	2	2	1	2	2	6	1	1	6	6	1	1	1	3	2	1	2
26	1	Marketing	3	1	5	3	5	4	2	4	2	5	2	5	5	5	5	3	5	2	2	2	2	2	2	5	2	2	5	5	2	2	2	2	2	2	2
26	1	Psychologie	3	2	5	5	1	4	2	3	1	6	1	6	6	6	6	1	6	1	1	1	1	1	1	6	1	1	6	6	1	1	1	1	1	1	1
42	1	Psychologie (B.Sc.)	3	1	4	2	4	5	2	3	2	4	2	5	5	5	5	2	6	3	2	2	2	2	1	4	2	2	6	5	3	1	2	2	2	2	2
26	1	Medienmanagement		2	4	5	4	4	3	3	1	6	1	3	6	6	6	1	2	1	4	1	1	1	5	6	1	1	6	6	1	1	1	2	2	2	2
28	1	Kognitionswissenschaften	3	2	4	4	5	5	3	5	2	4	3	4	4	4	4	2	4	3	3	3	3	3	3	4	2	3	4	5	3	3	2	2	4	4	4
23	1	BWL	1	1	5	2	5	5	4	4	2	6	3	6	6	5	4	2	3	1	1	2	1	3	4	6	2	3	6	6	1	1	1	3	4	4	5
25	1	Marketing	3	2	5	2	4	3	4	3	2	5	3	4	5	6	5	2	4	2	3	1	2	2	3	3	2	3	4	5	4	3	2	3	3	1	3
26	1	Psychologie	1	1	3	2	3	2	2	2	2	4	2	4	5	5	5	1	3	3	3	2	2	2	4	4	2	2	5	5	3	2	2	2	2	1	2
24	1	Marketing	3	2	5	5	3	5	1	2	2	4	3	4	6	6	6	1	3	3	3	1	1	1	4	6	3	2	6	5	1	1	2	1	2	2	2
22	1	Medien- und Wirtschaftspsychologie	2	1	4	3	5	2	3	2	2	4	2	6	4	5	5	2	6	3	1	2	3	2	1	5	1	2	5	6	2	2	1	2	4	3	4
23	1	Wirtschaftsinformatik	3	2	5	4	3	2	3	4	4	5	4	5	2	3	5	4	3	2	2	4	5	2	4	4	3	3	4	3	3	3	4	4	4	4	3
25	1	Marktfoschung	3	2	4	3	4	2	1	2	1	6	1	6	6	6	6	1	6	1	1	1	1	1	1	6	1	1	6	6	1	1	1	2	1	1	2
32	1	Soziale Arbeit	3	2	6	6	5	6	4	6	1	6	1	3	6	6	6	1	6	1	4	1	1	1	1	5	2	4	3	6	2	4	1	6	6	6	6
23	2	Psychologie	2	1	3	3	5	5	2	2	3	1	1	2	2	2	6	2	5	6	5	5	5	1	2	3	5	4	2	5	4	5	2	4	2	2	2
36	1	Psychologie	3	2	5	4	3	3	2	3	2	6	5	6	6	6	6	2	4	1	1	1	1	1	3	5	2	4	6	5	2	1	2	2	3	2	3
18	1	Psychologie	3	2	4	4	5	6	2	5	3	3	3	6	4	5	3	5	3	4	1	2	3	4	4	3	2	3	5	5	4	2	2	2	4	2	5
31	2	Wirtschaftswissenschaften	3	2	5	3	5	5	4	3	2	5	2	6	5	5	4	1	6	2	1	2	2	3	1	5	2	2	5	5	2	2	2	3	4	3	5
23	1	Psychologie	1	1	5	2	4	3	5	2	3	4	3	2	5	3	4	2	3	3	5	4	2	3	4	3	2	2	3	3	4	4	4	1	1	1	1

25	1	BWL	3	2	5	1	5	5	1	1	1	5	2	1	5	6	5	1	1	2	6	1	2	2	6	4	1	1	6	5	3	1	2	1	1	1	2
23	1	Medienwissenschaft	3	2	4	5	3	4	2	4	1	5	2	5	5	6	6	2	5	2	2	1	2	1	2	5	1	1	6	6	2	1	1	1	2	1	2
22	1	Psychologie	2	1	3	2	5	2	2	2	2	5	2	4	5	4	4	2	4	2	3	3	2	3	3	4	2	2	4	5	3	3	2	3	2	2	4
22	2	B.Sc. Psychologie	3	2	5	4	5	4	3	4	2	5	2	4	5	5	5	2	5	2	3	2	2	2	2	5	2	2	5	5	2	2	2	2	1	1	2
20	1	Psychologie	1	1	6	3	5	4	5	4	1	6	5	6	6	6	6	2	6	1	1	1	1	1	1	5	1	2	6	6	2	1	1	5	4	4	5
22	1	Medienkommunikation (Uni Würzburg)	3	2	6	3	5	4	2	4	1	6	1	6	6	6	6	1	6	1	1	1	1	1	1	6	1	1	6	6	1	1	1	1	1	1	2
26	2	Management	2	1	6	4	2	1	4	2	3	6	2	6	5	5	4	2	4	1	1	2	2	3	3	4	2	1	4	5	3	3	2	2	2	2	6
23	1	Business Administration	3	2	3	3	4	3	3	3	2	5	2	5	5	5	5	1	5	2	2	2	2	2	2	5	1	2	4	5	2	3	2	3	2	2	3
23	1	Psychologie	2	2	6	6	5	6	4	5	6	6	6	6	2	5	5	6	6	1	1	2	5	2	1	5	4	5	6	2	2	1	5	6	5	5	4
33	1	Pädagogik	2	1	4	5	4	4	4	4	2	5	2	4	5	5	5	2	4	2	3	2	2	2	3	5	2	2	4	5	2	3	2	2	2	2	2
25	1	bwl	3	2	6	3	6	4	2	5	4	4	3	2	4	5	4	3	2	3	5	2	3	3	5	4	2	3	5	5	3	2	2	2	2	4	4
26	1	Tourismusmanagement	3	2	5	1	5	1	4	5	2	5	2	4	6	6	6	1	5	2	3	1	1	1	2	6	2	2	6	6	1	1	1	3	2	1	2
35	1	Psychologie	1	1	4	3	3	3	2	3	2	5	2	5	5	5	5	2	4	2	2	2	2	2	3	6	2	2	4	6	1	3	1	2	2	2	3
18	1	Grundschullehramt	3	2	4	3	4	4	3	4	2	4	3	4	4	4	4	3	4	3	3	3	3	3	3	3	2	2	5	4	4	2	3	2	3	2	3
48	1	Psychologie Master	3	2	4	3	3	2	1	3	1	6	1	4	6	6	6	1	3	1	3	1	1	1	4	6	1	1	6	6	1	1	1	1	1	1	2
23	1	B.Sc Psychologie	3	2	5	5	4	4	4	5	2	4	3	4	3	3	3	4	3	3	3	4	4	4	4	4	3	4	3	3	3	4	4	2	2	2	2

LZ5	LZ6	LZ7	MOG1	MOG2	MOG3	MOG4	MOG5	MOG6	MOG7	MOG8	MOG9	MOG10	MOG11	MOG12	MOG13	MOG14	MOG15	PGWG1	PGWG3	PGWG2	PGWG4	PGWG5	PGWG6	PGWG7	APSI_P1	APSI_P2	APSI_P3	APSI_P4	APSI_P5	APSI_P6	APSI_P7	APSI_P8	APSI_P9	APSI_P10	APSI_P11	APSI_P12	APSI_II13	APSI_II14	APSI_II15
5	5	5	1	2	3	3	3	4	3	4	4	4	4	3	4	3	3	6	5	5	5	5	5	5	1	2	3	3	2	3	5	5	5	5	3	5	5	5	5
6	6	6	5	5	5													6	6	6	6	6	6	6	5	5	5	5	5	5	5	5	5	5	5	5	5	5	5
6	6	6	4	5	4	4	4	4	4	4	4	4	4	4	4	4	4	6	6	6	6	6	6	6	5	5	5	5	5	5	5	5	5	5	5	2	5	5	5
1	1	2	3	3	3	3	3	3	3	3	3	3	3	3	3	3	3	5	2	2	2	5	2	3	3	2	3	3	3	3	3	3	2	4	3	4	3	2	2
2	1	2	6	6	6	5	6	6	6	6	3	6	6	6	6	6	6	2	3	2	3	3	2	1	4	3	2	4	4	1	3	1	2	4	1	1	2	3	1
1	1	2	6	6	5	6	6	6	6	6	6	6	6	6	6	6	6	3	2	3	2	1	2	2	2	2	2	2	2	1	1	1	1	2	2	1	3	2	4
2	2	4	2	4	5	3	4	4	6	3	2	5	4	3	3	2	4	3	5	4	3	1	2	1	2	4	2	3	3	4	4	1	3	5	4	2	3	2	2
2	2	2	6	6	6	6	6	6	6	6	6	6	6	6	6	6	6	2	2	2	2	2	2	2	1	3	2	2	2	1	1	2	4	1	1	2	1	1	1
3	1	3	6	6	3	6	6	6	5	5	6	5	6	6	6	6	6	3	4	3	4	4	4	3	3	3	3	3	3	3	3	3	5	3	2	1	4	3	2
4	3	2	6	3	6	3	6	6	6	6	5	5	6	6	6	6	6	4	2	2	2	4	4	3	4	4	3	4	3	3	3	3	3	3	3	2	2	2	3
4	3	4	6	6	5	6	6	6	6	6	6	6	6	6	6	6	6	3	5	5	5	5	5	5	5	5	5	5	5	5	3	4	2	5	3	2	3	3	3
1	1	2	6	6	6	6	6	6	6	6	6	5	6	6	5	6	6	6	4	1	1	1	1	1	3	3	3	4	4	3	2	1	3	3	2	1	3	1	2
2	1	2	6	6	6	6	6	6	6	6	6	6	6	6	6	6	6	4	2	2	2	2	2	2	3	3	3	3	4	3	2	4	3	3	3	2	3	3	2
4	3	4	6	4	6	4	5	6	6	6	6	5	6	6	6	6	6	2	3	2	3	4	4	4	3	2	2	4	2	4	1	2	4	3	1	2	2	1	2
1	1	1	6	5	3	4	5	6	6	6	6	6	6	6	6	6	6	4	1	1	1	1	1	1	1	1	1	1	1	1	3	1	5	1	1	1	1	2	1
1	1	1	6	6	3	6	6	6	6	6	6	6	6	5	6	6	6	2	1	1	1	1	2	2	3	3	2	1	2	2	1	1	4	2	2	1	2	2	1
2	2	2	6	4	3	4	5	6	4	3	3	4	6	5	5	6	6	2	2	3	2	2	3	2	3	3	4	3	4	2	2	2	4	3	3	2	3	2	4
2	2	2	6	6	6	6	6	6	6	6	6	6	6	6	6	6	6	2	2	4	3	4	3	2	3	2	2	2	3	3	1	1	4	3	1	1	2	2	3
3	2	2	6	6	6	6	6	6	6	6	6	5	6	6	6	6	6	2	2	2	2	2	2	2	3	3	3	3	3	3	3	3	3	3	2	2	2	2	1
3	1	2	6	6	5	6	6	6	6	6	6	6	6	6	6	6	6	4	3	2	2	2	2	2	2	1	2	1	4	3	2	3	4	3	3	2	3	3	1
4	3	5	6	2	3	5	5	6	5	4	3	6	6	6	6	6	6	3	3	2	2	3	3	2	3	4	3	3	3	4	2	2	3	2	3	1	4	3	5
1	1	1	6	5	4	6	6	6	6	5	6	6	6	6	6	6	6	1	1	1	1	1	1	1	2	4	2	2	2	1	2	1	4	3	1	1	2	2	1
3	1	2	6	5	5	6	6	6	6	6	6	6	6	5	6	6	6	2	2	2	3	2	2	2	4	4	5	4	4	5	3	3	4	4	5	2	4	2	2
4	1	3	6	4	3	6	6	6	6	6	4	6	6	6	6	6	5	2	2	3	2	4	6	3	4	4	4	5	4	3	3	3	2	2	5	2	4	3	2
2	1	1	6	6	5	3	6	6	6	6	6	5	6	5	5	5	6	1	2	2	2	2	2	2	3	3	3	3	4	2	3	1	3	3	5	3	3	2	2
2	2	2	5	5	3	5	6	6	6	6	5	5	6	6	6	6	6	2	3	2	3	2	3	2	3	3	2	4	3	3	3	3	4	4	3	3	2	1	1
1	2	2	6	3	6	6	6	6	5	6	6	6	6	6	6	6	6	2	2	2	2	2	2	2	1	1	2	2	2	1	1	2	5	1	4	1	2	1	4
1	1	2	6	3	6	5	6	6	6	6	6	6	6	6	6	6	6	2	3	2	3	2	2	3	1	3	3	2	2	1	2	3	4	2	3	2	3	1	1
3	2	2	6	5	6	6	6	6	6	6	5	6	6	6	6	6	6	3	2	3	2	4	3	3	4	4	4	2	4	3	2	3	3	4	2	1	4	2	4
1	2	1	6	6	5	6	6	6	6	6	6	6	6	6	6	6	6	2	2	1	1	1	1	2	2	3	3	2	3	2	2	3	4	3	3	2	3	2	1
3	1	4	6	5	2	6	6	5	6	3	3	6	6	3	6	6	6	3	2	2	3	2	2	2	3	3	4	4	3	4	3	3	2	4	4	4	4	2	3
4	4	5	6	6	6	5	6	6	6	6	6	4	6	6	6	6	6	4	3	5	5	6	5	4	2	2	2	2	3	2	2	3	4	3	3	2	3	2	1
6	1	1	6	6	5	6	6	6	6	6	5	6	6	6	6	6	6	6	3	5	6	5	4	4	2	3	1	3	3	2	4	2	5	3	2	2	3	3	1
2	3	3	6	5	6	6	6	6	6	5	6	6	6	6	6	6	6	2	2	2	2	2	2	2	3	3	3	4	4	3	3	4	2	4	2	3	3	2	3
1	2	2	6	2	4	5	5	6	6	5	5	4	6	6	5	6	6	2	2	2	2	2	1	1	3	3	3	2	3	2	2	2	4	3	3	2	3	2	3
1	1	2	6	6	6	6	6	6	6	6	6	5	6	6	6	6	6	2	3	3	2	3	2	2	3	2	2	3	3	3	3	2	4	3	3	3	3	3	3
2	2	3	6	5	4	6	5	6	3	3	6	6	6	5	6	6	6	2	1	1	1	1	1	1	3	4	4	4	3	2	3	2	5	1	1	1	3	2	2
3	1	4	6	2	4	6	6	6	5	6	6	6	6	5	6	6	6	5	2	3	5	5	5	3	4	4	4	3	5	2	4	4	3	4	4	4	4	4	3
2	2	3	6	6	6	6	6	6	6	6	6	6	6	6	6	6	6	3	2	4	5	5	2	3	2	2	3	3	4	4	2	3	4	3	2	2	2	2	2
3	2	4	6	6	6	6	6	6	6	6	2	6	6	5	6	6	6	2	2	2	3	2	2	2	4	4	4	4	4	4	4	4	3	2	4	3	4	4	5
3	3	2	6	2	5	6	6	6	6	5	5	6	6	6	6	6	6	2	3	3	3	3	2	3	3	4	4	3	4	4	2	3	5	3	4	1	1	2	1
4	2	3	6	5	6	6	6	6	6	6	6	6	6	6	6	6	6	3	4	4	4	4	4	4	2	3	3	3	3	3	2	4	4	3	2	2	2	2	2
4	4	4	3	4	3	6	6	6	5	4	3	3	6	3	3	6	3	3	3	3	4	3	3	3	4	5	4	4	4	3	2	3	4	3	2	2	3	3	4
2	2	2	6	5	6	6	6	6	6	6	5	6	6	6	6	6	6	2	2	2	1	2	2	2	3	3	3	2	2	2	2	1	5	3	2	1	3	2	2
4	1	2	6	6	6	6	6	6	6	6	6	6	6	6	6	6	6	2	3	3	2	3	3	3	4	4	3	2	3	3	4	3	3	4	4	2	3	3	1
2	2	2	6	5	5	5	5	5	6	5	5	6	6	6	6	6	6	2	2	2	2	2	2	2	3	3	3	2	1	2	2	4	2	3	3	2	3	2	2
1	2	2	6	2	3	2	2	4	4	4	3	3	6	6	6	6	2	3	3	2	2	3	2	2	4	5	5	5	5	3	2	2	3	5	4	3	4	3	4
4	4	6	6	2	3	5	6	6	3	2	6	4	6	5	6	6	6	2	4	4	5	5	5	5	5	5	4	2	4	4	3	4	2	4	4	4	4	3	4
2	1	1	6	6	6	6	6	6	6	6	6	6	6	6	6	6	6	2	3	2	2	2	2	2	1	3	4	4	3	3	3	3	4	4	3	3	4	2	1
2	1	1	6	6	5	6	6	6	6	6	6	6	6	6	6	6	6	4	2	2	2	2	2	2	3	3	3	3	3	3	3	3	3	3	3	2	2	3	2
2	1	1	6	5	6	6	6	6	6	6	6	6	6	6	6	6	6	1	1	1	1	2	1	2	2	3	3	2	2	1	2	1	4	2	2	1	1	2	1
4	2	3	6	2	3	6	6	6	5	3	3	2	6	4	2	6	6	1	4	4	4	3	3	3	2	3	4	3	5	4	5	5	4	5	2	1	2	4	5
2	1	2	6	6	6	6	6	6	6	6	6	5	6	6	6	6	6	2	2	2	2	2	2	2	3	3	3	3	3	2	2	1	4	2	1	2	3	1	1
3	2	2	6	4	5	4	4	5	5	5	4	5	6	6	6	6	6	2	3	2	2	2	2	2	5	4	4	3	4	4	4	3	3	4	4	4	5	5	5
1	1	1	6	6	6	6	6	6	6	6	6	6	6	6	6	6	6	2	2	2	3	5	2	2	4	4	4	3	3	2	3	2	4	4	3	2	3	3	2
3	2	2	6	5	4	5	5	6	5	5	6	6	6	6	6	6	6	2	2	2	2	1	1	2	2	2	2	3	3	2	4	2	4	2	2	1	3	2	1
3	2	3	6	6	5	6	6	6	6	6	6	6	6	6	6	6	6	2	2	3	2	3	3	2	4	3	4	3	4	3	2	4	3	4	4	2	3	1	1
1	1	1	6	6	5	6	6	6	6	6	6	6	6	6	6	6	6	2	1	1	1	1	1	1	2	3	2	3	4	3	4	1	5	2	1	1	1	3	1
2	1	2	6	5	6	6	6	6	6	6	6	6	6	6	6	6	6	3	2	2	2	3	2	2	3	3	3	3	3	3	4	2	4	3	3	2	3	3	2
2	2	4	6	6	6	6	6	6	6	5	6	3	6	4	6	6	6	3	2	2	3	3	3	3	3	4	4	4	5	4	3	4	2	4	2	3	4	4	2
1	1	1	6	5	5	6	6	6	6	5	4	6	6	5	6	6	6	2	2	2	2	2	2	3	4	4	3	3	2	3	2	3	3	5	4	2	3	3	2
2	1	2	6	3	4	3	6	6	4	5	3	5	6	6	6	6	6	2	2	2	1	2	1	2	4	5	4	3	3	4	4	3	4	4	3	1	3	2	2
4	1	3	5	6	4	6	6	6	6	3	3	4	6	6	5	6	2	5	4	2	2	3	2	2	4	4	4	4	3	3	2	3	3	4	5	2	3	3	1
1	2	2	6	5	6	6	6	6	6	6	6	2	6	6	3	6	6	2	2	2	2	3	2	2	3	4	4	3	3	4	3	2	4	5	3	3	4	2	1
2	2	2	6	6	6	6	6	6	6	6	6	6	6	5	6	6	6	2	2	2	1	1	1	1	3	3	3	2	3	3	2	3	4	3	3	2	3	2	3
1	2	3	6	6	6	6	6	6	6	6	6	5	6	6	5	6	6	2	3	4	2	4	3	2	3	4	3	2	3	3	3	3	4	2	2	2	3	3	2
2	2	1	6	6	6	6	6	6	6	6	6	6	6	6	6	6	6	2	3	3	3	5	2	2	3	4	4	3	3	3	3	4	3	3	4	3	3	2	2
1	2	3	5	5	3	5	5	6	5	3	2	6	6	5	6	6	6	2	2	2	2	1	2	2	4	4	3	4	3	5	2	2	4	4	2	1	2	1	1
1	1	2	6	6	6	6	6	6	6	6	6	6	6	6	6	6	6	5	5	4	5	5	5	4	2	2	2	1	2	1	2	2	4	1	2	1	2	2	4
2	2	2	6	3	3	5	6	6	5	6	6	6	6	6	6	6	6	3	2	2	3	3	2	3	2	2	3	1	4	2	4	1	5	2	3	3	3	3	4
1	2	1	6	3	4	4	4	6	5	3	3	6	6	5	6	6	6	3	2	2	2	2	3	3	3	5	5	4	3	3	3	3	3	4	5	2	5	2	1
1	6	5	3	1	3	5	2	4	3	2	3	6	4	5	3	4	6	5	4	3	3	2	5	6	3	2	4	3	5	1	3	2	4	3	2	2	5	1	2
2	2	4	6	2	3	6	6	6	6	5	3	4	6	5	5	6	6	3	4	4	3	4	3	3	4	3	3	2	3	2	4	2	2	3	3	2	3	1	4
2	1	2	4	6	2	6	6	6	6	6	6	6	6	6	6	6	6	1	2	4	3	3	3	4	4	3	3	4	2	1	3	2	3	2	3	3	3	2	2
3	3	3	3	3	3	3	3	3	3	3	3	3	3	3	3	3	3	3	3	3	3	3	3	3	3	3	3	3	3	3	3	3	3	3	3	3	3	3	3
3	2	2	6	6	5	5	6	6	6	6	6	6	6	6	6	6	5	2	2	2	2	1	2	2	3	3	3	4	4	3	2	3	4	4	3	3	2	3	2
1	1	2	6	6	6	6	6	6	6	6	6	6	6	6	6	6	6	6	3	2	3	2	2	2	2	2	2	2	2	2	3	2	2	2	2	2	3	2	2
5	2	3	6	6	6	6	6	6	6	6	6	6	6	6	6	6	5	2	2	2	2	3	2	3	4	4	4	4	3	3	4	4	2	2	3	3	3	2	2
2	1	3	6	6	6	6	6	6	6	6	6	6	6	6	6	6	6	3	3	4	4	6	3	3	4	4	4	3	4	3	3	2	2	3	3	2	4	2	3
2	1	2	6	6	6	6	6	6	6	6	6	5	6	5	6	6	6	5	2	4	3	5	2	2	2	3	3	3	3	3	2	3	3	1	2	2	3	2	2
2	2	2	6	6	6	6	6	6	6	6	6	6	6	6	6	6	6	2	2	2	3	3	3	3	3	3	3	2	3	1	2	1	4	3	3	1	2	2	1
3	2	2	6	3	3	5	6	6	6	5	6	2	6	6	3	6	6	2	2	5	2	1	3	2	3	4	4	3	4	3	4	3	3	1	4	4	4	4	1
1	1	6	6	2	1	2	6	6	2	4	4	6	6	6	6	6	3	4	3	2	2	2	2	2	3	3	3	4	4	3	2	2	4	3	3	2	5	3	1
3	1	2	6	6	1	6	6	6	6	6	6	4	6	4	5	5	6	2	2	2	2	2	2	2	2	2	2	2	2	1	2	2	4	3	3	2	2	2	1
2	2	2	6	3	5	6	6	6	6	6	6	6	6	6	6	6	6	3	2	2	2	2	2	1	2	3	2	2	3	3	2	3	4	4	3	2	2	1	1
6	6	6	6	6	6	6	6	6	6	6	6	6	6	6	6	6	6	6	6	6	6	6	6	6	5	5	5	5	5	5	5	5	5	5	5	5	5	5	5
2	1	1	6	5	4	6	6	6	6	6	6	5	6	6	5	6	6	2	2	1	2	1	2	2	3	3	3	2	2	2	2	3	4	3	4	1	4	2	1
4	4	4	6	4	2	5	6	6	5	5	5	6	6	6	6	6	6	3	3	4	2	2	2	2	2	2	4	4	3	4	2	2	3	2	4	1	5	2	3
2	2	4	6	5	5	6	6	6	5	5	5	6	6	6	6	6	6	3	2	3	2	2	2	2	2	3	2	1	5	1	2	2	3	1	2	1	4	3	2
2	2	2	6	3	3	4	5	6	5	5	5	6	6	5	6	6	6	2	2	3	2	2	2	2	3	3	3	2	4	2	2	2	3	4	3	3	3	2	3
1	1	1	2	2	4	2	3	3	4	4	3	3	4	4	2	3	3	2	2	2	2	2	2	3	2	3	2	3	4	2	2	3	2	3	2	3	3	4	3
2	2	2	6	2	5	6	6	6	6	6	6	6	6	6	6	6	6	2	3	2	2	2	2	2	2	3	2	2	2	2	1	3	3	1	1	2	4	3	1
2	2	3	6	6	6	6	6	6	6	5	5	6	6	6	6	6	6	3	3	2	3	4	4	2	3	5	4	3	4	3	3	3	3	1	4	1	5	2	3
3	1	1	6	5	4	6	6	6	6	5	5	5	6	6	6	6	6	3	2	2	2	2	2	2	3	4	3	3	4	3	4	3	5	4	5	3	3	2	2
2	1	2	6	6	6	6	6	6	6	6	5	6	6	6	6	6	4	3	3	3	5	5	4	2	3	3	3	4	3	3	3	2	4	2	2	2	3	3	3
1	1	1	6	6	6	6	6	6	6	6	6	6	6	6	6	6	6	1	1	2	2	2	2	1	1	2	2	2	3	1	1	2	4	1	3	1	3	1	1
3	1	2																4	3	2	3	3	3	2	2	3	2	3	3	3	3	3	4	3	3	2	2	3	2
6	6	6	6	6	6	6	6	6	6	6	6	6	6	6	6	6	6	1	6	6	6	6	6	6	5	5	5	5	5	5	5	5	5	5	5	5	5	5	5
3	3	3	6	5	6	6	6	6	6	6	6	6	6	6	6	6	6	2	2	2	3	2	2	2	3	4	4	4	3	3	1	2	3	4	2	1	4	1	1
2	3	2	5	4	3	3	5	6	6	6	4	2	6	3	3	4	1	2	1	3	1	3	1	6	1	1	3	4	2	3	1	2	4	2	1	3	4	3	2
3	2	5	6	6	6	6	6	6	6	6	6	6	6	6	6	6	6	5	4	4	4	3	3	4	2	3	3	3	4	3	3	1	4	2	3	2	4	2	1
3	2	2	6	6	6	6	6	6	6	6	6	6	6	6	6	6	6	3	5	5	3	4	4	4	2	4	3	4	2	3	2	3	3	4	4	2	2	1	2
2	1	1	6	5	5	5	6	6	6	5	5	6	6	5	6	6	6	2	1	1	1	1	1	1	4	4	5	5	3	2	2	2	3	5	4	2	3	2	1
2	2	1	5	6	6	6	6	6	6	6	6	5	6	6	6	6	5	2	2	2	2	3	2	2	3	3	2	2	4	2	3	3	3	4	4	2	4	1	1
3	2	2	6	6	5	6	6	6	6	6	6	5	6	6	5	6	6	2	2	2	1	2	2	1	4	4	3	3	4	5	2	3	2	5	2	2	3	2	1
5	3	5	6	6	3	6	6	6	6	6	6	6	6	6	6	6	6	4	4	3	5	5	5	5	3	3	3	4	3	3	3	3	3	4	3	3	3	3	3
3	3	3	6	6	5	6	6	6	6	6	6	6	6	6	6	6	6	2	3	2	3	3	3	3	5	3	2	4	4	4	2	2	2	2	2	3	4	3	2
3	4	3	3	4	3	4	5	3	5	3	3	5	3	3	4	5	5	3	2	2	2	4	2	2	3	2	3	2	3	3	3	3	3	4	3	4	3	3	2
1	2	2	5	5	6	5	5	6	5	5	4	5	5	6	6	6	6	2	2	2	2	2	2	3	3	2	4	3	2	3	2	2	4	3	4	2	3	2	1
1	1	1	6	4	5	6	6	6	6	6	5	6	6	6	6	6	6	3	3	1	2	1	2	2	3	2	4	2	2	2	3	2	4	2	2	1	3	3	3
2	1	2	6	6	5	5	5	6	5	5	5	6	6	6	6	5	6	2	2	2	2	2	2	2	3	4	2	3	4	4	2	3	2	4	4	2	3	3	1
4	4	4	3	3	3	3	3	3	3	3	3	3	3	3	3	3	3	4	4	4	4	4	4	4	3	2	3	2	3	2	3	2	3	2	4	2	2	2	2
1	1	2	6	6	6	6	6	6	6	6	6	6	6	6	6	6	6	1	1	1	1	1	1	2	5	5	4	5	5	5	5	1	2	4	3	1	4	3	2
3	2	3	6	6	5	5	6	6	6	6	6	6	6	6	6	6	6	3	2	2	3	4	3	2	2	1	3	1	3	1	1	4	4	1	1	1	2	1	1
1	1	1	6	6	5	6	6	6	6	6	6	6	6	5	6	6	6	2	2	2	2	4	2	2	2	4	4	4	5	5	5	5	2	5	5	4	5	5	3
1	1	1	6	6	6	6	6	6	6	6	5	5	6	6	6	6	6	3	2	2	1	1	1	1	2	3	3	2	2	3	3	2	3	1	3	1	3	3	2
3	3	3	6	6	6	6	6	6	6	6	6	6	6	6	6	6	6	3	3	3	3	3	3	3	3	3	3	3	3	3	3	3	3	3	3	3	3	3	3
3	2	3	6	6	6	6	6	6	6	5	5	6	6	5	6	6	5	4	2	2	2	2	3	3	3	3	3	3	2	2	2	4	3	2	1	2	2	1	2
2	2	2	6	4	4	6	6	6	6	6	6	6	6	6	6	6	6	2	2	3	2	1	2	3	3	3	3	4	4	2	2	3	4	4	3	2	3	2	2

4	3	4	6	3	2	6	5	5	6	4	6	5	6	4	6	6	4	2	2	5	3	3	3	3	4	4	3	5	5	5	2	5	2	4	3	4	3	3	3
2	1	1	6	6	6	6	6	6	6	6	6	6	6	6	6	6	6	2	2	2	1	2	1	2	1	2	2	2	2	5	2	1	1	1	3	2	3	2	4
2	1	2	4	6	3	6	6	6	6	6	5	4	6	6	6	6	6	2	2	2	2	3	2	2	4	5	4	4	5	5	4	5	3	4	4	3	5	3	3
1	2	2	5	6	5	6	6	6	6	6	3	6	6	3	6	6	6	3	2	2	1	2	2	2	5	5	5	5	5	5	3	5	2	5	5	4	5	2	1
2	2	2	6	5	6	6	6	6	5	5	6	6	6	6	6	6	6	3	3	2	2	2	2	3	3	5	4	4	4	3	3	2	4	2	3	2	3	2	3
1	1	2	6	1	1	6	5	6	6	6	6	6	6	5	6	6	6	3	2	1	2	2	2	3	4	4	4	4	3	4	4	4	3	4	4	2	4	2	2
2	3	3	6	6	2	6	6	6	6	3	6	6	6	6	6	6	6	2	2	4	2	2	3	3	1	3	2	3	4	3	3	4	3	3	4	2	4	3	1
2	1	4	6	6	5	5	6	6	6	6	5	6	6	6	6	6	6	6	3	2	3	2	2	2	4	5	5	5	4	4	4	4	2	4	1	4	4	1	1
2	2	2	6	6	2	6	3	3	6	6	6	6	6	6	6	6	6	3	3	2	2	3	2	2	3	4	4	4	4	5	3	3	4	5	2	1	3	3	4
6	6	6	4	4	4	4	4	4	4	4	4	4	4	4	4	4	4	6	6	6	6	6	6	6	5	5	5	5	5	5	5	5	5	5	5	5	5	5	5
1	1	3	6	6	3	6	6	6	6	6	6	6	6	6	5	6	6	3	2	2	3	3	2	2	2	3	2	1	3	2	2	2	4	1	2	2	3	3	3
1	1	4	6	5	6	6	6	6	6	6	5	6	6	6	6	6	6	2	2	2	2	2	2	3	3	3	3	1	4	2	3	2	4	1	4	2	4	3	2
1	1	1	6	6	5	6	6	6	6	6	5	5	6	6	5	6	6	2	3	3	2	3	3	2	2	3	2	2	4	1	2	2	4	1	3	2	3	2	2
2	1	2	5	6	6	6	6	6	5	6	6	6	6	5	6	6	5	2	2	2	2	2	2	2	2	2	2	1	2	2	1	1	5	1	3	1	2	1	2
1	1	1	6	5	5	6	6	6	6	5	6	5	6	6	6	6	5	4	2	2	2	2	2	2	4	4	4	4	5	5	4	2	3	2	5	2	5	4	4
3	3	2	3	3	3	3	3	3	3	3	3	3	3	3	3	3	3	5	5	4	4	4	3	3	2	3	1	3	3	4	5	4	4	5	2	2	2	3	4
3	2	2	6	6	6	6	6	6	6	6	5	5	6	6	6	6	6	2	2	2	2	3	2	2	3	3	3	3	3	3	2	4	2	3	2	3	4	3	3
4	2	3	6	3	3	6	6	6	5	5	6	6	6	6	6	6	6	3	2	2	2	3	4	3	3	3	3	4	3	3	2	2	4	1	2	2	3	3	3
1	1	2	6	6	6	6	6	6	6	6	6	4	6	6	6	6	6	3	2	3	2	2	2	2	4	4	3	3	3	3	3	3	4	4	4	2	3	2	1
3	5	2	3	2	5	3	3	4	2	2	5	3	2	3	5	3	4	4	4	2	5	4	1	2	3	2	4	3	2	1	4	5	2	3	4	4	3	2	1
5	3	5	6	6	3	6	6	6	6	6	6	6	6	6	6	6	6	3	3	4	5	4	3	3	3	4	2	4	3	4	3	2	3	4	4	2	4	3	2
3	3	2	6	4	3	5	6	6	6	4	6	6	6	6	6	6	6	2	3	2	2	2	2	2	4	4	3	3	4	4	3	4	3	2	3	2	3	2	1
4	3	4	6	3	3	6	6	6	6	6	2	2	6	3	6	6	6	3	3	4	3	4	3	3	4	4	4	5	5	3	3	4	3	4	4	2	3	4	5
3	2	4	6	5	6	5	5	6	6	5	6	6	6	6	6	6	6	5	4	4	4	4	3	3	4	4	4	4	4	4	4	4	1	2	5	4	5	3	4
2	1	2	6	6	6	6	6	6	3	3	6	6	6	3	6	6	6	3	3	2	2	2	2	3	1	3	2	1	4	2	1	2	4	2	1	1	4	1	4
1	2	3	6	6	6	6	6	6	6	6	6	5	6	6	6	6	4	3	2	1	1	2	2	2	5	5	4	3	5	3	3	4	3	3	3	2	3	5	3
3	2	3	6	5	5	5	6	6	6	5	6	5	6	5	6	6	6	3	3	3	3	3	3	3	3	3	3	2	3	3	3	3	3	3	3	2	3	2	2
3	2	3	6	5	6	6	6	6	6	6	5	6	6	6	6	6	6	4	3	3	4	4	3	3	4	2	3	3	4	3	2	2	3	3	2	1	3	2	3
1	1	1	6	6	1	1	4	5	6	5	5	6	6	6	6	6	5	1	1	1	1	1	1	1	4	3	3	3	2	2	2	1	5	5	2	1	2	2	1
2	2	1	6	2	6	6	3	6	4	5	4	4	6	6	5	6	6	2	2	1	2	3	2	3	4	4	4	3	4	2	4	4	3	2	4	3	5	3	2
2	1	2	6	2	4	5	5	6	5	3	3	6	6	6	6	6	6	2	2	2	2	2	2	2	4	4	4	5	3	2	3	2	2	2	2	2	2	2	2
2	2	2	6	2	6	6	5	6	5	5	6	6	6	6	6	6	6	4	3	2	2	5	5	3	4	4	4	4	3	3	3	3	4	4	5	4	4	2	3
2	2	3	6	5	5	6	6	6	6	6	6	6	6	6	6	6	5	3	3	2	3	2	2	2	2	3	3	2	4	3	2	2	4	2	4	2	3	3	4
2	1	2	6	6	6	6	6	6	6	6	6	6	6	6	6	6	6	2	2	2	2	2	2	3	2	3	3	2	2	2	2	1	4	2	3	1	3	2	4
2	2	2	6	6	5	6	6	6	6	6	5	4	6	6	6	6	6	3	2	2	2	3	2	2	3	3	3	2	4	3	1	2	4	4	4	2	3	1	1
2	2	2	6	5	5	6	6	6	6	6	4	5	6	6	6	6	6	3	2	2	2	2	2	2	2	3	2	2	3	3	3	1	4	3	3	2	3	4	3
3	4	3	3	3	4	3	2	2	3	5	5	2	2	3	3	2	2	1	2	2	2	1	3	3	2	3	3	3	2	4	5	5	4	3	2	2	3	3	2
2	1	2	6	6	6	6	6	6	6	6	5	6	6	6	6	6	6	2	2	2	2	1	1	2	2	2	2	1	2	2	1	2	4	2	1	1	2	1	1
2	1	1	6	6	5	6	6	6	6	5	6	6	6	6	6	6	6	1	1	1	2	3	2	1	2	2	3	3	3	2	3	3	4	3	2	2	3	3	2
3	2	2	4	3	2	2	4	4	3	3	2	4	3	3	3	4	5	3	3	3	4	4	5	5	3	3	3	3	4	3	2	2	3	4	4	3	4	3	3
2	2	1	6	5	3	3	6	6	4	5	5	6	6	5	6	6	6	2	3	2	3	2	2	2	2	3	2	1	2	2	1	3	4	3	3	2	2	2	1
1	1	2	6	6	6	6	6	6	6	6	6	6	6	6	6	6	6	3	2	2	3	3	2	2	1	2	2	2	3	2	1	1	4	3	2	1	2	1	2
2	2	1	6	6	6	6	6	6	6	6	6	6	6	6	6	6	6	3	2	2	2	2	2	2	3	3	3	3	2	2	2	3	3	3	5	1	4	2	1
2	2	2	6	6	6	6	6	6	6	6	6	6	6	6	6	6	6	3	2	2	2	2	2	3	3	3	3	2	5	3	3	3	4	3	2	3	3	3	4
2	2	2	5	6	5	5	6	6	6	3	6	6	6	6	6	6	5	2	2	2	1	2	2	2	3	2	2	3	2	2	4	3	3	2	3	2	3	2	1
1	1	1	6	6	6	6	6	6	6	6	6	6	6	6	6	6	6	3	1	1	1	1	1	1	3	3	3	2	2	2	2	1	4	2	2	1	2	3	1
2	1	2	6	5	5	5	6	6	6	5	6	5	6	6	6	6	6	3	2	2	2	3	3	2	3	3	4	4	4	4	3	3	3	2	2	1	3	2	1
4	4	5	6	6	4	5	6	6	4	4	6	5	6	6	6	6	6	4	4	4	4	4	4	4	5	5	5	5	5	5	5	5	2	4	1	1	5	2	3
2	2	2	6	6	5	6	6	6	6	6	6	6	6	6	6	6	6	2	2	2	2	1	2	2	3	3	2	3	3	1	3	4	3	3	1	2	3	3	1
1	1	1	3	2	2	1	1	1	2	2	1	1	1	1	6	1	1	1	1	1	1	1	1	1	3	3	3	3	3	1	1	1	1	1	1	1	1	1	1
4	3	3	3	3	4	5	4	4	3	3	3	4	4	3	3	2	4	1	4	2	5	5	4	5	2	2	2	5	5	5	4	4	3	3	2	2	3	4	3
6	2	3	6	6	5	6	6	6	6	6	6	6	6	5	6	6	6	4	2	2	2	2	2	2	5	5	1	5	5	5	5	5	1	1	3	1	4	3	3
2	2	2	6	6	3	6	6	6	6	5	4	3	6	3	3	6	6	4	2	2	3	2	2	2	3	4	4	5	5	3	2	4	2	2	3	2	3	1	1
3	2	4	6	6	6	6	6	6	6	6	6	6	6	6	6	6	6	4	3	4	3	4	3	5	3	3	4	3	5	4	3	4	4	4	4	3	4	4	3
2	2	2	6	4	4	6	6	6	6	6	6	6	6	6	6	6	6	2	3	3	2	3	2	3	3	3	3	3	3	3	4	3	4	3	3	2	3	3	3
2	2	2	6	6	6	6	6	6	6	6	6	6	6	6	6	6	6	3	2	2	2	2	2	2	2	2	2	2	3	2	2	2	4	2	4	2	3	3	2
2	2	2	6	6	5	6	6	6	6	6	6	6	6	6	6	6	6	2	2	2	2	2	2	2	3	4	3	4	4	3	2	3	3	4	4	2	5	3	2
3	3	4	6	5	5	5	6	6	6	6	6	6	6	6	6	6	6	5	4	4	4	5	6	4	3	3	3	2	4	3	3	2	3	3	3	2	3	3	1
2	2	3	6	6	3	6	6	6	6	6	4	5	6	6	6	6	6	3	2	2	2	3	2	2	3	4	3	4	4	3	1	2	4	3	3	2	3	3	1
2	3	5	6	6	6	6	6	6	6	6	6	6	6	6	6	6	6	4	2	3	3	4	3	2	2	2	3	2	3	1	1	1	4	2	3	3	4	2	2
3	1	2	6	6	6	6	6	6	6	5	6	6	6	6	6	6	6	3	2	2	2	1	2	3	3	3	4	2	3	3	1	2	4	1	3	2	3	1	2
1	2	3	6	6	6	6	6	6	6	6	6	6	6	6	6	6	6	2	2	2	2	1	2	2	3	3	3	4	4	5	2	2	3	3	2	3	3	3	2
3	2	5	6	3	2	3	5	6	3	3	5	6	6	4	6	6	6	3	2	2	3	2	2	2	2	3	3	3	3	4	2	1	3	5	4	4	3	2	4
4	3	4	6	2	4	6	6	6	6	6	6	6	6	6	6	6	5	2	3	4	4	2	3	3	3	3	2	3	4	3	2	2	3	2	1	3	5	4	5
2	2	2	6	6	6	6	3	6	6	6	4	6	6	6	6	6	6	3	2	2	2	2	2	2	2	3	3	3	3	2	1	1	4	1	3	1	3	1	1
2	2	3	6	6	6	6	6	6	6	6	6	6	6	5	6	6	6	3	3	5	5	4	3	2	2	2	2	5	4	4	1	4	2	3	1	1	3	2	2
3	3	3	3	2	2	4	6	6	6	6	6	6	6	6	6	6	5	2	3	3	4	3	2	2	2	3	4	2	2	2	3	3	2	3	3	1	2	2	3
2	2	4	6	4	4	5	6	6	5	6	4	5	6	6	6	6	6	3	2	2	2	2	2	2	3	4	4	4	5	4	4	4	3	4	4	4	5	5	4
1	1	2	6	6	5	6	6	6	6	5	6	6	6	6	6	6	6	2	2	1	2	2	1	1	4	5	5	5	3	4	4	4	2	5	3	2	4	1	1
2	2	2	6	5	5	4	6	6	6	6	5	4	6	5	6	6	5	2	2	2	2	2	2	2	3	4	3	3	3	2	2	3	4	3	5	2	4	2	1
3	3	3	3	3	3	3	3	3	3	3	3	3	3	3	3	3	3	3	3	3	3	3	3	3	4	4	4	4	3	3	3	3	4	3	3	3	3	3	3
2	1	2	6	5	6	6	6	6	6	6	5	6	6	6	6	6	6	2	3	3	3	4	3	3	3	4	4	2	4	4	4	2	5	3	2	3	4	4	1
2	2	1	6	4	3	6	5	5	6	5	5	4	6	6	4	6	6	1	1	1	2	2	2	2	2	2	2	3	3	2	2	2	4	3	2	2	3	1	1
2	2	2	6	6	6	6	6	6	6	6	6	5	6	3	6	6	6	4	2	3	2	2	2	2	3	3	1	2	4	4	3	3	2	1	2	2	3	1	1
3	3	4	6	6	6	6	6	6	6	6	6	6	6	6	6	6	6	4	3	3	3	3	3	4	3	4	4	5	3	3	2	2	3	4	4	4	5	3	3
1	1	1	6	5	6	6	6	6	6	6	6	6	6	6	6	6	6	2	2	2	2	2	2	5	3	3	3	2	2	2	2	2	4	3	4	1	3	1	1
4	2	2	6	4	5	6	6	6	6	6	3	6	6	6	6	6	5	2	2	2	2	2	1	3	3	3	3	2	4	3	2	3	4	2	3	3	4	3	3
2	2	3	6	5	3	6	6	6	5	5	6	6	6	6	6	5	6	2	2	3	3	3	3	2	3	4	1	3	3	3	2	2	3	4	3	2	5	3	1
2	2	5	6	4	5	5	5	6	5	5	6	6	6	6	6	6	6	3	4	3	3	3	3	3	2	3	4	3	2	2	2	1	4	2	5	1	5	2	3
2	2	2	6	3	4	3	4	4	5	5	6	6	6	6	6	6	6	2	3	3	4	3	3	3	3	3	2	2	3	2	2	1	4	3	2	1	1	1	1
1	1	1	6	5	5	5	6	6	6	6	6	6	6	6	6	6	6	2	1	1	1	1	1	1	3	3	3	3	3	2	2	3	4	3	3	2	3	1	1
1	1	6	1	3	4	5	6	5	4	3	2	1	2	3	4	5	6	6	4	5	3	2	1	2	4	3	2	1	2	3	4	5	4	3	2	1	2	3	4
2	2	2	6	2	2	3	4	5	3	3	2	5	6	3	6	6	5	2	2	2	2	2	2	2	1	3	3	3	3	3	2	1	4	4	3	2	3	3	1
4	3	3	5	4	2	6	6	6	6	6	3	5	6	6	6	6	3	2	1	2	3	3	3	2	2	2	3	4	5	3	3	2	3	4	2	2	3	3	2
2	2	2	6	1	3	6	6	6	6	6	6	6	6	6	6	6	6	2	2	2	1	2	1	1	2	3	3	2	3	2	2	2	4	2	3	1	3	2	2
1	1	1	6	5	3	6	6	5	6	5	4	6	6	6	6	6	6	1	1	1	1	1	1	1	2	2	2	1	2	1	2	1	5	2	1	1	1	1	1
2	1	2	6	6	3	6	5	6	6	6	6	6	6	6	6	6	6	5	3	3	3	3	2	3	4	4	4	4	4	4	3	4	3	2	2	2	5	3	3
2	5	1	6	3	3	4	3	3	3	3	3	5	6	5	5	6	6	2	2	2	2	1	2	2	4	4	4	5	4	4	4	4	3	3	3	3	3	3	3
2	2	3	6	5	3	6	6	6	6	6	3	6	6	6	6	6	6	2	2	2	2	2	2	2	4	4	4	3	3	3	3	2	4	2	4	2	5	3	2
2	2	2	6	6	5	6	6	6	6	6	6	6	6	6	6	6	6	2	2	2	2	2	2	2	2	2	3	3	3	2	3	2	4	2	3	2	3	3	3
4	2	3	5	6	5	6	6	6	6	6	6	5	6	5	6	6	6	1	3	2	2	2	2	2	3	3	3	3	3	3	2	2	4	3	3	2	3	2	2
3	2	3	6	6	5	6	6	6	6	5	6	5	6	6	5	6	6	2	2	2	3	2	2	2	3	3	3	4	3	2	3	2	4	1	3	2	3	2	4
2	2	2	6	5	5	5	5	5	3	3	2	6	6	3	6	6	6	2	2	2	2	2	2	2	1	3	3	1	3	1	1	1	4	2	2	1	2	1	1
3	2	2	6	3	6	5	6	6	6	5	5	6	6	6	6	6	6	3	2	2	2	2	2	2	3	4	3	3	4	3	2	4	4	4	3	2	4	3	3
5	3	5	6	3	3	6	6	6	6	6	5	6	6	6	6	6	3	2	2	2	3	2	3	2	5	5	5	4	4	5	4	5	2	3	3	2	3	4	1
1	2	2	6	6	6	6	6	6	6	6	6	5	6	6	6	6	6	4	3	3	3	3	3	3	2	3	2	3	3	3	1	1	2	2	2	1	3	3	2
1	3	3	6	6	6	6	6	6	6	6	6	6	6	6	6	6	6	6	1	1	1	1	1	1	3	3	3	3	3	3	3	3	3	3	3	3	3	3	3
4	3	4	6	6	5	6	6	6	6	6	6	6	6	6	6	6	6	4	3	3	5	6	5	3	3	2	3	2	4	3	5	1	4	3	2	1	3	4	2
5	4	5	6	6	5	6	6	6	6	6	6	6	6	6	6	6	6	4	3	3	3	2	3	2	3	3	3	3	1	4	1	4	2	4	2	1	3	1	1
3	1	1	6	6	2	6	6	6	5	5	6	6	6	5	6	6	6	2	2	1	1	1	1	2	4	4	4	4	4	4	3	4	2	3	1	2	4	2	2
1	1	1	6	6	6	6	6	6	6	6	6	6	6	6	6	6	6	1	1	1	1	1	1	1	1	3	1	1	3	1	3	1	5	2	2	1	1	2	2
2	2	3	6	6	2	6	5	5	6	5	3	6	6	6	6	6	6	3	3	2	3	3	3	3	2	1	3	3	2	2	2	2	4	2	5	2	3	2	1
2	2	2	6	2	6	5	6	6	6	6	5	6	6	6	6	6	6	2	2	5	2	2	2	2	4	4	4	4	4	4	4	4	4	4	4	4	4	4	2
1	2	3	6	3	3	5	6	6	6	5	6	6	6	6	6	6	6	4	5	3	4	3	3	3	2	3	3	3	5	3	4	4	3	4	4	3	5	5	2
1	1	1	6	6	2	6	4	6	3	3	3	6	6	6	6	6	6	2	2	2	2	2	2	2	3	5	4	4	4	4	2	4	3	2	4	3	5	3	2
2	2	2	6	5	3	6	6	6	6	6	3	6	6	6	6	6	6	2	2	2	2	2	2	2	3	3	3	4	4	4	4	4	4	4	4	4	4	4	4
1	1	1	6	4	3	6	6	5	6	6	6	6	6	6	6	6	6	2	1	2	1	1	1	1	2	4	1	1	2	1	2	1	5	1	1	1	2	1	1
2	2	2	6	6	6	6	6	6	6	6	6	6	6	6	6	6	6	2	3	2	2	2	2	2	3	2	2	2	3	2	1	2	4	1	1	1	3	3	1
1	2	2	6	5	2	6	6	6	6	5	6	6	6	6	6	6	6	3	3	2	2	3	2	2	3	3	3	3	2	1	2	2	4	1	3	1	2	3	2
3	2	3	6	6	5	6	6	6	6	6	5	6	6	6	6	6	6	3	3	3	4	4	3	3	2	3	3	2	3	3	2	2	4	2	2	2	2	2	1
2	2	4	6	6	6	6	6	6	6	4	6	6	6	6	6	6	6	4	3	4	3	5	3	3	3	4	4	4	5	5	4	4	4	4	4	3	5	4	4
1	3	2	6	6	6	6	6	6	5	5	6	6	6	6	6	6	6	3	2	2	3	2	2	2	1	2	3	3	3	2	1	1	4	2	3	3	4	2	5
1	1	1	6	5	3	5	5	6	6	6	5	5	6	6	6	6	6	2	2	2	2	3	2	2	4	3	3	3	3	2	1	2	4	2	3	2	2	2	1
1	5	1	6	2	5	3	5	6	6	6	6	5	6	6	6	6	6	3	2	1	1	2	2	2	4	4	4	4	3	4	3	4	3	3	4	4	4	3	4
2	1	2	6	3	6	5	6	6	6	5	5	5	6	6	6	6	6	2	2	2	2	2	3	3	3	3	2	3	4	4	2	3	4	3	2	3	2	2	1
3	4	2	4	4	2	3	4	4	4	5	4	4	5	5	4	3	4	5	4	5	5	3	3	5	4	3	4	3	3	4	3	4	3	4	3	4	3	3	3
1	1	1	6	1	1	6	6	6	6	6	5	6	6	6	6	6	6	3	6	5	6	2	4	2	3	4	4	4	5	5	4	3	3	5	5	4	5	4	3
6	4	6	6	6	6	6	6	6	6	6	5	5	6	6	6	6	5	6	6	5	5	6	5	1	2	2	3	3	3	4	4	3	4	2	5	2	5	3	4
5	4	1	4	3	5	6	4	3	4	5	4	4	6	5	6	6	5	1	1	1	2	2	3	2	2	3	2	3	4	3	2	3	3	3	4	2	3	2	2
2	2	3	6	5	3	4	5	3	3	3	3	6	6	6	6	6	6	5	3	2	3	4	3	3	3	3	4	3	4	3	3	3	2	3	4	2	4	3	1
3	1	3	6	6	6	6	6	6	6	6	5	6	6	6	6	6	6	3	2	3	2	2	2	2	4	3	2	2	4	4	5	1	3	2	1	3	2	3	2
2	2	3	6	6	5	6	6	6	6	5	6	5	6	6	6	6	6	4	2	2	2	4	2	2	3	3	3	2	3	2	1	2	4	3	4	2	4	2	4
2	1	1	6	3	1	2	3	3	4	3	5	3	4	3	6	6	6	2	2	2	2	1	1	1	4	5	4	4	3	5	4	4	2	5	1	4	3	3	2

2	3	2	6	2	2	2	2	2	2	2	6	6	6	6	6	6	6	2	1	1	1	2	1	1	4	4	4	4	4	4	4	2	5	4	4	4	4	4	2
2	3	2	6	6	3	5	6	6	6	6	3	3	6	3	4	6	3	2	2	1	2	2	2	2	4	4	4	4	3	4	3	2	2	4	5	3	5	2	1
2	2	2	6	6	5	6	6	6	6	5	6	6	6	6	6	6	5	2	2	2	2	2	2	2	3	4	4	4	4	4	3	3	3	4	3	3	3	3	1
2	4	2	6	5	3	6	6	6	5	6	6	6	6	6	6	6	6	2	2	2	2	2	2	2	3	3	3	3	3	3	2	2	4	3	2	2	3	2	1
3	5	5	6	6	6	6	6	6	6	6	6	6	6	6	6	6	6	5	2	5	4	5	5	3	5	5	5	5	5	5	5	5	1	5	5	5	5	5	5
1	1	1	6	3	2	3	3	6	6	5	3	6	6	6	6	6	6	1	1	1	1	1	1	1	3	5	4	5	5	4	3	2	4	4	4	2	3	3	2
3	1	2	6	5	3	5	6	6	6	5	5	6	6	6	6	6	1	2	2	3	2	3	3	3	4	4	4	4	4	5	3	3	2	4	5	3	3	4	2
3	3	3	6	5	4	5	5	6	6	5	5	6	6	6	6	6	6	5	4	4	4	4	3	3	2	3	3	2	2	3	3	2	4	2	2	2	2	2	3
6	3	6	6	1	1	6	1	1	6	1	1	1	6	1	1	1	6	6	5	6	4	6	6	6	5	5	5	5	5	5	5	5	5	5	5	5	5	5	5
2	2	2	6	6	4	6	6	6	6	6	6	5	6	6	6	6	3	2	2	2	3	2	3	2	2	3	3	2	3	2	2	3	4	2	2	2	3	3	1
2	2	3	6	6	5	5	3	3	6	3	6	6	6	6	6	6	6	2	2	2	3	3	3	3	4	4	4	4	4	4	3	4	2	5	3	4	4	4	3
1	1	2	6	6	2	6	6	6	6	6	6	6	6	6	6	6	6	2	2	2	2	3	2	2	2	4	1	3	3	3	2	3	5	3	3	2	3	3	2
3	2	2	6	6	3	6	6	6	6	6	6	6	6	6	6	6	6	4	2	2	3	2	2	2	3	4	4	2	3	2	1	2	4	3	2	1	3	1	1
2	2	3	6	5	4	5	6	6	6	5	3	3	6	6	6	6	3	4	2	2	2	2	2	2	4	4	4	4	4	4	4	4	4	4	4	4	4	4	4
1	1	1	6	6	3	6	6	6	6	6	6	5	6	6	6	6	6	1	1	1	1	1	1	1	2	1	1	2	2	2	1	1	4	3	1	1	1	1	1
2	2	2	6	6	6	6	6	6	6	6	6	6	6	6	6	6	6	2	3	3	2	2	2	2	1	2	1	1	3	1	2	1	5	1	3	1	2	2	4

APSI_II16	APSI_II17	APSI_II18	APSI_III19	APSI_III20	APSI_III21	APSI_III22	APSI_III23	SC0	SozialE6	SozialE5	sozialeE1	SozialeE2	SozialeE3	SozialeE4	SozialeE3x	APSI_P9x	SW1	SW2	SW3	SW4	SW5	SW6	SW7	SozialeE4x	socialdesirable	GBJW	Lifesatifaction	PBJW	KG	DG	cheating	selfefficacy	procrastination	PQ	NQ
5	5	5	5	5	5	5	5		2	5	5	5	5	5	2	1	5	5	5	5	5	5	5	2	3,50	5	5	5	3	3,00	3	5	3	5	2
5	5	5	5	5	5	5	5		1	6	6	6	6	6	1	1	6	6	6	6	6	6	6	1	3,50	6	6	6	3	2,67		5	5	6	1
5	5	5	5	5	5	5	5		1	6	6	6	6	6	1	1	6	6	6	6	6	6	6	1	3,50	5	6	6	3	2,67	4	5	4	6	1
2	2	2	2	3	3	2	2	1	4	2	2	3	4	6	3	4	1	1	3	4	5	3	2	1	2,50	4	2	3	2	2,44	3	3	3	2	3
2	2	1	1	1	1	2	1	1	2	2	3	5	5	6	2	4	2	1	4	4	4	5	2	1	2,50	4	2	2	1	1,56	6	3	3	3	2
5	4	4	3	1	2	2	1	1	4	2	3	3	3	3	4	5	2	2	3	2	5	6	3	4	3,33	5	2	2	1	1,11	6	4	2	3	4
3	4	2	5	4	2	4	4	1	3	2	5	5	4	2	3		2	1	2	1	4	4	5	5	3,83	4	3	3	4	3,78	4	3		4	4
1	1	2	1	1	1	1	1	1	3	2	1	2	5	3	2	2	1	2	2	4	3	4	2	4	2,33	3	2	2	2	1,44	6	2	2	2	3
2	3	3	3	4	3	3	2	1	4	2	2	3	3	5	4	1	2	1	5	4	4	4	2	2	2,83	4	3	4	2	2,33	6	3	3	2	3
3	3	3	3	2	3	2	2		2	2	3	2	3	3	4		2	3	4	4	4	3	4	4	2,83	4	3	3	3	3,33	5	3		2	3
4	4	4	3	2	3	3	3		1	1	1	1	5	6	2	4	2	3	5	2	2	5	4	1	1,17	6	4	5	3	4,00	6	4	4	1	1
2	3	2	1	2	2	1	1	1	1	2	2	3	6	6	1		2	2	3	3	4	6	5	1	1,67	3	1	2	1	1,11	6	4		2	1
2	3	3	2	2	2	3	3	1	6	3	3	3	3	3	4		2	2	1	3	2	3	2	4	3,83	3	2	2	2	2,44	6	2		3	5
2	2	2	1	3	2	2	1	1	1	2	3	3	4	4	3	2	3	2	3	3	4	5	5	3	2,50	4	4	3	3	2,78	6	4	2	3	2
1	1	1	1	1	1	1	1	1	6	1	1	1	2	5	5	1	1	1	1	4	1	1	1	2	2,67	2	2	1	3	2,33	6	1	1	1	4
1	1	1	1	1	2	4	2	1	4	1	2	3	4	6	3	2	1	1	1	6	1	1	1	1	2,33	5	1	1	2	1,22	6	1	2	2	3
4	3	4	3	4	4	3	2	1	2	2	3	4	4	4	3	2	1	1	2	4	4	4	3	3	2,83	3	2	2	2	2,22	5	3	3	3	3
3	4	3	3	1	2	2	1	1	1	2	3	4	5	5	2	2	2	2	3	2	5	5	4	2	2,33	4	2	3	3	2,22	6	4	2	3	2
3	3	3	2	3	3	2	2	1	1	2	2	3	5	4	2		2	2	3	3	3	2	3	3	2,17	3	2	2	2	2,11	6	3		2	2
4	4	2	3	5	3	3	3	1	3	1	1	2	6	5	1	2	2	3	2	3	3	2	2	2	1,67	3	2	2	1	1,89	6	3	2	1	2
5	5	5	4	5	5	3	4	1	1	2	2	3	5	6	2		3	3	3	2	6	6	6	1	1,83	4	4	3	2	2,56	5	5		2	1
3	3	2	1	1	2	2	1	1	1	2	1	4	5	3	2	2	1	1	1	5	3	2	2	4	2,33	4	1	1	1	1,33	6	2	2	2	2
4	4	4	3	4	3	3	3	1	2	1	2	3	5	6	2	2	3	3	2	3	3	4	3	1	1,83	2	2	2	3	2,00	6	3	4	2	2
3	3	3	2	3	2	3	2	1	5	2	2	3	4	3	3	4	3	3	3	3	3	6	3	4	3,17	4	2	3	2	2,56	5	4	4	2	4
2	1	2	4	2	3	3	2		4	2	2	3	5	5	2		1	1	2	4	3	3	2	2	2,50	4	2	2	2	2,00	5	2		2	3
2	2	1	1	2	2	2	2		3	2	2	2	4	4	3	2	2	2	2	2	3	3	2	3	2,50	4	2	2	2	1,78	5	3	3	2	3
4	4	4	2	3	3	3	3	1	2	3	5	4	5	4	2	1	2	1	2	2	4	2	2	3	3,17	5	2	2	2	1,89	6	3	2	4	2
2	1	2	1	3	3	3	3		6	2	3	2	2	5	5	2	1	1	1	5	2	5	1	2	3,33	4	2	2	3	2,44	6	2	2	2	4
4	4	3	4	4	2	3	2	1	1	2	2	3	5	5	2		2	3	4	2	5	4	4	2	2,00	4	3	3	2	2,33	6	4		2	2
2	3	1	1	1	2	2	2		1	2	5	2	5	6	2	2	2	2	2	4	3	6	2	1	2,17	3	1	1	1	2,11	6	3	3	3	1
3	3	3	2	2	1	3	2		6	2	2	3	3	4	4	4	2	2	2	3	5	5	4	3	3,33	6	3	2	2	1,44	5	3	4	2	4
1	3	2	1	1	2	3	3		6	3	3	2	2	3	5	2	3	3	3	4	3	4	5	4	3,83	5	5	5	4	2,89	6	3	2	3	5
2	2	4	1	3	1	2	1	1	2	2	2	2	6	6	1	1	2	1	2	5	4	6	2	1	1,67	4	3	5	2	1,67	6	3	2	2	1
3	3	4	3	1	3	3	3		6	1	2	1	2	4	5	4	2	2	2	3	4	5	3	3	3,00	6	3	2	2	2,11	6	3	3	1	5
3	3	4	1	2	2	1	2	1	2	2	3	3	3	4	4	2	2	2	2	5	5	5	2	3	2,83	5	2	2	2	1,67	5	3	3	3	3
4	3	3	2	2	2	3	3	1	1	2	2	2	6	6	1	2	2	1	3	3	3	3	2	1	1,50	3	2	2	2	2,33	6	3	3	2	1
3	3	3	2	1	3	2	1	1	1	2	2	2	4	5	3	1	2	3	2	3	5	5	5	2	2,00	4	3	1	1	1,22	5	4	2	2	2
5	4	3	2	1	2	1	3	1	4	4	2	4	3	2	4		2	2	2	2	5	4	3	5	3,83	5	3	4	3	2,56	5	3		3	4
4	3	3	4	3	2	3	2		1	1	1	1	6	5	1	2	2	1	2	4	2	4	1	2	1,17	3	3	3	4	3,67	6	2	3	1	1
5	5	4	2	1	3	3	2	1	1	3	3	3	5	6	2		3	2	2	2	4	4	4	1	2,17	4	3	2	2	2,89	6	3		3	1
3	3	3	3	5	4	3	4	1	2	2	4	3	5	5	2	1	3	3	4	5	4	5	5	2	2,50	4	3	3	2	2,44	6	4	3	3	2
2	2	2	2	1	1	2	1		3	3	4	6	5	6	2	2	1	1	2	5	2	5	1	1	3,17	5	3	4	4	2,78	6	2	3	4	2
4	3	4	3	3	3	3	3		2	2	2	4	4	5	3	2	3	2	2	2	4	2	4	2	2,50	4	4	3	2	2,78	4	3	3	3	2
3	3	3	2	1	3	4	1	1	4	1	1	2	4	5	3	1	2	2	4	4	4	3	3	2	2,17	3	2	2	1	2,67	6	3	2	1	3
1	1	1	2	1	3	4	1	1	1	2	3	3	3	5	4		2	2	2	3	4	4	3	2	2,50	4	2	3	2	1,11	6	3		3	2
3	3	2	1	2	4	3	3	1	1	3	3	3	3	3	4	4	2	2	3	4	4	4	3	4	3,00	5	2	2	2	2,44	6	3	3	3	3
5	5	2	5	5	5	5	5	1	5	1	2	2	3	6	4		3	3	3	1	6	2	6	1	2,50	3	3	2	2	2,33	4	4		2	3
5	4	4	3	4	4	3	4	1	4	1	1	2	4	5	3	4	4	4	5	1	5	5	5	2	2,17	5	5	4	3	2,78	5	5	4	1	3
3	3	3	2	2	3	3	3	1	1	1	2	4	4	5	3	2	1	1	2	3	4	4	2	2	2,17	3	2	2	2	1,89	6	3	3	2	2
2	2	2	2	1	1	2	1	1	2	2	2	2	3	6	4		2	2	1	4	2	3	2	1	2,17	4	2	2	2	2,00	6	2		2	2
1	1	2	1	1	1	1	1		2	1	2	2	3	5	4	2	2	1	1	6	2	5	1	2	2,17	5	2	1	2	1,22	6	2	2	2	3
5	5	5	5	5	5	4	5	1	4	2	1	2	6	5	1	2	3	2	3	4	5	4	4	2	2,00	3	3	3	3	3,56	4	3	3	2	2
3	2	2	1	1	2	2	1	1	3	3	3	3	3	3	4	2	2	1	4	5	4	5	2	4	3,33	4	3	2	2	2,67	6	3	2	3	4
3	3	3	4	3	2	2	2	1	2	1	2	3	2	5	5		3	2	3	2	4	4	2	2	2,50	4	3	2	3	2,67	5	3		2	3
4	4	3	2	3	2	2	3	1	4	2	2	4	3	4	4	2	2	2	2	4	3	5	2	3	3,17	5	1	3	2	1,44	6	3	3	3	4
4	4	4	2	3	2	3	2	1	2	1	2	2	5	4	2	2	2	2	2	5	3	3	3	3	2,00	3	2	2	2	2,11	6	2	2	2	2
2	2	2	1	1	3	3	2		2	2	2	2	5	5	2		2	2	4	3	3	4	3	2	2,00	4	3	2	3	2,89	6	3		2	2
2	2	2	2	1	1	1	1	1	1	1	1	1	5	6	2	1	1	1	2	5	3	2	1	1	1,17	5	1	1	1	1,11	6	2	2	1	1
4	3	3	2	1	2	3	3	1	1	1	1	2	3	6	4	2	1	2	2	5	5	2	2	1	1,67	3	2	2	2	1,89	6	2	3	1	2
3	3	3	4	4	4	3	4	1	4	1	3	4	4	4	3	4	2	3	2	4	4	4	3	3	3,00	5	3	3	4	2,11	6	3	4	3	3
3	3	3	3	2	2	3	2	1	4	2	2	3	3	5	4		3	3	3	3	5	5	4	2	2,83	4	1	2	2	3,11	6	4		2	3
3	3	3	3	2	3	3	3	1	1	2	4	3	6	5	1	2	1	1	2	5	3	5	4	2	2,17	5	2	2	1	2,00	5	3	3	3	1
2	1	3	2	2	2	3	2		6	2	5	4	2	6	5		3	2	3	5	3	6	2	1	3,83	6	3	3	2	1,78	5	3		4	4
2	2	2	3	1	3	3	3	1	1	2	2	4	3	4	4	2	2	2	3	3	3	5	5	3	2,67	5	2	2	2	2,33	5	3	3	3	3
3	3	3	2	3	3	3	1	1	5	3	3	3	4	6	3	2	2	2	3	3	4	4	4	1	3,00	4	2	1	1	1,22	6	3	3	3	3
3	2	2	2	1	2	2	1	1	1	2	4	3	5	3	2	2	1	1	2	5	3	2	2	4	2,67	5	2	3	4	1,89	6	2	3	3	2
2	3	2	1	1	2	2	1		2	3	5	4	3	3	4		3	2	4	5	3	5	4	4	3,67	3	2	3	1	1,89	6	3		4	3
1	1	2	1	1	3	3	1		6	2	3	2	1	2	6	2	1	1	2	1	3	5	1	5	4,00	4	2	2	1	1,11	5	3	3	2	6
4	4	4	3	3	2	1	3	1	4	1	2	2	3	5	4	2	4	5	2	3	6	5	2	2	2,50	5	2	5	4	3,78	6	4	2	2	3
4	4	4	3	2	4	2	3	1	1	2	1	5	4	4	3	1	2	2	3	3	2	2	2	3	2,50	4	3	3	4	2,44	5	2	2	3	2
2	2	3	1	2	2	3	2	1	3	2	4	2	3	2	4		2	2	2	4	2	3	2	5	3,33	4	2	2	2	2,78	5	2		3	4
3	2	1	1	4	2	3	3	1	1	6	5	5	6	2	1	2	5	2	2	5	3	4	6	5	3,83	5	4	4	3	3,67	4	3	3	5	2
4	4	4	3	2	3	4	3	1	1	2	3	4	3	3	4	4	4	3	4	1	6	6	5	4	3,00	4	3	3	2	3,44	5	5	3	3	3
3	4	5	2	3	2	2	1	1	1	3	2	4	3	6	4		3	1	3	6	5	4	4	1	2,50	3	2	3	4	3,00	6	3		3	2
3	3	3	3	3	3	3	3	1	4	3	3	3	3	3	4		3	3	3	3	3	3	3	4	3,50	3	3	3	4	3,67	3	3		3	4
3	3	3	3	3	3	3	2	1	2	2	2	3	5	5	2	2	2	2	2	5	3	3	2	2	2,17	4	2	2	2	1,33	6	2	3	2	2
2	2	2	1	2	3	3	4	1	1	2	2	1	5	6	2	4	1	1	2	6	1	2	2	1	1,50	4	2	3	1	1,89	6	1	2	2	1
3	3	3	2	1	3	3	1		5	2	4	2	2	5	5	4	2	2	2	2	5	6	2	2	3,33	4	3	2	2	1,22	6	3	4	3	4
3	3	3	1	1	2	1	2	1	4	4	3	5	3	4	4	4	5	2	3	5	4	5	5	3	3,83	5	3	4	3	2,89	6	4	3	4	4
3	3	2	1	2	3	3	2	1	4	2	3	4	4	4	3		2	2	5	3	5	2	3	3	3,17	5	2	3	3	1,89	6	3		3	3
1	1	3	1	1	3	2	1	1	6	2	4	2	5	6	2	2	1	1	2	6	2	5	1	1	2,83	3	2	3	2	1,33	6	2	2	3	3
2	2	3	1	1	2	2	2	1	2	3	5	4	5	5	2		2	2	2	3	3	6	5	2	3,00	4	2	2	1	2,33	5	3		4	2
1	1	2	1	1	1	4	1		5	2	3	2	1	2	6	2	1	1	1	6	2	2	2	5	3,83	4	2	2	4	4,22	4	1	3	2	5
2	2	2	2	2	2	2	2	1	2	2	2	4	3	3	4	2	1	2	2	5	3	3	4	4	3,00	4	2	2	2	1,44	5	2	2	3	3
2	2	1	1	1	2	2	1	1	1	2	2	2	5	6	2	2	2	1	2	5	2	4	2	1	1,67	4	2	2	1	1,56	6	2	3	2	1
5	5	5	5	5	5	5	5		1	6	6	6	6	6	1	1	6	6	6	6	6	6	6	1	3,50	6	6	6	3	2,67	6	5	5	6	1
2	1	1	1	1	1	1	1	1	1	2	2	3	5	5	2	2	1	1	2	5	2	4	2	2	2,00	3	2	2	2	1,56	6	2	3	2	2
5	5	4	2	1	1	3	2	1	1	1	3	3	5	2	2		4	2	3	1	6	2	3	5	2,50	5	4	3	1	2,33	5	4		2	3
3	3	3	2	4	2	2	2	1	1	2	3	3	6	5	1		2	2	4	4	6	5	3	2	2,00	3	3	2	2	2,33	6	4		3	1
3	3	3	3	1	3	3	1	1	4	2	3	3	4	4	3		2	2	3	3	5	4	2	3	3,00	4	2	2	2	1,44	5	3		3	3
3	3	3	2	3	3	4	2	1	4	1	2	2	1	1	6	4	1	3	2	2	1	1	1	6	3,50	1	1	2	4	4,56	3	2	3	2	5
3	3	3	1	1	3	3	2	1	3	1	2	3	5	5	2		2	4	3	1	3	5	5	2	2,17	4	2	2	2	2,00	6	4		2	2
								1																											
								1																											
								1																											
								1																											
								1																											
4	3	3	1	1	1	2	1		2	2	3	3	5	4	2		3	2	3	2	3	5	3	3	2,50	4	3	3	3	2,33	6	3		3	2
								1																											
								1																											
3	4	3	3	3	3	3	2	1	2	2	4	2	5	6	2	1	3	3	4	2	3	4	3	1	2,17	4	2	2	1	1,33	6	4	3	3	2
								1																											
4	4	3	2	2	3	2	1	1	1	2	2	3	5	6	2	2	2	2	2	5	6	4	2	1	1,83	5	2	4	5	2,11	6	3	3	2	1
								1																											
3	2	1	1	3	1	1	1		4	2	2	2	5	2	2	2	2	1	1	5	2	1	1	5	2,83	5	1	2	1	1,56	6	1	2	2	4
								1																											
3	3	2	1	1	2	3	1	1	4	3	4	5	3	5	4	2	2	2	2	4	2	5	2	2	3,67	3	2	3	3	1,78		3	3	4	3
								1																											
5	5	5	5	5	5	5	5	1	1	1	1	1	6	6	1	1	6	6	6	1	6	6	6	1	1,00	4	5	5	6	6,00	6	6	5	1	1
3	2	1	1	1	1	4	2	1	1	2	3	4	3	5	4		2	2	1	3	4	5	3	2	2,67	6	3	2	1	2,11	6	3		3	2
3	3	2	3	1	3	3	3	1	2	3	3	2	2	4	5	2	2	1	2	5	4	5	2	3	3,00	3	3	2	2	2,33	4	3	2	3	3
3	3	3	3	3	3	2	2		3	2	2	3	6	5	1	2	3	3	4	3	4	4	4	2	2,17	4	4	4	2	2,67	6	4	3	2	2
								1																											
2	2	2	2	2	2	3	2		2	2	2	2	5	6	2		2	2	2	5	3	4	3	1	1,83	4	2	4	3	3,00	6	3		2	2
1	1	1	1	1	2	2	2		4	2	2	2	6	6	1		1	1	2	4	4	5	1	1	2,00	4	1	1	1	1,22	6	2		2	2
2	2	3	4	3	4	3	2		1	3	3	4	2	5	5		1	2	3	3	2	3	2	2	3,00	3	2	2	2	2,11	6	2		3	3
3	3	3	2	4	3	3	2	1	6	3	3	3	5	5	2	4	1	1	3	3	3	5	4	2	3,17	2	2	2	2	1,44	6	3	3	3	3
3	3	3	3	3	3	4	3	1	2	2	2	4	1	2	6		2	1	1	5	5	2	3	5	3,50	4	5	4	3	1,78	6	2		3	4
3	3	4	2	1	2	2	1	1	4	3	3	3	3	3	4	4	3	3	3	3	3	3	3	4	3,50	3	3	3	4	3,67	6	3	3	3	4
2	3	3	4	2	2	1	2		4	3	2	2	3	3	4		2	2	2	3	3	4	2	4	3,17	3	3	2	1	2,56	4	3		2	4
2	3	2	2	1	2	3	3		3	3	5	2	3	2	4	2	2	3	3	5	3	3	2	5	3,67	5	2	2	2	1,89	5	3	3	3	4
2	2	3	2	1	2	3	1	1	4	2	2	2	4	3	3	2	1	1	2	5	2	3	1	4	2,83	4	1	2	2	2,56	6	2	2	2	4
3	3	2	1	1	1	2	1	1	2	1	2	2	6	4	1	4	2	1	2	3	3	3	2	3	1,83	4	2	2	2	1,89	6	2	3	2	2
2	2	2	2	2	2	2	2		3	4	4	4	4	4	3		4	4	4	4	4	4	4	3	3,50	4	4	4	3	3,33	3	4		4	3
3	3	4	4	2	3	2	2	1	1	3	1	3	2	6	5	4	2	3	3	2	5	6	3	1	2,33	3	2	1	2	1,67	6	4	4	2	2
1	2	1	1	1	1	1	1	1	2	2	3	2	5	4	2	2	2	2	2	4	2	3	2	3	2,33	5	3	3	2	1,78	6	2	2	2	2
4	4	4	5	5	3	3	1	1	2	1	2	4	5	6	2	4	1	2	2	4	3	3	2	1	2,00	5	2	2	3	1,78	6	2	4	2	2
2	1	4	2	1	1	2	2	1	4	2	1	2	5	4	2		1	1	2	5	1	2	2	3	2,33	4	1	2	1	1,44	6	2		2	3
3	3	3	3	3	3	3	3		4	3	3	3	3	3	4		3	3	3	3	3	3	3	4	3,50	3	3	3	4	3,67	6	3		3	4
3	2	2	1	2	2	3	2	1	1	2	2	3	3	4	4		2	2	2	2	2	4	2	3	2,50	5	3	3	2	1,44	6	3		2	3
2	2	2	3	3	3	3	2	1	1	1	1	2	5	6	2	2	2	2	2	5	3	2	2	1	1,33	3	2	2	2	2,11	6	2	3	1	1

4	3	5	3	4	2	3	3	1	4	2	3	5	4	5	3	4	4	2	3	1	5	6	4	2	3,17	4	4	3	1	2,33	5	4	4	3	3
3	3	3	2	3	2	3	1	1	1	1	3	1	6	6	1	5	1	1	1	6	4	3	1	1	1,33	2	1	2	1	1,11	6	2	2	2	1
4	4	4	2	3	3	3	2	1	4	2	3	3	2	5	5		2	1	3	3	5	4	3	2	3,17	3	2	2	2	2,00	5	3		3	4
2	3	2	4	3	3	3	2	1	4	2	3	2	3	4	4	4	2	2	2	4	3	2	2	3	3,00	5	2	2	2	2,00	5	2	5	2	4
4	3	4	3	3	3	2	2	1	3	2	3	4	4	4	3	2	2	2	3	3	4	4	4	3	3,00	5	2	2	3	2,89	6	3	3	3	3
3	2	4	2	2	5	3	2	1	6	1	1	2	5	6	2		2	2	2	3	2	3	2	1	2,17	4	2	2	1	1,67	5	2		1	3
4	3	2	1	1	1	3	1	1	3	1	3	4	3	3	4		2	2	3	4	4	4	3	4	3,17	5	3	3	1	1,89	6	3		3	4
1	2	5	1	1	1	1	1		2	2	2	2	5	6	2	4	1	1	1	6	4	6	1	1	1,83	5	3	3	2	1,78	6	2	4	2	2
4	4	5	5	2	4	4	4	1	1	2	2	3	4	4	3	2	1	1	2	5	4	4	2	3	2,33	5	2	2	2	1,56	5	2	3	2	2
5	5	5	5	5	5	5	5		1	6	6	6	6	6	1	1	6	6	6	6	6	6	6	1	3,50	6	6	6	3	2,67	4	5	5	6	1
4	3	3	2	1	1	2	1	1	2	2	4	3	3	4	4	2	2	2	5	3	5	2	3	3	3,00	4	2	2	2	2,00	6	3	2	3	3
3	3	3	4	3	3	4	3	1	5	1	1	3	3	4	4	2	2	3	4	4	4	2	3	3	2,83	4	2	2	2	3,56	6	3	3	2	4
3	1	4	2	1	3	2	1	1	2	1	2	2	1	5	6	2	3	4	3	2	5	6	2	2	2,50	3	1	3	4	2,78	6	4	2	2	3
3	3	2	1	2	2	2	2		1	1	2	2	3	4	4	1	2	2	2	6	1	5	2	3	2,17	5	2	2	2	1,56	6	2	2	2	3
5	5	5	2	3	2	2	1	1	2	1	5	2	3	6	4		2	3	5	1	6	5	3	1	2,50	3	1	2	2	1,89	6	4		3	2
3	4	5	4	3	4	4	4	1	4	4	3	5	5	4	2	2	2	4	6	3	2	4	2	3	3,50	4	3	4	3	3,00	3	3	3	4	3
3	3	4	3	3	3	3	3	1	2	2	4	5	4	5	3	4	2	2	3	3	5	3	4	2	3,00	3	2	2	2	2,00	6	3	3	4	2
3	2	4	2	3	3	2	3	1	1	3	3	5	3	3	4	2	2	2	2	4	5	4	2	4	3,33	5	3	3	3	2,22	5	3	3	4	3
1	1	1	2	3	2	3	3	1	1	2	2	3	3	4	4	2	2	2	2	4	2	3	2	3	2,50	4	2	2	2	1,22	6	2	3	2	3
2	3	2	2	3	4	3	2	1	3	4	5	6	2	4	5	4	4	3	5	3	3	2	1	3	4,33	4	3	3	4	4,11	3	3	3	5	4
3	3	2	1	2	3	2	2		6	2	3	3	3	5	4		2	3	4	3	4	5	4	2	3,33	6	5	4	2	2,00	6	4		3	4
3	3	4	1	1	1	3	2	1	2	2	2	3	4	5	3		3	3	3	3	3	3	2	2	2,33	3	3	2	3	2,67	5	3		2	2
5	4	5	4	4	4	4	4		3	4	2	4	4	4	3		3	3	4	2	5	5	4	3	3,17	5	4	3	3	2,89	5	4		3	3
4	4	4	3	5	3	3	4	1	1	2	5	4	3	4	4	5	2	3	3	1	6	6	6	3	3,17	6	4	4	2	2,00	6	5	4	4	3
5	5	5	4	5	3	3	1	1	4	2	3	4	5	6	2	2	2	3	2	4	6	6	6	1	2,67	2	2	2	1	1,33	5	4	2	3	2
4	4	3	4	5	3	4	3	1	4	3	3	2	3	5	4		2	2	4	2	5	3	3	2	3,00	3	3	2	2	1,78	6	3		3	3
								1																											
2	2	3	1	2	2	3	1	1	4	2	3	3	3	3	4		2	2	2	4	3	2	3	4	3,33	4	3	3	2	2,33	6	2		3	4
4	2	3	1	3	1	3	2	1	1	2	1	2	6	6	1		2	2	2	4	2	3	2	1	1,33	4	3	3	3	2,11	6	2		2	1
1	1	4	1	1	1	1	1	1	1	1	1	1	6	6	1	1	1	1	1	4	1	1	1	1	1,00	1	1	1	1	1,00	5	1	2	1	1
3	3	3	3	3	3	3	3	1	6	3	6	4	2	3	5		2	1	3	2	3	3	4	4	4,67	3	2	2	2	2,78	5	3		4	5
3	2	2	2	2	2	3	1		4	2	3	3	3	3	4	4	2	2	2	4	1	1	2	4	3,33	5	2	2	2	2,00	5	2	3	3	4
								1																											
3	3	4	3	2	3	3	3	1	3	2	3	3	3	3	4	2	2	2	4	3	4	5	3	4	3,17	4	2	3	3	3,00	6	3	4	3	4
4	4	4	2	3	2	3	2		5	2	2	3	3	3	4	2	3	2	3	3	5	4	4	4	3,33	4	3	2	2	2,11	6	4	3	2	4
4	3	5	4	2	3	4	3	1	2	2	3	2	4	5	3	2	1	1	2	3	5	1	2	2	2,33	3	2	2	1	1,00	6	2	2	2	2
3	3	4	3	3	3	3	3		4	3	2	3	3	3	4	2	2	3	3	4	4	3	3	4	3,33	4	3	2	2	2,78	6	3	3	3	4
4	3	4	2	2	3	2	2	1	2	1	2	3	3	4	4	2	3	2	2	2	5	4	3	3	2,50	3	2	2	2	2,00	6	3	2	2	3
1	2	3	3	2	2	3	2	1	3	1	3	4	2	3	5	2	3	3	2	2	2	3	3	4	3,33	3	3	2	4	4,00	3	3	3	3	4
2	1	2	1	1	1	1	1		2	2	2	2	4	5	3	2	2	1	2	5	3	2	2	2	2,17	4	2	2	1	1,11	6	2	2	2	2
3	2	3	1	1	2	3	1	1	3	2	2	4	3	6	4	2	1	1	3	4	2	5	1	1	2,67	3	1	2	2	1,11	6	2	3	3	3
2	2	1	2	3	3	3	2	1	4	3	1	2	2	4	5		3	3	2	3	4	1	2	3	3,00	3	3	4	4	3,89	3	3		2	4
1	2	2	2	1	2	3	2	1	4	2	3	3	4	5	3	2	2	2	2	5	3	2	2	2	2,83	4	2	2	2	1,67	5	2	2	3	3
2	2	1	1	1	2	3	1		1	2	1	2	5	6	2	2	1	2	2	4	2	2	1	1	1,50	4	2	2	2	1,78	6	2	2	2	1
2	3	1	1	1	1	2	1	1	1	2	2	2	5	4	2		2	2	2	3	3	3	3	3	2,00	4	2	2	2	1,44	6	3		2	2
3	3	4	3	5	5	2	3	1	3	3	2	3	5	5	2	2	2	2	2	3	2	4	1	2	2,50	4	2	2	3	2,00	6	2	3	3	2
2	2	2	3	2	3	3	3	1	4	2	3	4	3	5	4		2	1	2	5	2	2	2	2	3,17	3	2	2	2	2,33	6	2		3	3
1	1	1	1	1	2	3	1		1	2	2	2	4	4	3	2	2	1	2	5	1	1	1	3	2,17	3	1	1	1	1,11	6	1	2	2	2
								1																											
								1																											
3	2	1	3	3	3	3	2	1	3	3	2	2	5	5	2		1	1	2	4	1	1	1	2	2,33	4	2	2	1	1,78	6	1		2	2
								1																											
								1																											
3	4	5	5	1	5	5	5	1	4	3	4	3	3	3	4	4	3	4	3	2	4	6	4	4	3,67	5	5	4	3	3,67	5	4	4	3	4
1	1	1	1	1	2	2	1	1	1	2	2	3	6	5	1		1	1	2	5	2	5	2	2	1,83	3	2	2	2	1,89	6	2		2	1
1	1	1	1	1	1	1	1		5	1	1	1	1	2	6	5	1	1	1	1	1	1	1	5	3,17	1	1	1	4	4,33	2	2	2	1	5
3	3	4	3	3	2	4	3	1	5	2	3	5	1	4	6		3	4	2	5	3	1	3	3	4,00	3	3	4	4	3,67	3	3		3	5
4	2	5	5	2	4	5	2	1	5	1	4	1	2	3	5	5	1	3	2	1	1	3	2	4	3,33	5	3	2	3	1,33	6	3	4	2	5
1	3	4	1	1	3	3	1	1	5	1	3	5	2	3	5	4	1	2	2	3	2	4	2	4	3,83	4	2	2	1	1,44	5	2	3	3	5
4	4	4	3	3	3	3	2	1	1	1	1	2	4	5	3	2	2	4	2	3	5	5	5	2	1,67	4	4	4	3	1,67	6	4	4	1	2
3	3	2	3	3	2	3	2	1	4	3	3	3	4	4	3	2	2	2	3	4	4	4	3	3	3,17	4	2	3	4	3,11	6	3	3	3	3
3	2	3	3	2	3	3	2	1	4	2	3	4	3	4	4	2	2	2	2	5	1	2	2	3	3,33	4	2	2	1	2,00	6	2	2	3	4
3	3	3	2	4	3	3	1	1	6	1	2	2	6	4	1		2	3	2	3	5	2	3	3	2,50	3	2	2	2	1,89	6	3		2	3
3	3	2	3	3	3	3	3	1	6	2	2	3	5	6	2		2	2	2	3	4	4	2	1	2,67	6	4	5	2	2,00	6	3		2	3
4	4	3	2	3	3	2	3		6	2	3	2	2	6	5	2	4	2	3	3	5	6	3	1	3,17	5	4	2	2	1,78	6	4	3	2	4
2	3	3	3	1	2	3	1		1	2	3	4	5	6	2	2	2	2	3	3	3	3	2	1	2,17	4	4	3	3	1,67	6	3	2	3	1
3	4	3	2	1	4	4	1	1	1	2	2	3	6	5	1	2	2	2	4	3	4	5	3	2	1,83	4	2	2	1	1,67	6	3	2	2	1
3	2	2	1	3	1	2	1	1	2	1	2	3	6	5	1		3	3	2	3	3	4	4	2	1,83	3	3	2	2	1,89	6	3		2	2
4	4	3	1	3	1	3	1		1	2	3	3	3	5	4		1	3	1	3	5	6	3	2	2,50	4	4	2	2	2,33	5	3		3	2
5	5	5	3	2	2	3	2	1	1	3	2	3	6	3	1		3	5	5	2	3	3	5	4	2,33	3	3	3	3	3,56	6	4		3	2
2	2	2	1	1	1	3	3	1	1	2	2	3	6	5	1	2	2	2	3	3	3	5	3	2	1,83	4	2	2	1	1,44	6	3	2	2	1
4	3	3	1	1	1	3	1	1	3	2	1	4	3	5	4	4	2	1	2	4	2	2	2	2	2,67	4	3	4	1	1,11	6	2	3	2	3
3	3	5	4	4	1	3	2	1	3	2	4	4	4	3	3	4	2	2	3	3	5	3	3	4	3,33	4	3	3	3	3,44	5	3	3	3	3
5	5	5	3	3	2	2	2	1	4	2	4	3	5	5	2		3	1	3	3	5	1	3	2	2,83	5	3	2	2	2,11	5	3		3	3
1	1	1	1	1	1	1	1	1	1	2	2	2	5	4	2	4	2	2	3	3	4	3	3	3	2,00	5	1	2	1	1,89	6	3	4	2	2
2	3	3	4	4	3	4	4	1	4	3	2	4	3	4	4	2	2	2	3	4	3	3	2	3	3,33	3	2	2	3	2,89	5	3	3	3	4
3	3	3	3	3	3	3	3	1	4	3	3	3	3	3	4	2	3	3	3	3	3	3	3	4	3,50	3	3	3	3	3,56	3	3	3	3	4
2	2	1	1	2	2	2	2		1	1	1	1	6	5	1	1	2	1	2	3	1	2	2	2	1,17	4	2	3	2	2,11	6	2	3	1	1
3	2	2	2	2	3	3	2		6	2	2	2	5	3	2	2	2	2	3	4	2	6	2	4	3,00	4	2	2	2	2,22	5	3	2	2	4
4	3	2	3	3	2	2	1	1	3	3	4	6	5	4	2	4	2	3	1	1	6	3	2	3	3,50	4	2	2	1	2,22	6	3	3	4	3
4	4	4	1	1	3	2	2		4	2	2	2	3	4	4		3	1	2	3	2	5	2	3	2,83	4	4	3	3	3,33	6	3		2	4
1	2	2	1	2	3	3	1	1	2	2	2	3	5	6	2	2	2	2	3	5	3	4	3	1	2,00	2	1	2	1	2,00	6	3	2	2	2
4	4	3	2	3	2	2	2	1	5	4	4	3	4	5	3	2	3	2	3	3	4	4	2	2	3,50	4	3	2	2	1,22	6	3	3	4	3
								1																											
								1																											
								1																											
								1																											
3	2	2	1	3	2	2	1	1	4	2	2	3	4	3	3		2	2	4	4	2	3	3	4	3,00	4	3	3	3	2,33	6	3		2	4
4	3	4	2	1	2	3	1	1	1	2	3	3	3	3	4	2	4	3	3	3	4	3	5	4	2,83	5	3	3	1	2,44	6	4	2	3	3
								1																											
1	2	1	1	1	2	1	1		4	3	4	3	3	4	4	2	2	3	3	4	4	4	4	3	3,50	5	2	3	2	2,67	5	3	2	3	4
2	2	2	2	1	1	3	2	1	1	1	3	3	3	6	4	2	1	1	1	2	1	1	1	1	2,17	2	1	1	1	1,78	6	2	3	2	2
5	4	3	2	1	2	4	3	1	5	3	6	4	4	2	3	2	5	3	6	2	5	3	4	5	4,33	3	4	3	3	3,44	4	4	3	4	4
2	2	2	3	3	3	3	2	1	2	3	2	3	4	4	3	2	2	2	2	4	3	3	2	3	2,67	3	2	2	2	2,89	4	2	3	3	3
4	4	3	3	4	3	5	3	1	3	3	3	4	6	4	1		3	3	4	3	5	1	4	3	2,83	4	4	2	2	2,00	5	3		3	2
2	2	3	2	1	3	3	1	1	4	3	4	5	3	3	4	2	1	1	1	5	2	2	2	4	4,00	4	2	2	1	1,11	5	2	2	4	4
1	1	1	1	1	1	2	1	1	2	2	2	1	5	4	2	1	1	1	2	6	1	1	1	3	2,00	2	1	1	1	1,00	5	1	2	2	2
3	3	3	2	3	3	2	2	1	1	2	2	3	5	5	2		2	2	3	3	4	5	3	2	2,00	3	2	3	2	2,44	6	3		2	2
3	4	4	4	5	3	4	4		3	3	2	2	4	2	3		2	2	2	3	4	2	3	5	3,00	3	3	2	3	3,33	4	3		2	4
3	3	3	2	3	3	3	3	1	2	3	3	3	2	4	5	2	2	2	5	3	2	4	3	3	3,17	5	3	2	2	2,11	6	3	3	3	3
3	3	4	2	3	3	3	2	1	1	1	3	4	5	5	2	2	2	3	3	4	5	5	4	2	2,17	3	2	2	1	1,67	6	4	2	3	2
4	3	4	2	2	1	1	1	1	2	3	3	3	2	6	5	2	3	3	3	4	5	5	3	1	2,83	4	3	2	3	2,44	6	4	3	3	3
4	3	3	2	3	3	3	3	1	2	2	4	4	3	2	4	2	2	2	5	5	6	6	5	5	3,50	3	3	2	2	2,56	6	4	3	3	4
2	2	2	4	3	3	3	3		4	2	2	2	3	4	4	2	2	2	3	4	3	5	2	3	2,83	3	2	2	2	1,44	5	3	2	2	4
3	3	4	2	3	4	3	3	1	2	2	2	4	3	4	4	2	2	3	4	3	4	6	5	3	2,83	4	2	2	2	1,78	6	4	3	3	3
1	3	3	2	4	3	3	5		2	2	4	4	6	6	1	4	4	1	3	3	4	2	4	1	2,33	4	4	2	1	1,89	5	3	4	3	1
4	4	4	2	3	3	3	3	1	4	2	3	1	4	4	3	4	2	2	2	2	3	3	2	3	2,67	4	2	3	3	3,44	6	3	2	2	3
								1																											
3	3	3	3	3	3	3	3	1	5	2	1	4	3	1	4		1	1	1	2	1	2	4	6	3,67	2	2	2	3	4,00	6	2		2	5
4	3	3	2	2	3	2	2	1	1	2	5	3	4	3	3	2	2	1	2	6	6	4	3	4	3,00	4	4	4	6	3,44	6	3	3	3	3
1	1	2	1	1	1	2	1		3	3	3	3	5	4	2	4	2	2	2	5	5	4	3	3	2,83	5	5	3	1	2,11	6	3	3	3	3
1	1	3	3	1	3	1	1	1	4	2	2	2	5	6	2	4	1	1	2	4	2	2	1	1	2,17	4	2	1	1	1,22	6	2	3	2	2
2	2	2	1	1	1	1	1	1	1	1	1	1	6	6	1	1	1	1	1	6	1	1	1	1	1,00	5	2	1	1	1,00	6	1	2	1	1
1	1	2	1	2	3	3	3		2	2	2	3	3	5	4	2	2	2	2	5	2	4	3	2	2,50	5	2	3	3	2,11	5	2	2	2	3
4	4	4	3	4	4	4	4	1	4	2	3	4	5	5	2	2	2	2	2	2	4	5	5	2	2,83	3	2	2	2	1,78	6	4	4	3	3
4	5	5	5	5	5	5	5	1	1	1	1	1	6	5	1		2	1	2	3	4	2	2	2	1,17	4	3	4	3	3,11	5	2		1	1
4	3	3	3	3	4	3	2	1	6	2	5	5	3	3	4		2	1	2	3	4	3	3	4	4,33	3	2	2	1	1,67	5	3		4	5
4	4	4	4	4	4	4	4	1	5	2	2	5	2	5	5	2	2	2	2	3	5	3	2	2	3,50	4	2	2	2	2,11	6	3	4	3	4
3	3	2	2	1	3	3	3	1	1	2	2	5	4	5	3	1	1	1	2	5	2	6	2	2	2,50	3	1	1	1	1,00	6	2	2	3	2
3	2	3	1	2	2	2	2	1	1	2	1	2	6	4	1	2	2	2	2	5	3	4	4	3	1,67	3	2	2	2	2,00	6	3	2	2	2
4	3	1	1	1	1	1	2	1	2	3	3	4	5	5	2	2	1	1	6	3	3	2	2	2	2,67	4	2	2	1	1,78	6	3	2	3	2
3	3	3	2	2	2	2	2	1	1	2	2	3	5	5	2	2	2	2	2	4	4	3	3	2	2,00	4	3	3	3	2,78	6	3	2	2	2
5	5	5	5	4	4	4	3	1	2	2	4	3	3	3	4	2	3	2	3	3	6	1	5	4	3,17	4	3	4	2	2,11	6	3	4	3	3
5	4	5	1	2	3	3	3	1	5	2	3	4	3	3	4	2	2	2	3	3	3	5	4	4	3,67	4	2	2	3	2,22	6	3	2	3	4
1	3	2	1	3	3	3	3	1	2	1	1	2	5	4	2	2	1	1	1	5	2	2	1	3	1,83	2	1	2	2	2,33	5	1	3	1	2
3	2	3	3	2	1	2	2	1	2	5	1	2	3	1	4		1	1	1	2	6	5	2	6	3,33	4	2	2	2	2,11	5	3		3	4
1	1	1	1	1	2	1	1	1	3	2	2	2	3	5	4	2	2	1	1	4	3	4	1	2	2,50	3	3	2	2	2,00	6	2	3	2	3
4	3	3	4	4	3	4	3	1	2	4	3	4	5	4	2		3	3	4	4	5	5	5	3	3,00	4	3	4	3	3,44	4	4		4	2
4	5	5	5	5	5	5	5	1	2	1	3	2	5	6	2		1	1	3	3	6	3	1	1	1,83	3	1	4	1	1,00	5	3		2	2
4	4	5	1	1	1	1	2	1	1	2	2	2	5	6	2	2	1	1	4	5	3	3	2	1	1,67	6	6	5	3	1,33	6	2	3	2	1
2	3	3	1	3	3	3	2		5	2	2	1	4	1	3		3	4	5	3	2	3	2	6	3,17	3	3	2	4	3,33	5	3		2	5
3	2	3	2	2	3	3	2	1	2	2	2	3	5	6	2	4	2	2	1	2	4	5	2	1	2,00	3	2	3	2	1,89	5	3	3	2	2
3	3	4	4	4	3	3	1	1	1	2	4	5	6	3	1		2	2	4	4	2	6	2	4	2,83	4	3	2	3	3,22	6	3		4	2
3	3	4	3	2	3	3	3		2	3	2	3	5	6	2	2	2	1	2	5	5	4	2	1	2,17	4	3	3	2	1,78	6	3	3	3	2
3	3	4	5	1	2	4	3	1	1	1	2	2	6	6	1	4	2	1	1	1	3	6	2	1	1,33	4	1	2	3	3,22	4	3	4	2	1

2	2	2	2	2	2	3	2	1	1	1	1	2	4	4	3	1	1	1	2	6	2	1	1	3	1,83	3	2	1	2	2,56	4	1	4	1	2
4	5	3	2	4	3	5	4	1	1	4	3	5	3	5	4	4	5	1	2	3	5	3	2	2	3,17	4	2	2	1	1,67	5	3	4	4	2
4	4	4	4	3	4	3	3	1	2	2	3	3	4	6	3		2	1	1	2	3	2	2	1	2,33	3	2	2	2	2,44	6	2		3	2
2	2	3	1	1	2	2	1		2	2	2	2	5	5	2	2	2	2	3	4	3	3	2	2	2,00	4	2	2	2	2,11	6	3	3	2	2
5	5	5	5	4	4	3	2	1	4	1	3	5	6	5	1	5	3	3	2	1	5	5	6	2	2,67	5	4	4	1	1,56	6	4	5	3	2
2	2	3	1	1	2	2	1	1	4	2	2	3	3	6	4	2	1	1	1	5	2	6	1	1	2,67	4	1	1	1	1,00	5	2	4	2	3
3	4	5	3	5	4	3	4		6	2	6	5	1	1	6	4	3	2	2	2	6	6	3	6	5,17	3	3	3	2	2,11	5	4	4	4	6
3	3	2	3	3	3	3	3	1	1	2	2	2	5	4	2	2	2	2	2	5	2	2	2	3	2,00	3	3	4	2	1,89	6	2	2	2	2
5	5	5	5	5	5	5	5	1	1	4	6	6	4	6	3	1	6	6	6	6	6	3	5	1	3,50	5	5	6	3	3,33	3	5	5	5	2
2	2	2	1	2	1	2	3	1	1	2	2	3	4	4	3	2	2	2	2	5	3	3	2	3	2,33	4	2	2	2	2,22	6	2	2	2	2
4	4	4	4	3	3	4	4	1	2	1	1	2	6	6	1	4	4	2	4	2	6	6	3	1	1,33	4	3	3	2	3,44	5	4	4	1	1
3	3	4	2	1	4	3	1	1	4	4	3	3	2	5	5	1	2	1	2	4	4	4	2	2	3,50	4	2	2	1	1,67	6	3	3	3	4
3	3	1	1	3	1	2	1	1	2	2	3	3	3	5	4	2	4	1	2	3	6	6	5	2	2,67	3	2	2	2	2,11	6	4	2	3	3
4	4	4	4	4	4	3	4	1	4	2	2	4	2	4	5	2	2	2	4	2	4	2	3	3	3,33	4	2	2	3	2,89	5	3	4	3	4
1	1	1	1	1	1	1	1	1	1	1	2	2	6	5	1	2	1	1	1	6	1	1	1	2	1,50	3	1	1	1	1,56	6	1	2	2	1
3	4	4	4	2	3	3	1	1	3	4	2	4	3	4	4	1	2	4	4	4	3	3	2	3	3,33	5	2	2	4	3,44	6	3	2	3	3
